# *Crataegus pentagyna* willd. Fruits, leaves and roots: phytochemicals, antioxidant and antimicrobial potentials

**DOI:** 10.1186/s12906-024-04430-4

**Published:** 2024-03-19

**Authors:** Akram Taleghani, Samira Eghbali, Roya Moghimi, Majid Mokaber-Esfahani

**Affiliations:** 1 Department of Chemistry, Faculty of Science, Gonbad Kavous University, Gonbad Kavous, Iran; 2 Department of Pharmacognosy, School of Pharmacy, Birjand University of Medical Sciences, Birjand, Iran; 3 Department of Organic Chemistry, Faculty of Chemistry, University of Mazandaran, Babolsar, Iran

**Keywords:** *Crataegus pentagyna*, HPLC-ESI-MS/MS, GC-MS, PCA, Phenolic compounds, Antioxidant capacity, Antibacterial activity

## Abstract

**Background:**

The hawthorn has recently been used as a popular herbal medicine in food applications and phytotherapy, especially for the cardiovascular system.

**Methods:**

In this study, phytochemicals were evaluated by LC-ESI-MS, GC-MS, and biological activity, including antioxidant (DPPH test) and antibacterial (broth dilution assay), in different extracts of *Crataegus pentagyna* fruit, leaf, and root.

**Results:**

Globally, 49 phenolics were tentatively identified using HPLC-ESI-MS/MS in the hydro-methanolic extract of the fruit (major apigenin, caffeoylquinic acid derivative, and 4-*O-*(3′-*O-*glucopyranosyl)-caffeoyl quinic acid), 42 in the leaf (major salicylic acid, naringenin-6-C-glucoside, and naringin), and 33 in the root (major naringenin-7-*O-*neohesperidoside, isovitexin-2″-*O-*rhamnoside, and 4-*O-*(3′-*O-*glucopyranosyl)-caffeoyl quinic acid). The major group compounds analyzed by GC-MS in petroleum ether extracts were hydrocarbons (63.80%) and fatty acids and their derivatives (11.77%) in fruit, hydrocarbons (49.20%) and fatty acids and their derivatives (13.85%) in leaf, and hydrocarbons (53.96%) and terpenes (13.06%) in root. All samples exhibited promising phytochemical profile (total phenol, flavonoid, phenolic acid, and anthocyanin), antioxidant and antibacterial capacities, especially in hydro-methanolic extract of fruit (210.22 ± 0.44 mg GAE/g DE; 79.93 ± 0.54 mg QE/g DE; 194.64 ± 0.32 mg CAE/g DE; 85.37 ± 0.13 mg cyanidin 3-glucoside/100 g FW; DPPH: 15.43 ± 0.65 µg/mL; MIC: 0.15–0.62 µg/mL; and MBC: 0.62–1.25 mg/mL), followed by the leaf and root extracts, respectively. The PCA and heatmap analysis results distinguished metabolite profile differences for samples.

**Conclusion:**

The results of the present work provide scientific support for *C. pentagyna* as antimicrobial agents and natural antioxidants in human health and food preservation.

**Supplementary Information:**

The online version contains supplementary material available at 10.1186/s12906-024-04430-4.

## Introduction

Oxidative stress caused by overproduction of free radicals and reactive oxygen species that results in the development of several diseases such as diabetes, alzheimer’s disease, parkinson’s disease and cardiovascular conditions such as atherosclerosis, stroke and high blood pressure [1, 2]. Also, infectious diseases such as measles, flu, HIV, COVID-19, strep throat and salmonella are disorders caused by organisms such as viruses, bacteria, fungi or parasites [3]. Infectious diseases resistance to antibiotics are 3rd common cause of death worldwide after cardiovascular diseases [4]. So, there is a continuous need for research and investment in the field of new drugs and food preservatives with lower toxicity and higher efficacy. Recently, pharmaceutical and food industries have utilized medicinal plants as sources of bioactive substances, particularly phenolic compounds with a wide range of pharmacological activities [5]. Most research found that phenolic compounds specially flavonoids exert antibacterial activity via damaging microbial cell membranes and inhibiting microbial enzymes and gene expression [6] and antioxidant activity via decreasing enzymatic activity of oxidases [7]. Hawthorn, the common name for more than a thousand species of plants in the genus *Crataegus* and family Rosaceae (subfamily Maloideae). The genus of *Crataegus* is primarily found in Asia, Europe, and North America and is recommended by the World Health Organization as a medicinal and food ingredient in several countries. This genus is native to northern temperate regions and consists of 15- to 18-foot-tall trees and deciduous shrubs [8]. Flowers, fruits, leaves, stems, and roots of *Crataegus* species have been recommended in modern and traditional medicine as cardiotonic, antispasmodic, diuretic, anti-atherosclerotic, and hypotensive agents [9, 10]. In terms of biological activity, proanthocyanidins and flavonoid glycosides are the most important compounds in hawthorn. In the leaves, flowers, and fruits of hawthorn, known phenolic compounds, such as quercetin, isoquercetin, rutin, hyperoside, epicatechin, chlorogenic acid, and protocatechuic acid, may be excellent sources of antioxidants [9–11]. Variations in genetics, maturity of plant organs, collection regions, processing methods, and preharvest and postharvest environmental conditions may influence the chemical compound content of plant organs [12, 13]. In Iran’s flora, *Crataegus pentagyna* subsp. *elburensis* is the most common cultivar of hawthorn. This plant is primarily utilized as an antiarrhythmic and cardiovascular disease preventative. The presence of polyphenols, such as flavonoids, phenolic acids, and proanthocyanidins, in the species may be primarily responsible for these effects [14]. Earlier phytochemical investigations of *C. pentagyna* from different origins have revealed the identification of different compounds such as gallic acid, caffeic acid, and chlorogenic acid in fruit, pulp and seed of Iranian species [15, 16]; hyperoside, rutin, isoquercitrin, sexangularetin-3-*O*-glucoside, isoorientin, isoorientin-2-*O*-rhamnoside, isovitexin, orientin, orientin-2-*O*-rhamnoside, vitexin, and vitexin-2-*O*-rhamnoside in leaves of Austrian species [17]; coumaric acid, chlorogenic acid, caffeic acid, ferulic acid, quercetin 3-*O*-glucoside (isoquercetin), quercetin, quercetin 3-*O*-rutinoside (rutin), (-)-epicatechin, kaempferol 3-*O-*glucoside, hyperoside, apigenin, cyanidin 3-*O*-glucoside, luteolin and procyanidins B1 and B2 in fruits, flowers and leaves of Serbian species [18]; and a number of flavonoid aglycones, flavonoid *O*- and *C*-glycosides, organic and phenolic acids and proanthocyanidins in leaf, flower and fruit of Romanian species [19]. Previous studies showed that these phytochemicals have been linked to the health-promoting effects of this species, including cardiovascular system influence, antioxidant, anti-cancer, antimicrobial, anti-inflammatory and antihypercholesterolemic activities [9, 20]. As an effective method with high sensitivity and resolution, liquid chromatography coupled with electrospray ionization tandem mass spectrometry (LC–ESI-MS/MS) is widely used for plant metabolomics analyses and species discrimination. To our knowledge, there are no reports comparing the chemical profile and biological activities of different morphological parts of *C. pentagyna*. We investigated the chemical composition, total phenol, total flavonoid, total phenolic acid, total anthocyanin, antioxidant, and antibacterial activities of *C. pentagyna* fruits, leaves, and roots collected in Golestan province of Iran. Using gas chromatography coupled with mass spectrometry (GC-MS) and LC-ESI-MS/MS techniques, the chemical profile of petroleum ether and hydro-methanolic extracts was determined.

## Materials and methods

### Chemicals

All chemicals were purchased from Sigma (St. Louis MO, USA), including gallic acid (purity = 97.5%), caffeic acid (98%), quercetin (95%), cyanidin-3-glucoside (≥ 95.5%), butylated hydroxytoluene (BHT) (≥ 99%), hydrochloric acid, sodium hydroxide, sodium molybdate, sodium carbonate, aluminum chloride, sodium acetate, potassium chloride, potassium acetate, Folin–Ciocalteu reagent, 1,1-diphenyl-2-picrylhydrazyl (DPPH) (≥ 95%), methanol, petroleum ether, and formic acid (≤ 100%) and etc. Quality of all chemicals used in LC–MS/MS and GC-MS were analytical grade. Aqueous solutions were also prepared using deionized water. Microorganism cultures of Gram-negative *Escherichia coli* (ATCC 25,922) and Gram-positive *Staphylococcus aureus* (ATCC 9144) were obtained from Iranian microbial collections, Pasteur Institute of Iran.

### Plant samples

The essential factors for growth of plant are water, sunlight, heat, and topographical conditions in the planting region. Research has displayed that the optimum climate conditions for *C. pentagyna* growing involve 300–400 mm of annual precipitation, 8–12.5 °C of annual mean temperature, 2300–3200 °C of annual cumulative temperature, 2300 h of annual sunshine, and soil pH from 6.0 to 7.0. The highest growth rate occurs in the orchard between the ages of one and five years. Identification of optimal harvesting season is necessary to ensure contents of desired bioactive compounds. Therefore, around 500 g of fruits, leaves, and roots without damage of *Crataegus pentagyna* Willd. from one plant population were collected from Farsian, Galikesh, Golestan province, Iran (37°16’01.6"N 55°26’09.5"E) in September 2020 (early maturity stage) at the highest content of phenolic compounds [16, 18]. Samples was verified by Dr. Ali Satarian and the voucher number (803,892) was deposited in the Herbarium of Gonbad Kavous University, Gonbad, Iran.

### Preparation of plant extracts

Fresh fruits, leaves, and roots were air-dried and ground separately using a mortar. One gram of defatted powder with petroleum ether (3 × 20 mL) was extracted thrice separately in the dark for two hours with water–methanol and water–ethanol 80% (3 × 20 mL). Under reduced pressure, the filtrate extracts were evaporated at 40 °C on a rotary evaporator (Heidolph, Laborota 4000, Schwabach, Germany) to dryness and then freeze-dried. The dried extracts were stored at − 20 °C in the dark until further examination. In addition, the yield of constituents and the weight of dried extracts were determined [18].

### Estimation of phenolic profile

#### Total phenolic content (TPC)

The TPC of the extracts was determined using a modified version of Singleton and Rossi’s (1965) method [21], with gallic acid as the standard. To 500 µL of diluted samples, a mixture of 2.5 mL of Folin–Ciocalteu (0.2 N) reagent and 2 mL of Na_2_CO_3_ (75 g/L) was added. The absorbance of the samples was measured at 765 nm following 30 min of incubation at 45 °C. The results were given in milligrams of gallic acid per gram of dry extract (mg GAE/g DE).

#### Total flavonoid content (TFC)

Using a modified aluminum chloride colorimetric method [22], the TFC content of the samples was determined. In total, 0.5 mL of samples was combined with 0.1 mL of sodium acetate (1 M) and 0.1 mL of AlCl_3_ (10%) and incubated for 30 min at room temperature. At 415 nm, the absorbance of the reaction mixture was measured. The total flavonoid concentration was calculated as mg of quercetin equivalents (QE) per gram of dry extract.

#### Determination of total phenolic acid content (TPAC)

The total phenolic acid content of the extracts was determined by spectrophotometry at 474 nm [23]. A total of 1 mL of each extract was combined with 10 g of sodium nitrite and 10 g of sodium molybdate diluted in 100 mL of water, 2 mL of HCl (0.5 M), 3 mL of water, and 2 mL of NaOH (8.5% *w*/*v*) in this procedure. The results were reported in terms of mg of caffeic acid (CAE) per g of dry extract (mg CAE/g DE).

#### Determination of total anthocyanin content

The total anthocyanin concentration was determined using a method previously described [24]. At 530 nm, the absorbance of diluted extracts with 1% HCl in methanol (5:95, *v*/*v*) was measured. Using the following equation, the values were expressed as mg malvidin-3-glucoside equivalents per g of dry extract.

The results were expressed in milligrams of cyanidin-3-glucoside per 100 g of fresh weight (mg c3g/100 g FW).

### HPLC-PAD analysis

An analytical technique A KNAUER liquid chromatograph system with a photodiode array detector (Smartline PDA 2600) and a quaternary pump (Smartline Pump 1000) has been developed. The dissolved extracts in methanol: water 7:3 (approximately 2 mg/mL) were subjected to a gradient method on a 150 mm length × 4.6 mm inner-diameter C18 amide column (Varian, Darmstadt, Germany) at a flow rate of 1.0 mL/min and injection volume of 20 µL (water as solvent A and methanol as solvent B, including 0.05% trifluoroacetic acid). The elution gradient was 0–10 min with 10% solvent B, 10–35 min with 10–100% B, 35–45 min with 100% B, 45–50 min with 100–10% B, and 50–55 min with 10% B. The system was managed using the software EZ Chrom Elite. For testing the system’s dependability, 4-hydroxymethyl benzoate, uracil, benzophenone, and 4-hydroxy ethyl benzoate were injected into the HPLC [25, 26].

### LC-ESI-MS/MS analysis of phenolic compounds

Using a Waters Alliance 2695 HPLC system coupled to a micro mass quattro micro API mass spectrometer with an ion source, the flavonoids and other phenolics compositions of hydro-methanolic extracts from the fruit, leaf, and root of *C. pentagyna* were determined (ESI). The separation was carried out using a Supelco C18 (15 mm×2.1 mm×3 μm) column with a flow rate of 0.2 mL/min and an injection volume of 10 µL. Using a gradient method (acetonitrile + 0.1% formic acid as solvent A and water + 0.1% formic acid as solvent B), the samples were eluted. The elution gradient was 0–5 min with 10% solvent A, 5–10 min with 10–50% A, 10–16 min with 50% A, 16–20 min with 50–90% A, 20–24 min with 90% A, 24–26 min with 90–10% B, and 26–30 min with 10% A. The mass spectra were acquired using negative ionization and a range of 50 to 2000 *m*/*z*. The detection of peaks at 310 °C probe temperature and 3.5 kV probe voltage were monitored. The LC-MS data were analyzed using MZmine version 2.35 software [27].

### GC-MS analysis of essential compounds

Experiments were performed using an Agilent 6890 A mass spectrometer and an Agilent 5973 gas chromatograph. A total of 1 mL of the diluted extracts was injected into the HP_5_MS column with a length of 30 m, an inner diameter of 0.25 mm, and a thickness of 0.25 μm. As a carrier gas, helium at a flow rate of 1.0 mL/min was utilized. The column temperature was set at 50 °C for 3 min before increasing to 280 °C at a rate of 4 °C/min. The temperature of the GC injector and MS transfer line were set to 250 and 230 °C, respectively. MS detection utilized an ionization energy of 70 eV and a mass range of 50–550 *m*/*z*. By comparing their mass fragmentation patterns and retention times with the Wiley 7.0 and NIST libraries, the compound profiles were identified in petroleum ether extracts [28, 29].

### Antioxidant activit

#### DPPH radical-scavenging activity

Modifications were made to the previously reported method [30] to measure antioxidant activity. Briefly, 3 mL of diluted samples with concentrations of 10 to 200 µg/mL were mixed with 1 mL of 100 M methanolic DPPH solution. The absorbance of the mixture was measured at room temperature at 517 nm. DPPH radical scavenging capacity was calculated using the following formula:

% inhibition = [(A_0_ − A_t_)/A_0_ × 100].

Where, A_0_ is the absorbance of the blank (without extracts) and A_t_ is the absorbance of the extracts.

The antioxidant capacity of the samples was expressed as IC_50_. Using the regression equation, the IC_50_ values were determined as the concentrations of extracts that inhibit 50% of DPPH radicals. In addition, BHT served as a positive control.

### Estimation of antibacterial activity

In our laboratory, a Gram-negative (*Escherichia coli* ATCC 25,922) and a Gram-positive (*Staphylococcus aureus* ATCC 9144) bacteria were stored at 4 °C and used in this study. Iranian Research Organization for Science and Technology provided the examined bacteria. The microdilution broth technique [31] was used to evaluate the antimicrobial activity of *C. pentagyna* extracts. Two-fold serial dilutions of extracts in Muller Hinton broth (MHB) were prepared in a microtiter ranging from 0.078 to 20 mg/mL, and one million colony-forming units per milliliter were added to each well. Gentamicin and MHB were used as positive and negative controls, respectively. After 24 h of incubation at 35 °C, MIC was defined as the lowest concentration of the extracts with no visible bacterial growth. Also, MBC was determined as the lowest concentration of the extracts that was resulted in bacterial death.

### Statistics

Each experiment was performed three times under the same conditions. The results of each test and analysis were recorded as means and standard deviation. One-way ANOVA test was used to compare variance analyses, and the SAS software (least significant difference (LSD) test) was utilized to compare means (*p* < 0.05). The chromatograms of fruits, leaves and roots of *C. pentagyna* were pre-processed using M*Z*mine analysis software package. To perform further statistical analyses was performed through SIMCA software (version 14.1 Umetrics AB, Umea, Sweden), M*Z*mine exported the peak list data for each sample, which included the average retention time (RT), *m/z* values and peak intensity (height). The exported data were subsequently mean-centered and Pareto-scaled prior to multivariate statistical analysis to enhance the contribution from medium-sized features without inflating the noise from quiet areas of the chromatogram. Principal Component Analysis (PCA) and related biplot and loading plot was used for multidimensional data to identify metabolite differences between samples. Furthermore, heatmap analysis was performed using import-exciting clusters to better show differences between metabolites.

## Results and discussion

*C. pentagyna* extraction yield, quantitative evaluation of phenolic profile, and antioxidant power.

The chemical components of the fruit, leaf, and root of *C. pentagyna* were fractionated using ether petroleum, 80% aqueous methanol, and ethanol as extraction solvents. The yield of extracts varied between 2.4% and 9.6% for methanolic extracts, between 2.1% and 8.3% for ethanolic extracts, and between 0.9% and 3.6% for petroleum ether extracts. Due to the hydroxyl (-OH) and methoxy (-OCH_3_) groups in their molecular structures [32], phenolic compounds possess the ability to scavenge free radicals. The phenolic content of hawthorn varies by cultivar, species, geographical location, harvest time, method of chemical determination of phytochemicals, and extraction preparation conditions [33]. There are few studies on the phytochemical content of *C. pentagyna* compounds. In addition, there have been no reports of hawthorn root. In this study, the phytochemical content (phenol, flavonoid, phenolic acid, and anthocyanin) and antioxidant capacity (DPPH scavenging) of hydro-methanolic and hydro-ethanolic extracts of fruit, leaf, and root were evaluated and compared (Table 1). The results indicated that hydro-methanolic extracts contain more phytochemicals and have more antioxidant capacity than ethanol extracts. Fruit extract contained the highest concentrations of phenolic (210.22 ± 0.44 mg GAE/g DE), total flavonoid (79.93 ± 0.54 mg QE/g DE), total phenolic acid (194.64 ± 0.32 mg CAE/g DE), and total anthocyanin (85.37 ± 0.13 mg cyanidin 3-glucoside/100 g FW) among the tested extracts, followed by leaf and root extracts. The total content of phenol and flavonoid in methanolic extracts of fruit from various regions ranged from 69.12 to 186.72 mg GAE/g and 1.6 to 85.31 mg QE/g dry weight plant, respectively, as reported by other researchers [15, 32, 34–36]. In a different study, the TPC and TFC concentrations in methanolic leaf extracts were 206.94 GAE/g and 57.08 mg (+)-catechin/g, respectively [37, 38]. There was a strong correlation between TPC and DPPH reduction. The phenolic and flavonoid content of fruit, leaf, and root extracts increases their antioxidant activity. The fruit extract with the highest phenolic and flavonoid content exhibited the highest DPPH radical scavenging capacity (IC_50_ = 15.43 ± 0.65 g/mL), followed by leaf and root extracts (IC_50_ = 34.67 ± 0.14 g/mL and 60.72 ± 0.32 g/mL, respectively). Moreover, the fruit and leaf extracts exhibited potent activity comparable to the positive control butylate hydroxytoluene (BHT) (IC_50_ = 49.02 ± 0.2 g/mL), whereas the root extract was less active. Antioxidant activity of fruit and leaf extracts of *C. pentaegyna* from different regions have been already investigated. For the methanolic fraction of fruit, the IC_50_ values for DPPH radical scavenging activity range from 17.48 to 341.29 g/mL [32, 34, 36, 39]. Moreover, the DPPH inhibition of *C. pentagyna* leaf was quantified as 5708 g/mL in hydroacetonic extract and 2.34 M TE/g (micromoles of Trolox equivalents) in ethanolic extract, respectively [18, 37, 40].

**Table 1:** Phytochemical screen and antioxidant activity of fruit, leaf, and root of *C. pentagyna* (TP: total phenol; TF: total flavonoid; TPA: total phenolic acid; TAC: total anthocyanin)

Analysis	Hydro-Methanolic Extract			Hydro-Ethanolic Extract		
	Fruit	Leaf	Root	Fruit	Leaf	Root
TP (mg GAE/g)	210.22 ± 0.44 ^a^	132.25 ± 0.32 ^b^	102.46 ± 0.54 ^c^	189.49 ± 0.19 ^d^	112.33 ± 0.48 ^e^	93.19 ± 0.58 ^f^
TF (mg QE/g)	79.93 ± 0.54 ^a^	67.21 ± 0.54 ^b^	48.66 ± 0.71 ^c^	58.9 ± 0.34 ^d^	39.53 ± 0.58 ^e^	42.47 ± 0.34 ^f^
TPA (mg CAE/g)	194.64 ± 0.32 ^a^	98.43 ± 0.76 ^b^	79.34 ± 0.92 ^c^	123.21 ± 0.67 ^d^	86.54 ± 0.39 ^e^	51.43 ± 0.85 ^f^
TAC (mg cyanidin 3-glucoside/100 g FW)	85.37 ± 0.13 ^a^	20.45 ± 0.53 ^b^	1.32 ± 0.13 ^c^	75.94 ± 0.84 ^d^	16.45 ± 0.88 ^e^	0.56 ± 0.44 ^c^
DPPH assay, IC_50_ (µg/mL)	15.43 ± 0.65 ^a^	34.67 ± 0.14 ^b^	60.72 ± 0.32 ^c^	29.48 ± 0.33 ^d^	51.32 ± 0.73 ^e^	70.21 ± 0.12 ^f^
Extraction yield % (*w*/*w*)	9.6	6.9	2.4	8.3	6.4	2.1

Means with different superscript lowercase letters within the same row differ significantly (*p* < 0.05)

### Phenolic characterization using HPLC-PDA and LC/ESI-MS/MS

**Fig. 1:**
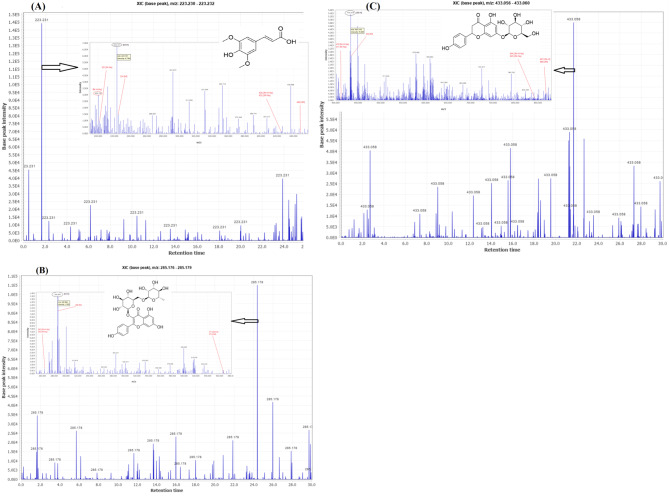
Extracted ion chromatograms (XIC) and corresponding mass adducts in the hydro-methanolic extracts of *C. pentagyna*. (**A**) chromatogram XIC of sinapic acid and mass adducts, m/z 223; (**B**) XIC of Kaempferol-3-*O*-rutinoside and mass adducts, m/z 285; and (**C**) XIC of naringenin-7-*O*-glucoside and mass adducts, m/z 433

Using HPLC-ESI-MS/MS in negative ionization mode, the phytochemical profile of hydro-methanolic extracts of the fruit, leaf, and root of *C. pentagyna* tentatively were identified. Fig. S1 A-C depict the HPLC-ESI-MS/MS and HPLC-PDA (280 nm, 330 nm, and 360 nm) fingerprints (Supplementary materials). LC-ESI-MS/MS chromatograms show total ion chromatograms of compounds (TIC). The extracted ion chromatograms (XIC) from total ion chromatograms were processed by MZmine analysis software package which can separate all chromatograms and compounds. Figure 1A-C illustrates the instances of extracted ion chromatograms (XIC). According to the ESI-MS/MS fragmentation pattern, molecular weights, matching mass adducts ([M-H]^−^, [2M]^−^, [2 M-H]^−^, [M-2 H]^−^,[M-2 H + Na]^−^ and [M-2 H + K]^−^) and published data, we identified 49, 42, and 33 phenolic compounds from the fruit, leaf, and root, respectively (Table 2) and (Supplementary materials: Table S1 and Fig S3-S5). Other hawthorn species (*Crataegus* spp.) in which these compounds have been previously identified are listed in Table 2 to compare with obtained results in our study. Phenolic compounds of *C. pentagyna* have been identified in a limited number of studies. Salmanian et al. [16]. determined the concentrations of gallic acid, caffeic acid, and chlorogenic acid in pulp and seed extracts of *C. pentagyna* from Iran. In Austrian *C. pentagyna* leaves, Prinz et al. [17]. isolated and identified hyperoside, rutin, isoquercitrin, sexangularetin-3-*O*-glucoside, isoorientin, isoorientin-2-*O*-rhamnoside, isovitexin, orientin, orientin-2-*O*-rhamnoside, vitexin, and vitexin-2-*O*-rhamnoside as flavones. In a separate study, Pavlovic et al. [18]. determined phenolics (*p*-coumaric acid, chlorogenic acid, caffeic acid, ferulic acid, isoquercetin, quercetin, rutin, (-)-epicatechin, kaempferol 3-*O-*glucoside, hyperoside, apigenin, cyanidin 3-*O*-glucoside, luteolin, procyanidins B1 and B2) during harvesting period in *C. pentagyna* fruits, flowers and leaves from Serbia. In addition, Alirezalu et al. [15]. quantified chlorogenic acid, vitexin, hyperoside, rutin, quercetin, and isoquercetin in the Iranian *C. pentagyna* fruit extract. Moreover, a number of 39 compounds (flavonoid aglycones, flavonoid *O-* and *C-*glycosides, organic and phenolic acids and proanthocyanidins) were identified in Romania *C. pentagyna* leaf, flower and fruit ethyl acetate extracts [19].

**Table 2:** Phenolic constituents identified in the hydro-methanolic extracts of the fruit, leaf, and root of *C. pentagyna* using HPLC-ESI-MS/MS.

No.	Compound	[M-H^]−−^ (m/z)	MS^2^ Fragments	R_t_ (min)/Intensitya (E^n^)/ Mass error (ppm)			Used Parts in Literature ^a^	Ref.
				Fruits	Leaves	Roots		
1	Chlorogenic acid glycoside: 4-*O*-(3′-*O*-glucopyranosyl)-caffeoyl quinic acid	515	353	0.91/7.2E^5^/ 0.22	0.93/2.7E^5^/ 0.23	0.93/4.8E^5^/ 0.23	F, L	[68]
2	Sinapic acid-*O*-hexoside	385	223, 208	1.1/7.3E^4^/ 1.21	-	1.3/1.2E^5^/ 1.17	F	[45]
3	Caffeoylquinic acid derivative	353	191, 179	1.3/8.2E^5^/ 0.27	-	-	F ^⁎^, L ^⁎^, Fl ^⁎^	[18]
4	Caffeoylquinic acid derivative	353	191, 179	1.3/6.9E^4^/ 0.27	1.4/4.4E^5^ 0.25	-	F, L ^⁎^, Fl ^⁎^	[12, 18, 33, 79]
5	Coumaroylquinic acid derivative	337	191	1.5/9.7E^4^/ -0.87	-	-	F^⁎^, Fl^⁎^, L^⁎^	[19, 68, 87]
6	Caffeoylquinic acid derivative	353	191, 179	-	-	1.6/6.3E^4^/ 0.24	F ^⁎^, L ^⁎^, Fl ^⁎^	[15, 18, 45, 51]
7	Coumaroylquinic acid derivative	337	191	-	1.7/1.1E^5^/ -0.81	1.6/2.0E^5^/ -0.83	F, L	[68]
8	Coumaroylquinic acid derivative	337	191	-	1.7/2.4E^5^/ -0.82	-	F ^⁎^, L	[18, 68]
9	Sinapic acid	223	208	-	1.9/1.3E^5^/ 2.54	1.8/6.7E^4^/ 2.57	F, Fl	[45]
10	Protocatechuic acid	153	109	2.5/1.5E^5^/ 0.64	-	-	F^⁎^, Fl^⁎^, L^⁎^	[19, 77]
11	Caffeic acid	178	178, 135	2.9/2.6E^5^/ -1.9	-	-	F ^⁎^, L ^⁎^, Fl ^⁎^	[18, 19, 45, 71]
12	Salicylic acid	137	93	4.3/1.3 E^5^/ 1.45	4.4/3.0 E^6^/ 1.39	4.3/1.3E^5^/ 1.45	F^⁎^, Fl^⁎^, L^⁎^	[19, 56]
13	Hydroxybenzoic acid derivative	137	93	4.3/1.2E^5^ 1.35	-	-	F^⁎^, Fl^⁎^, L^⁎^	[19, 45]
14	Hydroxybenzoic acid derivative	137	93	4.3/1.3E^5^/ 1.35	-	-	F	[45]
15	Ferulic acid	193	178, 149	4.7/1.2E^5^/ -0.31	-	-	F ^⁎^, L ^⁎^, Fl	[45–47, 57, 77]
16	Myricetin-3-*O*-(6″ galloyl) galactoside	631	479, 317	5.3/2.0E^5^/ -1.21	5.4/1.6E^5^/ -1.25	5.3/1.6E^5^/ -1.20	F	[56]
17	Coumaric acid	163	119	-	5.3/2.8E^5^/ 0.89	5.4/2.1E^5^/ 0.84	F ^⁎^, L ^⁎^, Fl ^⁎^	[18, 78]
18	Quercetin-3-*O-*rutinoside	609	271, 301	5.5/1.0E^5^ 0.45	5.5/2.4E^5^/ 0.43	-	L	[55]
19	Quercetin-3-*O-*(6″ galloyl) glucoside	615	463, 301	6.7/2.5E^5^/ -0.23	6.5/8.2E^4^/ -0.24	6.6/1.9E^5^/ -0.23	L	[56]
20	Quercetin-7,4′-dimethyl ether-3-*O-*rutinoside	637	491, 329	7.5/1.2E^5^/ 3.69	7.8/1.0E^5^/ 3.52	7.6/1.6E^5^/ 3.63	F	[56]
21	Quercetin-3-*O-*rhamnoside	447	301	8.4/7.2E^4^/ -2.71	8.2/3.0E^5^/ -2.63	-	L ^⁎^, Fl ^⁎^	[18]
22	Orientin or isoorientin glycoside (Orientin-2″-*O-*rhamnoside)	593	447	8.4/1.0E^5^/ 1.45	8.4/1.5E^5^/ 1.40	-	L ^⁎^, Fl ^⁎^	[12, 17]
23	Orientin or isoorientin glycoside (Isoorientin-2″-*O-*rhamnoside)	593	447	9.3/4.6E^4^ 1.44	-	-	Fl ^⁎^	
24	Vitexin-*O-*rhamnoside derivative	577	457, 431, 413, 311, 293	-	10.9/1.5E^5^/ 1.87	10.7/8.2E^4^/ 1.81	F, L ^⁎^, Fl ^⁎^	[48, 58–65]
25	Vitexin-*O-*rhamnoside derivative	577	457, 431, 413, 311, 293	-	10.7/1.3E^5^/ 1.87	10.9/5.7E^5^/ 1.86	-	[68]
26	Vitexin-*O*-glucoside derivative	593	473, 431, 413, 311, 293	10.6/1.2E^5^/ -2.41	-	-	F, L	[58, 60]
27	Naringenin-7-*O*-neohesperidoside	579	271	-	11.6/3.7E^5^/ 2.51	11.8/6.8E^5^/ 2.49	L	[67]
28	Vitexin-*O-*rhamnoside derivative	577	457, 431, 413, 311, 293	-	12.1/1.7E^5^/ 1.88	-	L, Fl	[42, 50, 64, 65]
29	Vitexin-*O*-glucoside derivative	593	473, 431, 413, 311, 293	12.8/5.3E^4^/ -2.44	-	-	L	[51, 64–66]
30	Myricetin-3-*O*-galactose	479	317	13.9/4.0E^4^/ -3.15	-	13.8/1.1E^5^/ -3.18	F	[56]
31	Quercetin-*O*-glycoside derivative	463	301	14.6/8.8E^4^/ 1.25	-	14.8/1.5E^5^/ 1.19	F, L ^⁎^, Fl	[12, 15, 18, 43, 46]
32	Quercetin-*O*-glycoside derivative	463	301	14.6/8.9E^4^/ 1.26	-	-	F ^⁎^, L ^⁎^, Fl ^⁎^, S	[15, 18, 42, 43, 50–54]
33	Quercetin-*O*-glycoside derivative	463	301	14.7/8.3E^4^/ 1.26	-	-	F, Fl	[1, 47–49]
34	Epigallocatechin gallate	457	305	15.4/9.5E^4^/ -0.86	15.2/1.4E^5^/ -0.81	-	F	[74]
35	Epicatechin gallate	441	289	15.9/9.3E^4^/ 0.56	15.8/1.1E^5^/ 0.52	-	F, L	[17, 49, 65]
36	Orientin or isoorientin	447	357, 327	16.9/8.5E^4^/ 1.23	-	-	L ^⁎^, Fl ^⁎^	[12, 17]
37	Orientin or isoorientin	447	357, 327	16.9/1.1E^5^/ 1.24	-	-	Fl ^⁎^	[17]
38	8-methoxykaempferol	315	300	17.1/2.0E^5^/ -1.45	-	-	Fl	[17, 51]
39	Eriodictyol-7-glucuronide	463	287	-	17.5/7.1E^4^/ 2.13	-	L, Fl	[17, 43]
40	Luteolin 7-*O-*glucoside	447	285	17.8/7.1E^4^ 0.93	17.6/2.1E^5^/ 0.91	-	F ^⁎^, L ^⁎^, Fl ^⁎^	[12, 17, 18, 42]
41	Luteolin-7-*O-*glucuronide	461	267	17.8/1.5E^5^/ 3.02	17.5/1.6E^5^/ 3.10	-	L	[43]
42	Apigenin-8-*C-*glucoside	431	341, 311	19.8/6.8E^4^/ 1.95	19.7/9.0E^4^/ 1.91	19.9/1.1E^5^/ 1.81	F^⁎^, L^⁎^, Fl^⁎^, S	[12, 15, 17, 42, 43, 51, 57]
43	Naringenin-6-*C-*glucoside	433	343, 313	20.5/3.9E^5^/ 2.34	20.6/5.3E^5^/ 2.30	20.4/9.0E^4^/ 2.35	L	[68]
44	Naringenin-7-*O-*glucoside	433	271	21.4/2.7E^5^/ 1.98	21.7/1.4 E^5^/ 1.93	21.5/1.4E^5^/ 1.95	L	[68]
45	Procyanidin B3 7-glucoside	739	721, 450, 435	-	22.9/1.6E^5^/ 2/54	-	F	[75]
46	Myricetin	317	300, 179, 151	23.5/1.5E^5^/ 1.31	23.5/8.8E^4^/ 1.19	-	F	[56]
47	5-*O-*methylmyricetin	331	315	23.7/1.3E^5^/ -0.32	23.9/1.3E^5^/ -0.37	23.7/1.2E^5^/ -0.33	-	[56]
48	Quercetin	301	151, 158	23.9/5.9E^4^/ 3.34	23.8/1.3E^5^/ 3.31	23.9/1.5E^5^/ 3.29	F^⁎^, L^⁎^, Fl	[18, 19, 42, 44, 45]
49	Catechin or epicatechin monomers	289	271, 245, 137	24.2/1.5E^5^/ 2.23	24.3/2.6E^5^/ 2.23	24.3/1.4E^5^/ 2.30	F^⁎^, L^⁎^, Fl	[19, 66, 71]
50	Kaempferol-3-*O-*rutinoside	593	285	24.4/9.0E^4^/ 0.45	-	-	F ^⁎^, L ^⁎^	[18]
51	Catechin or epicatechin monomers	289	271, 245, 137	24.4/7.6E^4^/ 1.69	24.4/1.0E^5^/ 1.61	24.6/1.6E^5^/ 1.58	F ^⁎^, L ^⁎^, Fl ^⁎^	[1, 18, 72, 73]
52	Luteolin	285	269	24.6/1.3E^5^/ 3.01	24.9/6.7E^4^/ 3.04	24.7/1.0E^5^/ 3.10	L	[42]
53	Kaempferol	285	-	24.6/2.9E^5^/ -0.82	24.9/7.7E^4^/ -0.73	-	F ^⁎^, Fl ^⁎^	[18, 44]
54	Eriodictyol	287	153, 163 179	24.6/2.7E^5^/ -2,41	24.5/1.7E^5^/ -2.39	24.4/1.0E^5^/ -2.20	Fl	[42]
55	Hesperetin	301	153, 163, 179	24.8/6.5E^4^/ 0.103	24.7/1.2 E^5^/ 0.101	24.8/3.9E^4^/ 0.10	Fl	[42]
56	Apigenin	269	-	24.9/8.4E^5^/ 2.92	24.8/1.1E^5^/ 2.84	24.9/1.5E^5^/ 2.92	L	[42]
57	Naringenin	271	152	-	26.5/2.0E^5^/ -0.39	26.4/1.0E^5^ -0.38	L	[42, 66]
58	Procyanidin tetramer-hexoside	1315	-	-	-	26.9/1.0E^5^/ 1.36	F	[75]
59	Procyanidin C2	865	713, 576, 425, 289	26.9/5.1E^4^/ 2.78	26.8/1.0E^5^ 2.72	26.8/1.4E^5^ 2.70	F, L, Fl	[75, 76]
60	Procyanidin pentamer-hexoside	1605	-	-	28.8/7.1E^4^/ 1.83	28.8/1.0E^5^/ 1.80	F	[75]
61	Procyanidin tetramers	1154	-	28.8/1.6E^5^/ -0.32	28.9/4.4E^4^/ -0.38	28.8/1.2E^5^/ -0.32	F, L, Fl	[75, 76]
62	B-type procyanidin pentamers	1442	1156	29.1/8.5E^4^ 3.24	29.3/4.0E^4^ 3.19	29.0/9.9E^4^ 3.19	F, L, Fl	[75, 76]
63	B-type procyanidin hexamers	1731	1154, 1156, 1444	29.4/1.2E^5^/ 1.42	29.8/3.3E^4^ 1.45	29.5/3.9E^5^/ 1.45	F, L, Fl	[75, 76]

- The MZmine analysis software program, version 2.3, was used to process the data. ^a^ Reported parts from other hawthorn species (*Crataegus* spp.) based on the previous literature sources; fruit (F), leaf (L), flower (Fl), seed (S), and root (R). ^⁎^ Identified phenolic compounds in different parts of *C. pentagyna* in previous works

Below is an explanation of the HPLC-ESI-MS/MS identification of phenolics in fruit, leaf, and root, as well as the comparison of literature.

#### Flavonoids

Flavonoids containing two benzene rings and one oxygenated ring were the most abundant phenols found in hawthorn in this study. According to Table 2, the aerial parts and roots of *C. pentagyna* contain 63 phenolic compounds. In mass spectrometry, all O-glycosides, including glucose or galactose (162 Daltons), rhamnose (146 Daltons), pentose—xylose or arabinose (132 Daltons), and disaccharide structures—rutinose or neohesperidose, lost their sugar moiety (308 Daltons). Due to cross-ring cleavages of sugar residues, C-glycosides exhibited fragments at m/z [M-H-18]^−−^, [M-H-60]^−^, [M-H-90]^−^, [M-H-120]^−^, [M-H-180]^−^, and [M-H-210]^−^ for pentosyl residues and [M-H-74]^−^ and [M-H-104]^−^ for deoxyhexosyl residues [41].

#### Identification of luteolin derivatives

Compounds **50**, **40**, and **41** were identified as luteolin 7-*O*-glucoside, luteolin-7-*O*-glucuronide, and luteolin, respectively. Previous studies have identified compound 41 in *C. microphylla* leaves [42]; compound 50 from fruits, leaves, and flowers of *C. microphylla* [12, 17, 18, 42]; and compound 40 from leaves of *C. macrocarpa* [43].

#### Identification of orientin derivatives

Compounds **36** and **37** were characterized as orientin or isoorientin; and compounds **22** and and **23** as orientin or isoorientin glycoside derivatives (orientin-2″-*O*-rhamnoside and isoorientin-2″-*O*-rhamnoside. Previous studies have identified compound orientin from leaves and flowers of *C. monogyna* and *C. pentagyna*; compound isoorientin from flowers of *C. monogyna* and *C. pentagyna*; compound orientin-2″-*O*-rhamnoside from leaves and flowers of *C. pentagyna*; and compound isoorientin-2″-*O*-rhamnoside from flowers of *C. pentagyna* [12, 17].

#### Identification of quercetin derivatives

Compounds **48** was identified as quercetin aglycone. Compounds **31, 33** and **32** were identified as quercetin-*O-*glycoside derivatives (quercetin 3-*O*-glucoside (isoquercitrin), quercetin 4′-*O*-glucoside (spiraeoside) or quercetin 3-*O*-galactoside (hyperoside); and compounds **18, 21, 20** and **19** as quercetin-3-O-rutinoside, quercetin-3-*O*-rhamnoside, quercetin 7,4′-dimethyl ether-3-*O*-rutinoside and quercetin-3-*O-*(6″ galloyl) glucoside, respectively. Previous studies have identified compound 48 from fruits and leaves of *C. pentagyna*, *C. monogyna*, and *C. oxyacantha*, fruits of *C. pinnatifid*a, *C. germanica*, *C. cuneata*, and *C. brettschneideri*, flowers and leaves of *C. microphylla*, leaves of *C. scabrifolia* and *C. pinnatifid*, and flowers of *C. azarolus* [18, 19, 42, 44, 45]; isoquercitrin from leaves of *C. pentagyna*, flowers of *C. monogyna*, *C. macrocarpa*, *C. rhipidophylla*, *C. laevigata*, and *C. azarolus*, and fruits of *C. scabrifolia* [12, 15, 18, 43, 46]; spiraeoside from fruits and flowers of *C. monogyna* and *C. azarolus* [1, 47–49]; hyperoside from fruits, leaves, and flowers of *C. pentagyna*, seeds and fruits of *C. microphylla*, *C. macrocarpa*, and *C. oxyacantha*, and leaves of *C. pinnatifid*a [12, 15, 18, 42, 43, 50–54]; compound 18 from leaves of *C. scabrifolia* [55]; compound 21 from leaves and flowers of *C. pentagyna* [18]; and compound 20 from fruits of *C. monogyna* [56]; and compound 19 from leaves of *C. monogyna* [56].

#### Identification of kaempferol derivatives

Compounds **53**, **50** and **38** were assigned as kaempferol aglycone, kaempferol-3-*O-*rutinoside and 8-methoxykaempferol (sexangularetin), respectively. Previous studies have identified compound 53 from fruits and flowers of *C. pentagyna* [18, 44]; compound 50 from fruits and leaves of *C. pentagyna* [18]; and compound 38 from flowers of *C. maximowiczii* [51].

#### Identification of apigenin derivatives

Compounds **56** and **42** were identified as apigenin aglycone and apigenin 8-*C-*glucoside (vitexin). Also, compounds **24**, **25**, **28** were detected as vitexin-*O*-rhamnoside derivatives (vitexin 2″-*O*-rhamnoside, isovitexin 2″-*O*-rhamnoside or vitexin-4′- *O*-rhamnoside); and compounds **29** and **26** as vitexin-*O*- glucoside (vitexin 4′-*O*-glucoside or vitexin-2″-*O*-glucoside), respectively. Previous studies have identified compound 56 from leaves of *C. microphylla* [42]; compound 42 from fruits, leaves, and flowers of *C. pentagyna*, *C. monogyna*, *C. microphylla*, and *C. macrocarpa*, and leaves *C. pinnatifid*a [12, 15, 17, 42, 43, 51, 57]; vitexin-2″-*O*-rhamnoside from leaves and flowers of *C. pentagyna*, fruits, leaves, and flowers of *C. monogyna* and *C. pinnatifid*a, leaves of *C. microphylla*, *C. aronia*, *C. pseudoheterophylla*, *C. scabrifolia*, and *C. cuneata*, flowers of *C. macrocarpa*, *C. rhipidophylla*, and *C. laevigata*, and fruits and leaves of *C. pinnatifid*a [48, 58–65]; vitexin-4′-*O*-rhamnoside from leaves of *C. oxyacantha* and leaves and flowers of *C. microphylla* [42, 50, 64, 65]; vitexin-4′-*O*-glucoside from leaves of *C. scabrifolia* and *C. cuneata*, and fruits and leaves of *C. pinnatifid*a [51, 64–66]; and vitexin-2″-*O*-glucoside from leaves of *C. pinnatifid*a [58, 60].

#### Identification of eriodictyol derivatives

Compounds **54, 55** and **39** were assigned as eriodictyol aglycon, hesperetin and eriodictyol-7-glucuronide, respectively. Previous studies have identified compounds **54** and **55** from flowers of *C. microphylla* [42] and compound **39** from leaves and flowers of *C. macrocarpa* [43].

#### Identification of naringenin derivatives

Compounds **57, 27, 43** and **44** were suggested to be naringenin aglycon, naringenin 7-*O*-neohesperidoside (naringin), naringenin-6-*C-*glucoside and naringenin 7-*O*-glucoside, respectively. Previous studies have identified compound 57 from leaves of *C. microphylla* [42, 66]; compound 27 from leaves of *C. oxyacantha* [67]; and compounds 43 and 44 from leaves of *C. monogyna* and *C. laevigata* [68].

#### Identification of catechins, proanthocyanidins, and their derivatives

There are two major categories of procyanidins (A-type and B-type). (Epi)catechin units linked through C4 to C8 or C4 to C6 are called B-type procyanidins, while those with an additional bond (C2-O-C7) are called A-type. In the negative ion mode, there are three characteristic fragmentation routes, including retro Diels–Alder (RDA) [M-152-H]^−^, heterocyclic ring fission (HRF) [M-125-H]^−^, and quinine methide (QM) reaction (cleavage of the interflavan bond), with [M-289-H]^−^ or [M-287-H]^−^ as fragment ions of procyanidins [69]. Due to the degree of polymerization [70], procyanidins may exist as several isomers with the same molecular weight and mass spectrometry. In the present study, samples of *C. pentagyna* contained B-types and their derivatives (Table 2).

Compounds **49**, **51** were detected as catechin or epicatechin monomers. In addition, compounds **35**, **34, 59, 61**, **62**, **63**, **45, 58** and **60** were identified as epicatechin gallate, epigallocatechin gallate, B-type trimer (procyanidin C2), B-type procyanidin tetramer, B-type pentamer, B-type procyanidin hexamer, procyanidin dimer hexosides (procyanidin B3 7-glucoside), procyanidin tetramer glycoside and procyanidin pentamer glycoside, respectively. Previous studies have identified catechin from fruits and leaves of *C. pubescens* [66, 71]; epicatechin from fruits, leaves, and flowers of *C. pentagyna*, callus of *C. monogyna*, fruits and leaves of *C. oxyacantha*, and fruits of *C. pubescens* [1, 18, 19, 72, 73]; compound 59 from fruits and leaves of *C. oxyacantha* [49, 65]; compound 61 from fruits of *C. orientalis* [74] and compounds 45, 58 and 60–63 from fruits of *C. pinnatifid*a [75, 76].

#### Identification of myricetin derivatives

Compound **16**, **30**, **46** and **47** were identified as myricetin-3-*O*-(6″-galloyl) galactoside, myricetin-3-*O*-galactose, myricetin and 5-*O*-methylmyricetin, respectively. Previous studies have identified these compounds from fruits of *C. monogyna* [56].

#### Phenolic acids

Free and conjugated phenolic acids are classified into two groups: hydroxybenzoic acids and hydroxycinnamic acids. Increasing interest in the profile of phenolic acids is due to their possible health benefits and antioxidant activity. In mass spectrometry, phenolic acids and their glycoside derivatives are distinguished by the loss of an ion at *m*/*z* -162 Da (glucose or galactose), followed by the loss of ions at *m*/*z* -18 Da (hydroxyl), -15 Da (methyl), and − 44 Da (carbon dioxide). In this study, a total of 16 phenolic acids were identified.

#### Identification of hydroxybenzoic acids and their glycosidic derivatives

Compound **10** and **12** were identified as protocatechuic acid and salicylic acid. Also, compounds **13** and **14** can be tentatively assigned as hydroxybenzoic acid derivatives. Previous studies have identified compound 12 from fruits of *C. monogyna*; compound 10 from fruits of *C. germanica* and fruits, leaves and flowers of *C. pentagyna* [19, 77]; 3-hydroxybenzoic acid and 4-hydroxybenzoic acid from fruits of *C. germanica* [45, 56, 68]; and hydroxybenzoic acid, hydroxybenzoic acid hexoside from fruits, leaves and flowers of *C. pentagyna* [19].

#### Identification of hydroxycinnamic acids and their glycosidic derivatives

Compounds **17**, **15**, **11**, **9**, **2** and **1** were determined as coumaric acid, ferulic acid, caffeic acid, sinapic acid and sinapic acid-*O*-hexoside and chlorogenic acid glycoside derivative, respectively. Also, compounds **6**, **3** and **4** can be tentatively identified as caffeoylquinic acid derivatives (chlorogenic acid, cryptochlorogenic acid or cryptochlorogenic acid); and compounds **7**, **8**, and **5** as coumaroylquinic acid derivatives (3-*O-*p- coumaroylquinic acid, 4-*O-*p- coumaroylquinic acid or 5-*O-*p- coumaroylquinic acid). Previous studies have identified compound 17 from fruits, leaves, and flowers of *C. pinnatifid*a [18, 78]; compound 15 from fruits, leaves, and flowers of *C. pinnatifid*a and *C. monogyna*, fruits and leaves of *C. scabrifolia* and *C. pinnatifid*a, leaves of *C. cuneata*, fruits of *C. brettschneideri*, leaves and flowers of *C. laevigata*, and flowers of *C. azarolus* [12, 45–47, 57, 77]; compound 11 from fruits, leaves, and flowers of *C. pinnatifid*a and *C. pentagyna*, fruits and leaves of *C. oxyacantha*, and fruits of *C. germanica* [18, 19, 45, 71]; compound 9 from fruits and flowers of *C. monogyna* and fruits of *C. germanica* [45]; compound 2 from fruits and flowers of *C. monogyna* and fruits of *C. germanica* [45]; chlorogenic acid from fruits, leaves, and flowers of *C. pinnatifid*a and *C. pentagyna*, callus of *C. monogyna*, leaves of *C. pinnatifid*a, and fruits of *C. germanica* and *C. pubescens* [15, 18, 19, 45, 51]; cryptochlorogenic acid and neochlorogenic acid from fruits, leaves, and flowers of *C. pinnatifid*a and *C. pentagyna* [12, 18, 19, 33, 79]; 3-*O*-p-coumaroylquinic acid and 5-*O*-p-coumaroylquinic acid from fruits, leaves, and flowers of *C. monogyna* and *C. pentagyna* [19, 68]; 4-*O*-p-coumaroylquinic acid from fruits of *C. pentagyna* [18, 68] and 4-*O*-(3′-*O*-glucopyranosyl)-caffeoyl quinic acid from leaves and fruits of *C. monogyna* and *C. laevigata* [68].

Comparison between phenolic compounds in different parts of *C. pentagyna* based on ion intensity.

The phenolics and their bioactivity are influenced by agronomic, climatic, genomic, processing, and harvesting factors [18]. The relative intensity (E^n^) values indicated that apigenin (8.4E^5^), one of the caffeoylquinic acid derivatives (8.2E^5^), 4-*O*-(3′-*O*-glucopyranosyl)-caffeoyl quinic acid (7.2E^5^), and naringenin-6-*C-*glucoside (3.9E^5^) are the major phenolic compounds in fruit; salicylic acid (3.0E^6^), naringenin-6-*C-*glucoside (5.3E^5^), naringin (3.7E^5^), and one of the caffeoylquinic acid derivatives (4.4E^5^) in leaf; and naringenin-7-*O*-neohesperidoside (6.8E^5^), isovitexin-2″-*O*-rhamnoside (5.7E^5^), 4-*O*-(3′-*O*-glucopyranosyl)-caffeoyl quinic acid (4.8E^5^), and B-type procyanidin hexamers (3.9E^5^) in root. Previous studies on *C. pentagyna* indicated that hyperoside, isoquercetin and chlorogenic acid were other main phenolics in the fruit extracts; isoorientin, isoquercitrin, 8-methoxykaempferol-3-*O*-glucoside, (-)-epicatechin, neochlorogenic and chlorogenic acid, and procyanidin B2 in leaf and flower; and gallic acid and chlorogenic acid in pulp and peel [15, 17, 18]. LC–MS/MS indicated that fruit, leaf, and root of *C. pentagyna* have a similar profile. Meanwhile, orientin, isoorientin, isoorientin-2″-*O*-rhamnoside, quercetin-4′-*O*-glucoside, quercetin-3-*O*-galactoside, kaempferol-3-*O*-rutinoside, vitexin-4′-*O*-glucoside, vitexin-2″-*O*-glucoside, one of the hydroxybenzoic acid derivatives, protocatechuic acid, ferulic acid, caffeic acid, and one of the caffeoylquinic acid derivatives have been observed only in fruit; vitexin-4′-rhamnoside, eriodictyol-7-glucuronide, procyanidin B3 7-glucoside, and coumaroylquinic acid derivative only in leaf; and one of the caffeoylquinic acid derivatives only in root.

### GC-MS analysis of the petroleum ether extracts

**Fig. 2:**
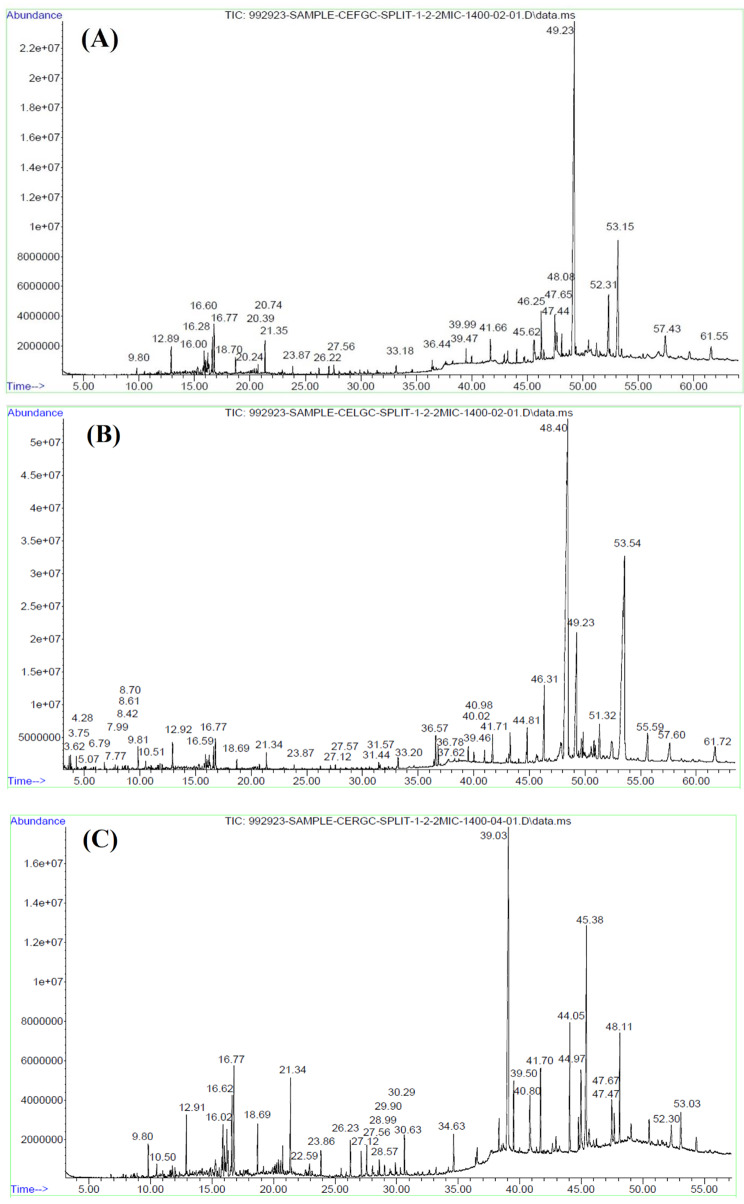
Gas ion chromatogram of *C. pentagyna* extracts: (**A**) fruit; (**B**) leaf; (**C**) root

Currently, there are no reports on GC-MS analysis of *C. pentagyna* extracts. In this study, for the first time, GC-MS was used to investigate the chemical composition of various petroleum ether extracts (Fig. 2). As shown in Table 3, the analysis of extracts identified 28, 42, and 25 chemical compounds belonging to different chemical families, representing 91.13%, 95.71%, and 89.67%, respectively, of the relative area in the fruit, leaf, and root extracts. The fruit extract consists of hydrocarbons (63.80%), fatty acids and their derivatives (11.77%), steroids (9.54%), and terpenes (2.051%), with nonacosane (50.74%), tetratetracontane (7.22%) and γ-sitosterol (6.11%). Hydrocarbons (49.20%), fatty acids and their derivatives (13.85%), steroids (13.04%) and terpenes (10.95%) and predominated in the leaf extract, with nonacosane (26.29%), squalene (10.29%), γ-sitosterol (7.30%) and oleyl palmitoleate (6.53%). In addition, the root extract contained hydrocarbons (53.96%), terpenes (13.06%), steroids (6.83%) and fatty acids and their derivatives (6.70%) with squalene (10.15%), bis(2-ethylhexyl) adipate (9.65%) and tributyl acetylcitrate (6.42%). Several studies have documented the antimicrobial and antioxidant effects of nonpolar extracts [80–82].

**Table 3:** Chemical composition of petroleum ether extracts from *C. pentagyna* fruit, leaf, and root analyzed by GC-MS.

Compounds	Molecular Formula	Classification	RT (min)			Percentage %		
			Fruit	Leaf	Root	Fruit	Leaf	Root
2-Methylheptane	C_8_H_18_	Alkane hydrocarbon	-	3.628	-	-	0.779%	-
3-Methylheptane	C_8_H_18_	Alkane hydrocarbon	-	3.758	-	-	0.824 %	-
Octane	C_8_H_18_	Alkane hydrocarbon	-	4.283	-	-	0.662 %	-
2,6-Dimethylheptane	C_9_H_20_	Alkane hydrocarbon	-	4.915	-	-	0.128 %	-
1,2-Dimethylcyclohexane	C_8_H_16_	Cyclo alkane	-	4.953	-	-	0.102 %	-
2,5-Dimethylheptane	C_9_H_20_	Alkane hydrocarbon	-	5.074	-	-	0.280 %	-
1,1,3-Trimethylcyclohexane	C_9_H_18_	Cyclo alkane	-	5.141	-	-	0.160 %	-
2,3,5-Trimethylhexane	C_9_H_20_	Alkane hydrocarbon	-	5.600	-	-	0.091 %	-
2-Methyloctane	C_9_H_20_	Alkane hydrocarbon	-	5.819	-	-	0.218 %	-
2,5-Dimethylheptane	C_9_H_20_	Alkane hydrocarbon	-	6.015	-	-	0.179 %	-
Nonane	C_9_H_20_	Alkane hydrocarbon	-	6.790	-	-	0.446 %	-
2,6-Dimethyloctane	C_10_H_22_	Alkane hydrocarbon	-	7.776	-	-	0.353 %	-
3-Ethyl-2-methylheptane	C_10_H_22_	Alkane hydrocarbon	-	7.994	-	-	0.309 %	-
3,7,11-Trimethyl-1-dodecanol	C_15_H_32_O	Alkane hydrocarbon	-	8.423	-	-	0.292 %	-
4-Methylnonane	C_10_H_22_	Alkane hydrocarbon	-	8.619	-	-	0.380 %	-
2-Methylnonane	C_10_H_22_	Alkane hydrocarbon	-	8.709	-	-	0.240 %	-
Decane	C_10_H_22_	Alkane hydrocarbon	9.803	9.816	9.809	0.290%	1.574 %	1.665%
4-Methyldecane	C_11_H_24_	Alkane hydrocarbon	-	10.518	10.509	-	0.675 %	0.751%
Undecane	C_11_H_24_	Alkane hydrocarbon	12.890	12.925	12.911	1.189%	2.341 %	3.310%
2,6-Dimethyldecalin	C_12_H_22_	Polycyclic hydrocarbon	16.006	-	16.020	0.873%	-	2.313%
Decahydro-1,6-dimethylnaphthalene	C_12_H_22_	Polycyclic hydrocarbon	16.285	-	-	0.609%	-	-
2,6-Dimethyldecalin	C_12_H_22_	Polycyclic hydrocarbon	16.608	16.599	16.622	1.842%	2.170 %	5.222%
1,5-Dimethyldecahydronaphthalene	C_12_H_22_	Polycyclic hydrocarbon	16.774	16.779	16.787	3.163%	3.011 %	9.014%
Tridecane	C_13_H_28_	Alkane hydrocarbon	18.701	18.699	18.707	0.845%	0.756 %	3.218%
Geranyl isovalerate	C_15_H_26_O_2_	Fatty ester	20.244	-	-	0.286%	-	-
2,6,10-Trimethyltetradecane	C_17_H_36_	Isoprenoid hydrocarbon	20.395	-	-	0.4312%	-	-
Farnesan	C_15_H_32_	Sesquiterpene	20.741	-	20.732	0.479%	-	1.823%
Tetradecane	C_14_H_30_	Alkane hydrocarbon	21.351	21.349	21.357	1.565%	1.196 %	6.375%
2-Methyl-1-hexadecanol	C_17_H_36_O	Fatty alcohol	-	-	22.591	-	-	0.888%
Pentadecane	C_15_H_32_	Alkane hydrocarbon	23.873	23.863	23.871	0.4280%	0.346 %	1.482%
Hexadecane	C_16_H_34_	Alkane hydrocarbon	26.229	-	26.235	0.298 %	-	2.207%
8-Amino-6-methoxy-4-methyl-5-[n-nonoxy]-quinoline	C_10_H_10_N_2_O	Heterocyclic aromatic derivatives	-	27.122	-	-	0.277 %	-
Benzene, 1,3,5-tris(1-methylpropyl)-	C_18_H_30_	Benzene derivatives	27.569	27.574	27.567	0.493%	0.462 %	2.316%
11-Eicosenoic acid	C_20_H_38_O_2_	Fatty Acid	-	-	28.576	-	-	1.256%
3α-Methyl-24-noroleana-4(23),12-diene	C_30_H_48_	Triterpene	-	-	28.997	-	-	1.089%
Naphthalene, 1,2,3,4-tetrahydro-1-isopropyl-1,2,4,4,7-pentamethyl-	C_18_H_28_	Polycyclic aromatic hydrocarbon	-	-	29.908	-	-	0.899%
Octadecane	C_18_H_38_	Alkane hydrocarbon	-	-	30.638	-	-	2.385%
3,7,11,15-Tetramethyl-2-hexadecen-1-ol	C_20_H_40_O	Diterpene	-	31.443	-	-	0.656 %	-
6,10,14-Trimethylpentadecan-2-one	C_18_H_36_O	Sesquiterpenoid	-	31.586	-	-	0.415 %	-
7,9-Di-tert-butyl-1-oxaspiro(4,5)deca-6,9-diene-2,8-dione	C_17_H_24_O_3_	Lactone	33.185	-	-	0.814%	-	-
Methyl palmitate	C_17_H_34_O_2_	Fatty acid ester	-	33.205	-	-	1.581 %	-
Eicosane	C_20_H_42_	Alkane hydrocarbon	-	-	34.636	-	-	2.643%
Methyl linolelaidate	C_19_H_34_O_2_	Fatty acid ester	36.442	-	-	0.72 %	-	-
Methyl linolenate	C_19_H_32_O_2_	Fatty acid ester	-	36.570	-	-	2.904 %	-
Oleic Acid	C_18_H_34_O_2_	Fatty acid	-	37.623	-	-	2.834 %	-
Tributyl acetylcitrate	C_20_H_34_O_8_	Citrates (tricarboxylic acid)	39.470	-	39.506	0.924%	-	6.424%
Oleic acid, 3-(octadecyloxy)propyl ester	C_39_H_76_O_3_	Fatty acid ester	39.997	-	-	0.628%	-	-
17-Pentatriacontene	C_35_H_70_	Unsaturated hydrocarbon	-	40.025	47.674	-	1.412 %	8.667%
4,8,12,16-Tetramethylheptadecan-4-olide	C_21_H_40_O_2_	Lactone	-	40.981	-	-	0.961 %	-
Dioctyl adipate	C_22_H_42_O_4_	Adipate (dicarboxylic acids)	41.668	41.711	-	1.822%	1.411 %	-
Bis(2-ethylhexyl) adipate	C_22_H_42_O_4_	Adipate (dicarboxylic acids)	-	-	41.704	-	-	9.659%
Diisooctyl phthalate	C_24_H_38_O_4_	Phthalate ester	-	-	44.053	-	-	4.805%
Hexacosane	C_26_H_54_	Alkane hydrocarbon	-	44.812	-	-	2.700 %	-
Docosyl heptanoate	C_29_H_58_O_2_	Fatty esters	45.628	-	-	2.501%	-	-
Heptacosane	C_27_H_56_	Alkane hydrocarbon	46.253	46.310	-	3.065%	4.490 %	-
Erucamide	C_22_H_43_NO	Fatty amide	47.442	-	47.470	3.591%	-	4.583%
Oleic acid, 3-(octadecyloxy)propyl ester	C_39_H_76_O_3_	Fatty acid ester	47.653	-	-	4.046%	-	-
Lycopersene	C_40_H_66_	Tetraterpene	48.089	-	-	1.561%	-	-
Squalene	C_30_H_50_	Triterpene	-	48.403	48.118	-	10.298 %	10.156%
Nonacosane	C_29_H_60_	Alkane hydrocarbon	49.234	49.239	-	50.747%	26.295 %	-
Oleyl palmitoleate	C_34_H_64_O_2_	Fatty esters	-	51.324	-	**-**	6.537 %	-
Stigmast-5-en-3-ol, oleate	C_29_H_50_O	Steroid	-	-	52.303	**-**	-	6.83%
Tetratetracontane	C_44_H_90_	Alkane hydrocarbon	52.313	-	-	7.223%	-	-
Triacontane-1,30-diol	C_30_H_62_O_2_	Alcohol	-	55.592	-	**-**	6.186 %	-
γ-Sitosterol	C_29_H_50_O	Steroid	57.431	57.602	-	6.117%	7.308 %	-
Pollinasterol	C_29_H_48_O	Steroid	61.557	-	-	3.430%	-	-
β-Sitosterol acetate	C_31_H_52_O_2_	Steroid	-	61.720	-	-	5.740 %	-
**Major Grouped Compounds**						**Fruits**	**Leaves**	**Roots**
Terpenes						2.051%	10.954%	13.06%
Fatty acids, Fatty acid esters, Fatty amids						11.77%	13.85%	6.7%
Steroids						9.54%	13.04%	6.83%
Hydrocarbons						**63.80%**	**49.20%**	**53.96**%
Miscellaneous						3.97%	5.7%	6.99%
**Total Identified%**						91.13%	95.71%	89.67%

#### Antimicrobial activities

Table 4 provides a summary of the antibacterial activities of petroleum ether and hydro-methanolic extracts of *C. pentagyna* fruit, leaf, and root against two pathogenic bacteria (*Staphylococcus aureus* and *Escherichia coli*). All examined extracts inhibited bacterial growth with MIC and MBC values ranging from 0.15 to 5.12 mg/mL and 0.15 to 10.12 mg/mL, respectively. The activity of the petroleum ether extract of leaf was higher than that of the root and fruit extracts (MICs 1.25–5 mg/mL). The presence of terpenes and flavonoids explains the high antibacterial activity in petroleum ether and hydro-methanol extracts, respectively [83]. . few studies have been conducted on the crude extracts of *C. pentagyna* to date. Salmanian et al. [16] examined the antimicrobial activity of seed and pulp extracts against four clinical pathogens. These extracts inhibited bacterial strains, with MICs and MBCs ranging between 2.5 and 40 mg/mL and 5 and > 40 mg/mL, respectively. In the study of Safapour et al. [84], *C. pentagyna* fruit extract demonstrated potent antibacterial activity, especially against Gram-negative bacteria. In a separate study, fruit acetonic extract exhibited the highest antibacterial activity against *Bacillus subtilis* (MBC = 2.5 mg/mL) [85]. These antibacterial effects may be attributable to the flavonoid content of the extracts, consistent with our findings. Terpenoids are known for their antibacterial effect and aromatic qualities. Non-polar constituents are more soluble in non-polar solvents and polar constituents are more soluble in polar solvents, so there was a different antibacterial effect between solvents. Polar and non-polar solvents have high capacity to dissolve active antimicrobial compounds than the medium polar solvent. Terpenoids are fat soluble, so these phytochemicals can be attracted to the petroleum ether solvent as non-polar solvent [86].

**Table 4:** MICs and MBCs (mean ± SD) (mg/mL) of *C. pentagyna* petroleum ether (EP) and hydro-methanol (HM) extracts against pathogenic bacteria (mg/mL)

Plant part	S. aureus				E.coli			
	MIC		MBC		MIC		MBC	
	EP	HM	EP	HM	EP	HM	EP	HM
Fruit	2.52 ± 2.11 ^a^	0.15 ± 1.32 ^b^	5.25 ± 2.14 ^c^	0.62 ± 3.14 ^d^	5.12 ± 1.42 ^c^	0.62 ± 2.94 ^d^	10.12 ± 2.76 ^f^	1.25 ± 3.12 ^g^
Leaf	0.31 ± 1.09 ^a^	0.31 ± 1.45 ^a^	0.62 ± 1.32 ^b^	2.52 ± 3.52 ^c^	0.31 ± 1.15 ^a^	0.15 ± 2.31 ^d^	1.25 ± 2.77 ^e^	0.31 ± 2.34 ^a^
Root	1.25 ± 1.21 ^a^	1.25 ± 3.16 ^a^	5.78 ± 1.38 ^b^	2.52 ± 2.41 ^c^	2.58 ± 1.56 ^c^	2.52 ± 1.11 ^c^	5.56 ± 2.86 ^b^	5.25 ± 3.32 ^d^
Gentamicin	0.00229 ± 1.51		0.03196 ± 1.85		0.01651 ± 1.15		0.12668 ± 2.41	

Means with different superscript lowercase letters within the same row differ significantly (*p* < 0.05)

### Principle component analysis (PCA) and heatmap analysis

**Fig. 3:**
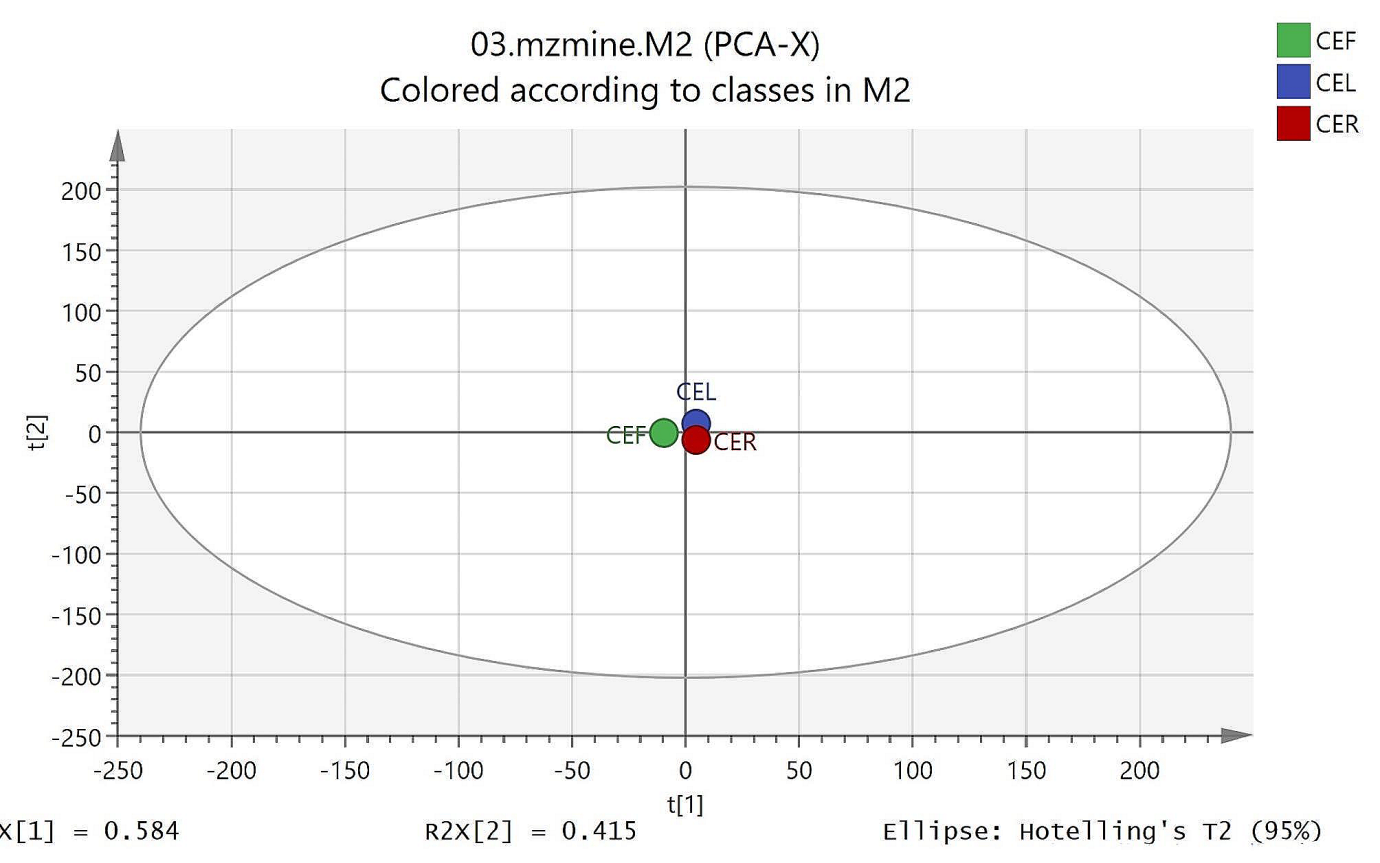
Principal component analysis (PCA) score plot of samples including CEF, CEL and CER

**Fig. 4:**
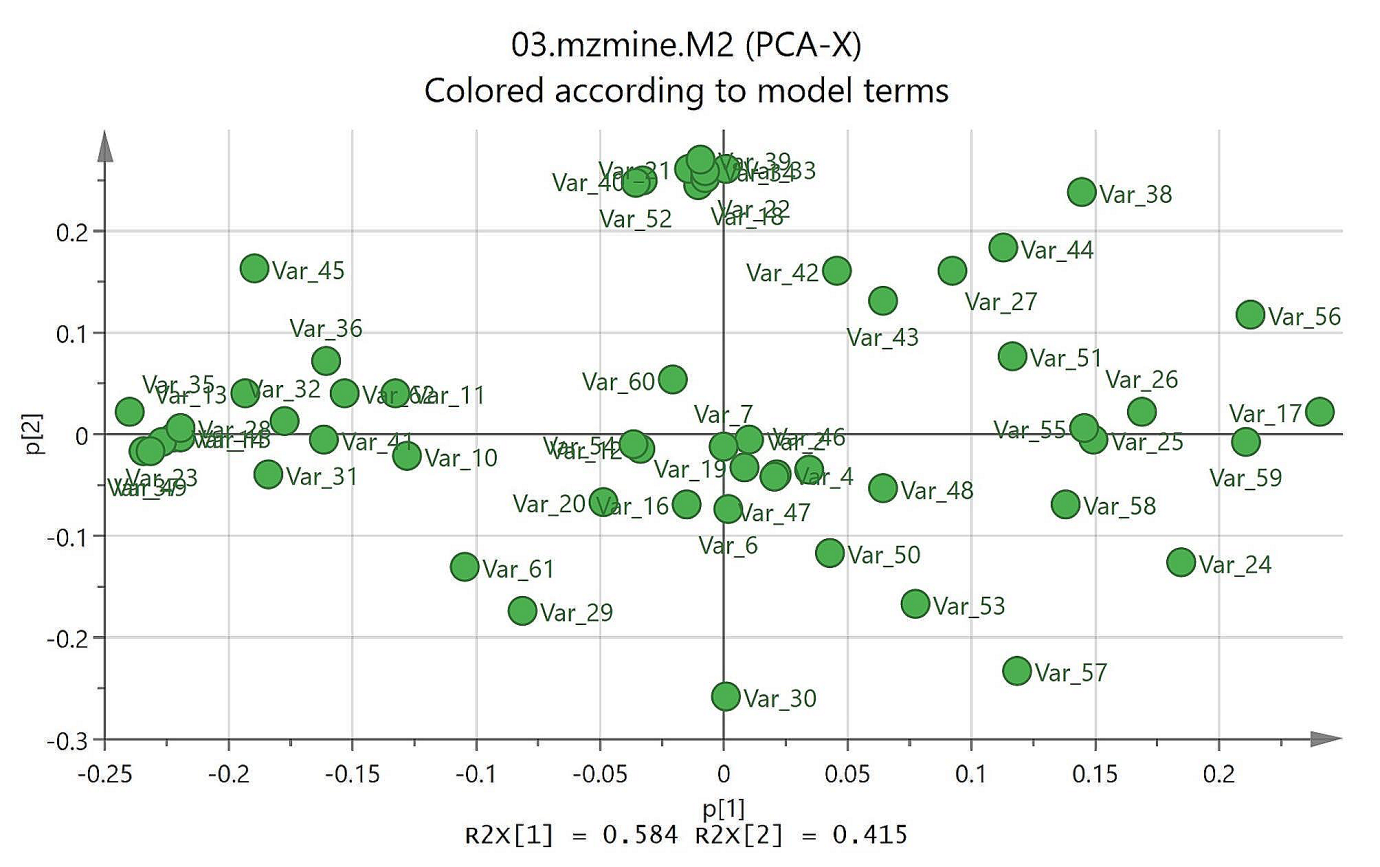
Principal component analysis (PCA) loading plot of samples including CEF, CEL and CER

**Fig. 5:**
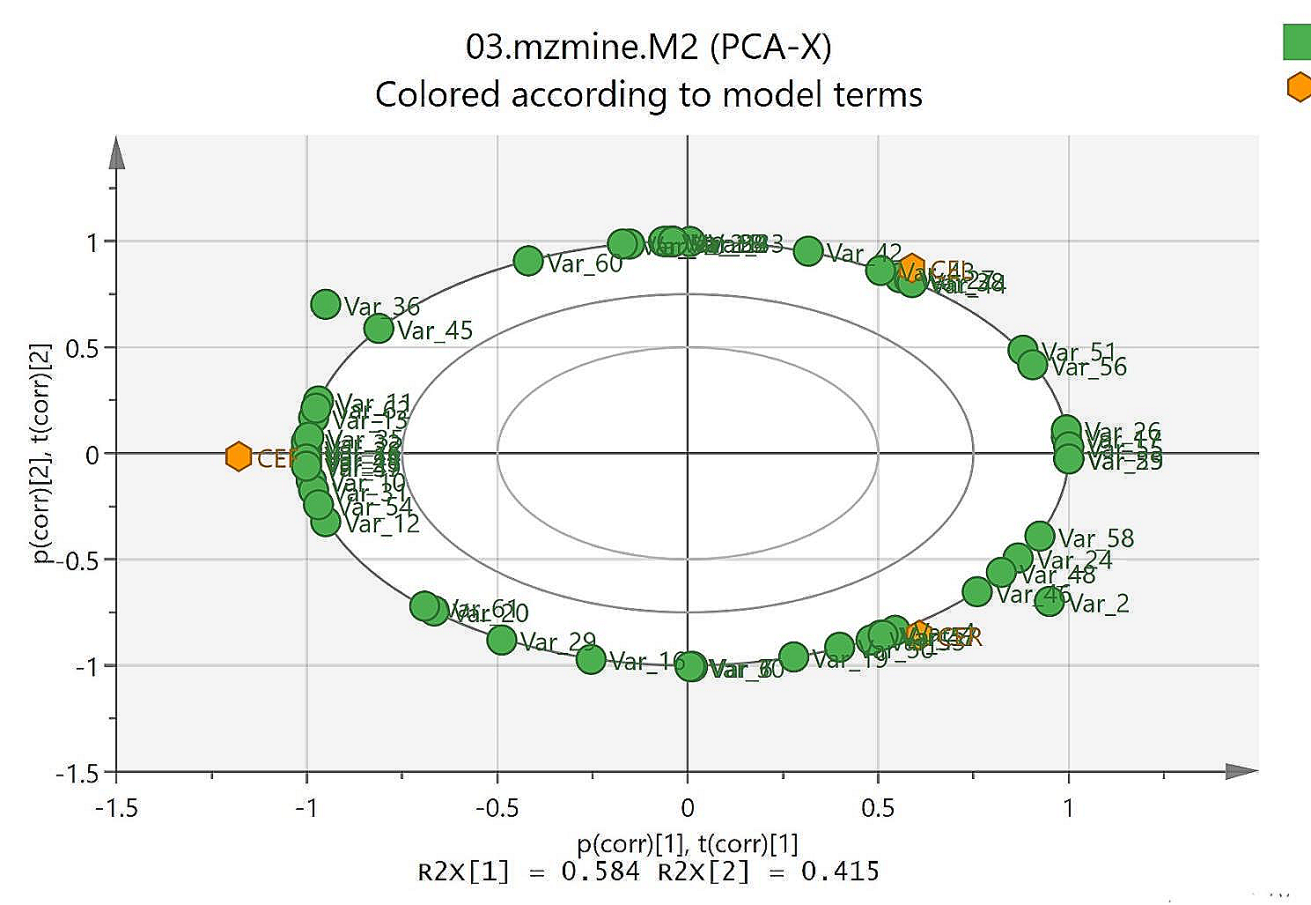
Principal component analysis (PCA) biplot of samples including CEF, CEL and CER

The PCA model as the most common multivariate data analysis is an unsupervised method to reduce the dimensionality of huge multivariate data sets. As shown in Fig. 3 samples (CEF: fruit, CEL: leaf and CER: root) revealed a clear difference in the PCA model, indicating differences in metabolic fingerprints of samples. The resulting values of R2X(cum) and Q2(cum) of 0.96 and 0.98, respectively, indicating a good fitness, discrimination, and predictability of the PCA model. The loading plot (Fig. 4) and bipolot (Fig. 5) are useful for identifying the variable responsible for similarities and differences between samples. The assignment of these variables led to the identification of compounds that are responsible for separation in the score plot. The variables of salicylic acid, luteolin, catechin, eriodictyol, quercetin, hesperetin, 5-*O*-methylmyricetin, apigenin-8-*C*-glucoside, naringenin-6-*C*-glucoside, naringenin-7-*O*-glucoside, chlorogenic acid glycoside, quercetin-3-*O*-(6″ galloyl) glucoside, myricetin-3-*O*-(6″ galloyl) galactoside, quercetin-7,4′-dimethyl ether-3-*O*-rutinoside, procyanidin C2, procyanidin tetramers, B-type procyanidin pentamers and B-type procyanidin hexamers were identified for all the samples; caffeoylquinic acid derivative, quercetin-3-*O*-rutinoside, quercetin-3-*O*-rhamnoside, orientin glycoside, epigallocatechin gallate, epicatechin gallate, luteolin 7-*O*-glucoside, luteolin-7-*O*-glucuronide, myricetin and kaempferol for fruit and leaf; sinapic acid-*O*-hexoside, myricetin-3-*O*-galactose and quercetin-*O*-glycoside derivative for fruir and root; and coumaroylquinic acid derivative, sinapic acid, coumaric acid, vitexin-*O*-rhamnoside derivative, naringenin-7-*O*-neohesperidoside, naringenin and procyanidin pentamer-hexoside for leaf and root. Also, caffeoylquinic acid derivative, coumaroylquinic acid derivative, protocatechuic acid, caffeic acid, hydroxybenzoic acid derivative, ferulic acid, orientin glycoside, vitexin-*O*-glucoside derivative, quercetin-*O*-glycoside derivative, orientin, 8-methoxykaempferol and kaempferol-3-*O*-rutinoside were found only in fruit; coumaroylquinic acid derivative, eriodictyol-7-glucuronide and procyanidin B3 7-glucoside in leaf; and caffeoylquinic acid derivative and procyanidin tetramer-hexoside in root. The heatmap analysis was presented in Fig. S2 to better show differences between metabolites. (Supplementary materials). The metabolites of Table 2 were displayed in the heat map as variables 1–62. The major metabolites based on the relative abundance in heat map included some phenolic compounds including variables 1,3,5, 10–15, 23, 27,31,32, 35–37, 41,45, 49,54, 61 and 62 in fruit sample (CEF); variables 8,9, 17,18, 21,22, 26,27, 32, 33, 38–40, 42–44, 51,52 and 56 in leaf (CEL); and variables 4, 6, 19, 24, 30, 46–48, 50, 53, 57 and 58 in root (CER). As shown in the heat map, both CEF and CEL sample contains several metabolites with significantly higher abundance, whereas, less metabolites with high abundance were found in the sample of CER.

## Conclusions

In this study, the compound profiles in petroleum ether and hydro-methanolic extracts of *C. pentagyna* fruit, leaf, and root tentatively were identified and characterized. In addition, the chemical profile of hawthorn root was discovered for the first time. The fruit hydro-methanolic extract exhibited the highest levels of antioxidant and antibacterial activity, followed by the leaf and root extracts. Antibacterial and antioxidant activities of *C. pentagyna* extracts were attributed to the major phenolics and terpenes detected by HPLC-MS/MS and GC-MS. Using LC-ESI-MS, it was possible to characterize 62 compounds including mainly flavone apigenin, phenolic acid salicylic acid and flavanone naringin in fruit, leaf and root, respectively. Also, bioactive compounds such as alkane nonacosane in fruit and leaf extracts and triterpene squalene in root extract were identified using GC-MS as major components. The results of the PCA and heatmap analysis distinguished metabolite profile differences for fruit, leaf and root samples into well-defined groups. To confirm their potential as phytotherapeutic agents, it is suggested that further research should be conducted, including the purification of the main compounds and the investigation of their biological activities and mechanisms of action.

### Electronic supplementary material

Below is the link to the electronic supplementary material.

Supplementary Material 1

Supplementary Material 2

## Data Availability

The authors declare that the data supporting the findings of this study are available within the paper and its Supplementary Information files. Should any raw data files be needed in another format they are available from the corresponding author upon reasonable request.

## Declarations

All methods were performed in accordance with the relevant guidelines and regulations.

The plant samples used in this study were collected from Farsian, Galikesh, Golestan province, Iran. Identification of this species was confirmed by Dr. Ali Satarian from the Biology department, Science College, Gonbade Kavous university, Gonbad Kavous, Iran. The voucher specimen (803892) has been deposited at Dr. Ali Satarian’s laboratory for reference.

## Abbreviations

LC–ESI-MS/MS: liquid chromatography coupled with electrospray ionization tandem mass spectrometry
GC-MS: gas chromatography coupled with mass spectrometry
EP: ether petroleum
HM: hydro-methanol
TP: total phenol
TF: total flavonoid
TPA: total phenolic acid
TAC: total anthocyanin
RT: retention time
MHB: Muller Hinton broth
BHT: butylate hydroxytoluene
RDA: retro Diels–Alder
DPPH: 1,1-diphenyl-2-picrylhydrazyl
XIC: extracted ion chromatograms
TIC: total ion chromatogram
PCA: principal component analysis
LSD: least significant difference
