# Supplementary material for: Crataegus pentagyna willd. Fruits, leaves and roots: phytochemicals, antioxidant and antimicrobial potentials
Source: BMC Complement Med Ther. 2024 Mar 19;24:126. doi: 10.1186/s12906-024-04430-4 (PMC10949799; doi:10.1186/s12906-024-04430-4)
Supplement: Supplementary file 1 — Supplementary Material 1 [file 12906_2024_4430_MOESM1_ESM.docx]

Table S1. Structures of phenolic compounds identified in *C.pentagyna.*

| **Luteolin derivatives** | | | |
| --- | --- | --- | --- |
|  |  |  |  |
| Luteolin | Luteolin 7-O-glucoside (Cynaroside) | Luteolin-7-*O-*glucuronide | Orientin |
|  |  |  | |
| Isoorientin | Orientin-2''-O-rhamnoside | Isoorientin-2''-O-rhamnoside | |
| **Quercetin derivatives** | | | |
|  |  |  |  |
| Quercetin | Quercetin 3-O-glucoside, isoquercitrin | quercetin 4'-O-glucoside (spiraeoside( | Quercetin 3-O-galactoside, hyperoside |
|  |  |  |  |
| Quercetin-3-O-rutinoside | Quercetin-3-O-rhamnoside | Quercetin 7,4′-dimethyl ether-3-O-rutinoside | quercetin-3-O-(6′′ galloyl) glucoside |
| **Kaempferol derivatives** | | | |
|  |  |  |  |
| Kaempferol | Kaempferol-3-O-rutinoside (Nicotiflorin) | Kaempferol-3-O-glucoside, astragalin | 8-methoxykaempferol, sexangularetin |
|  | | | |
| 8-methoxykaempferol -3-O- glucose | | | |
| **Apigenin derivatives** | | | |
|  |  |  |  |
| Apigenin | Apigenin-8-C-glucoside (vitexin) | Apigenin 6-C-glucoside (isovitexin) | Apigenin-7-O-glucoside |
|  |  |  |  |
| Vitexin-2″-O-rhamnoside | Isovitexin 2″-O-rhamnoside | Vitexin-4'-rhamnoside | Isovitexin-4'-rhamnoside |
|  |  |  | |
| Vitexin-4'-O-glucoside | Vitexin-2″-O-glucoside | Vitexin-4″-O-glucoside | |
| **Eriodictyol derivatives** | | | |
|  |  |  | |
| Eriodictyol | Hesperetin | Eriodictyol-7-glucuronide | |
| **Naringenin derivatives** | | | |
|  |  |  |  |
| Naringenin | Naringenin 7-O-neohesperidoside (naringin) | Naringenin-6-C-glucoside | Naringenin-7-O-glucoside |
| **Catechins, proanthocyanidins** | | | |
|  |  |  |  |
| Catechin | Epicatechin | Epicatechin gallate | Procyanidin B1 or procyanidin B2 |
|  | |  | |
| Procyanidin C2 | | Procyanidin tetramers | |
| **Myricetin derivatives** | | | |
|  |  |  |  |
| Myricetin | Myricetin-3-O-galactose | Myricetin-3-O-rhamnoside, myricitrin | 5-O-methylmyricetin |
|  | |  | |
| Myricetin-3-O- (6” galloyl) galactoside | | Myricetin-3-O- (6” galloyl) galactoside | |
| **Hydroxybenzoic acids** | | | |
|  |  |  |  |
| Salicylic acid | Syringic acid | Salicylic acid O-glucoside | Syringic acid O-glucoside |
|  |  |  |  |
| Gallic acid | 3-hydroxybenzoic acid | 4-hydroxybenzoic acid | Protocatechuic acid |
|  | | | |
| Protocatechuic acid-O-hexoside | | | |
| **Hydroxycinnamic acids** | | | |
|  |  |  |  |
| Coumaric acid | Ferulic acid | Caffeic acid | Sinapic acid |
|  |  |  |  |
| Sinapic acid O-glucoside | 3-O-caffeoylquinic acid (chlorogenic acid) | 4-O-caffeoylquinic acid (cryptochlorogenic acid) | 5-O-caffeoylquinic acid (neochlorogenic acid) |
|  |  |  | |
| 3-O-p-coumaroylquinic acid | 4-O-p-coumaroylquinic acid | 5-O-p-coumaroylquinic acid | |
