# Supplementary material for: Crataegus pentagyna willd. Fruits, leaves and roots: phytochemicals, antioxidant and antimicrobial potentials
Source: BMC Complement Med Ther. 2024 Mar 19;24:126. doi: 10.1186/s12906-024-04430-4 (PMC10949799; doi:10.1186/s12906-024-04430-4)
Supplement: Supplementary file 2 — Supplementary Material 2 [file 12906_2024_4430_MOESM2_ESM.docx]

**(A)**


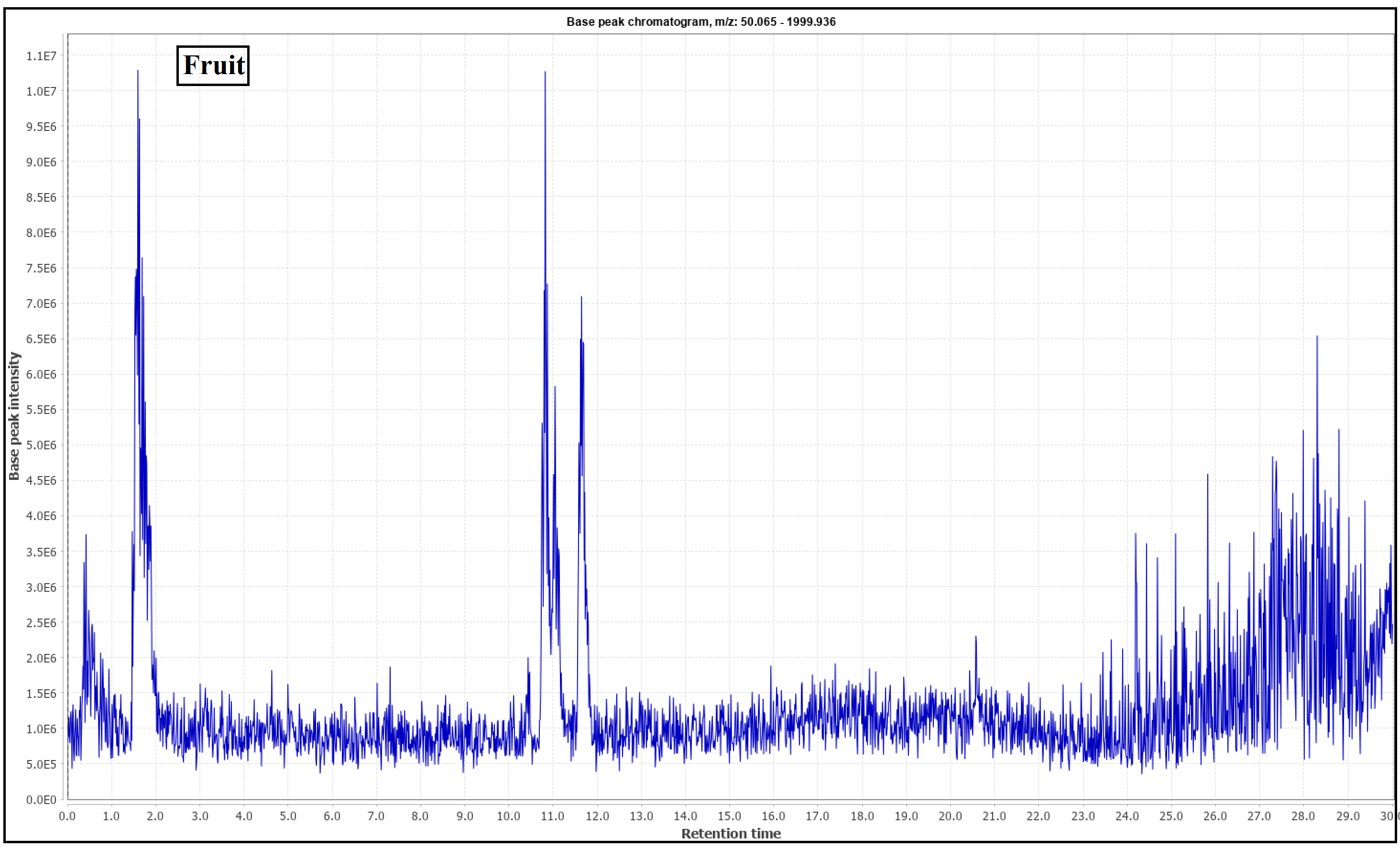


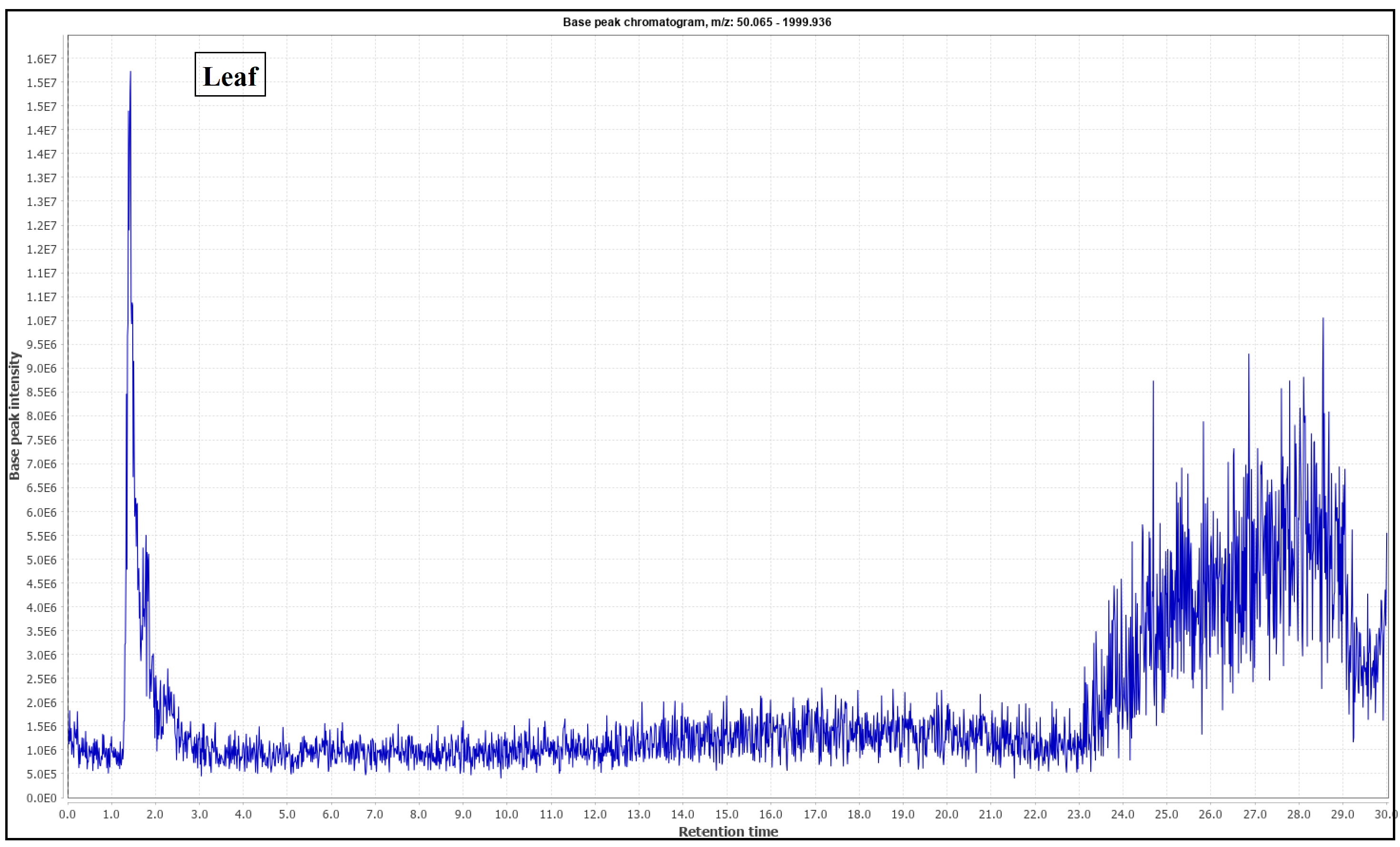


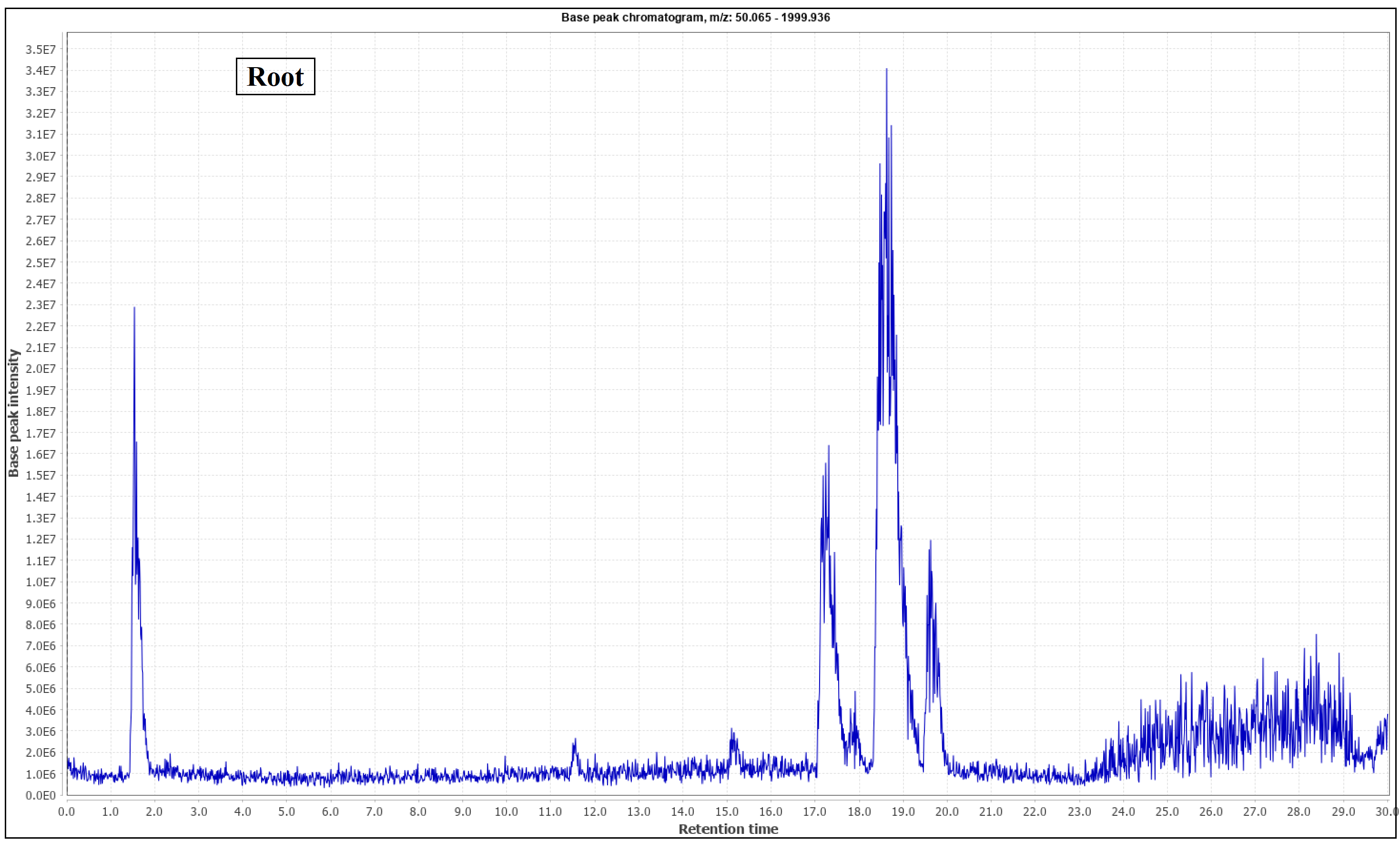


**(B)**


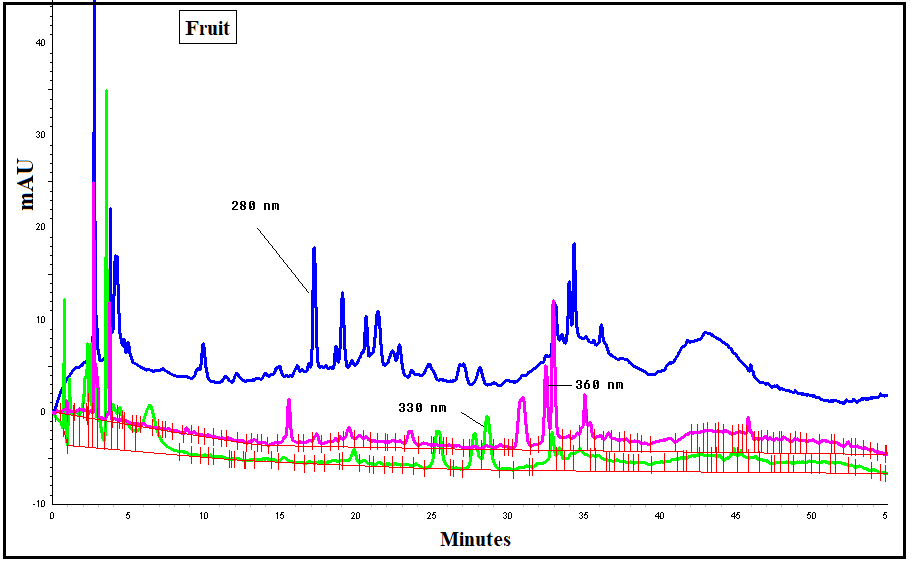


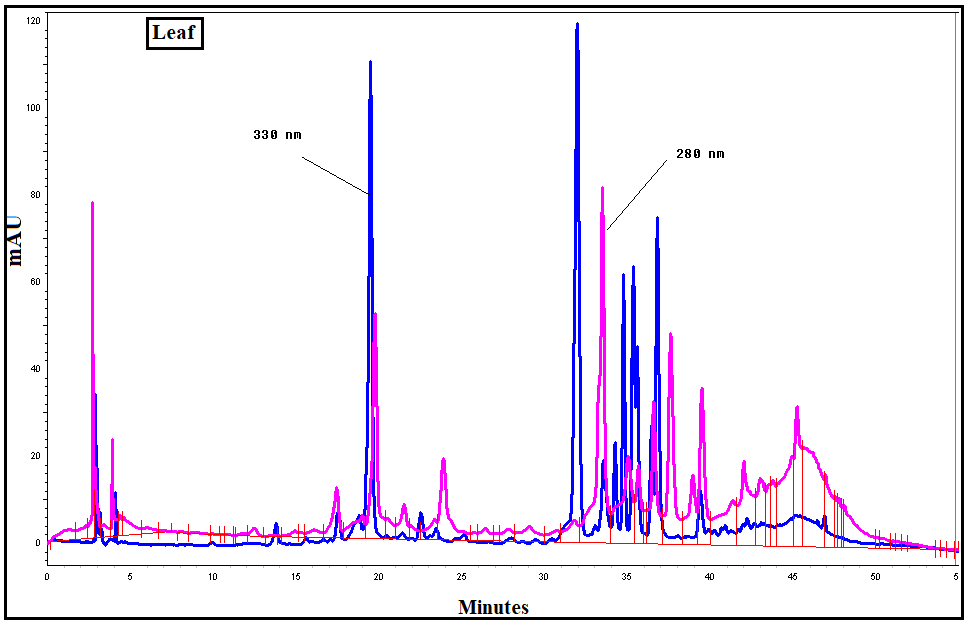


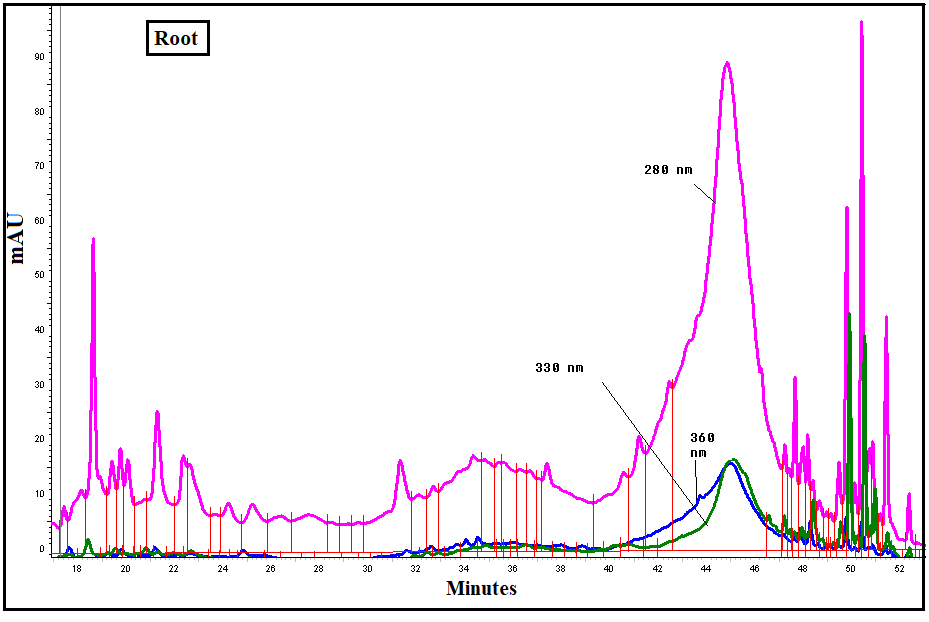


**Fig S1.** (A) LC-MS/MS and (B) LC-PDA chromatograms of hydro-methanolic extracts of the fruit, leaf, and root of *C. pentagyna*.


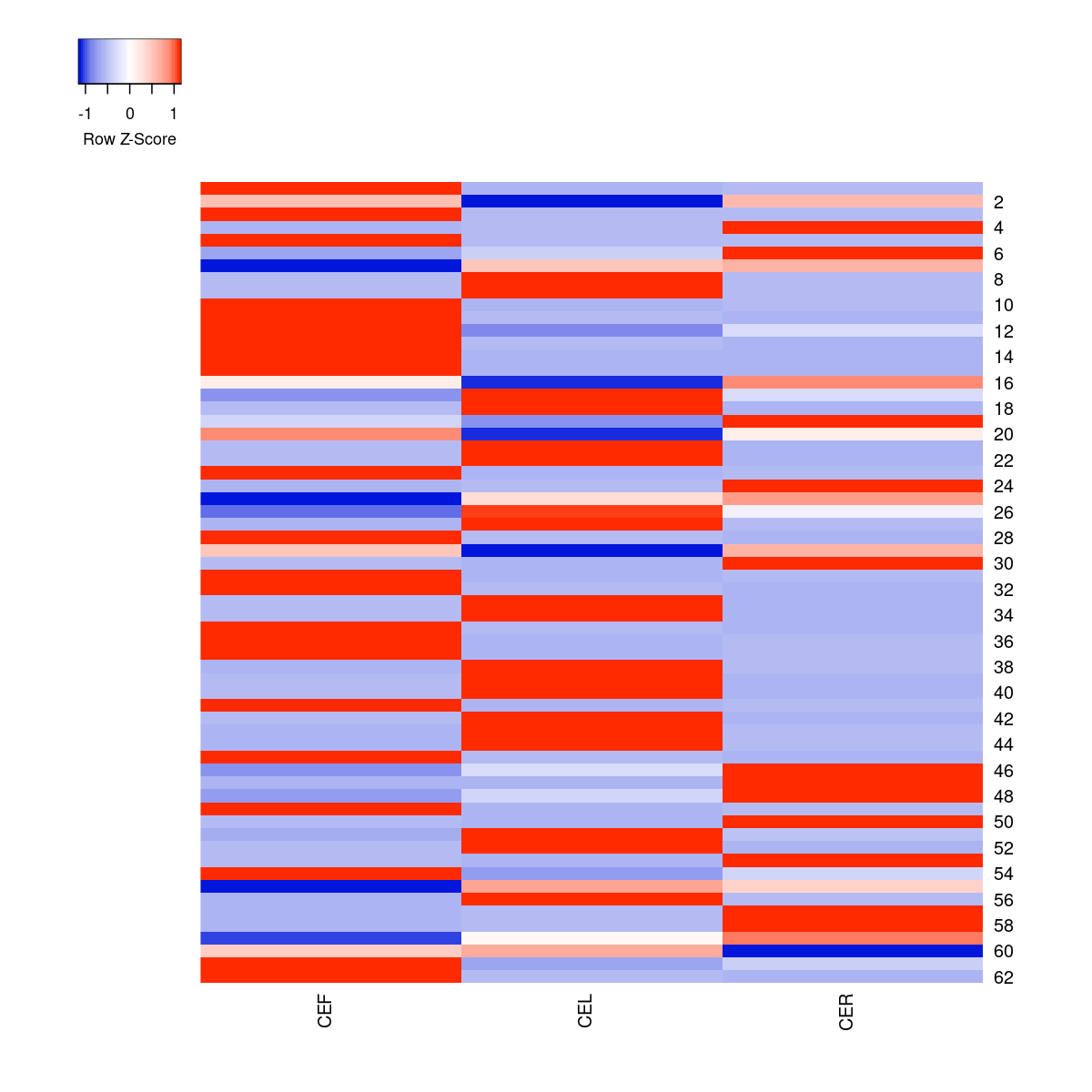


**Fig S2.** Heatmap analysis of identified metabolites in CEF, CEL and CER samples


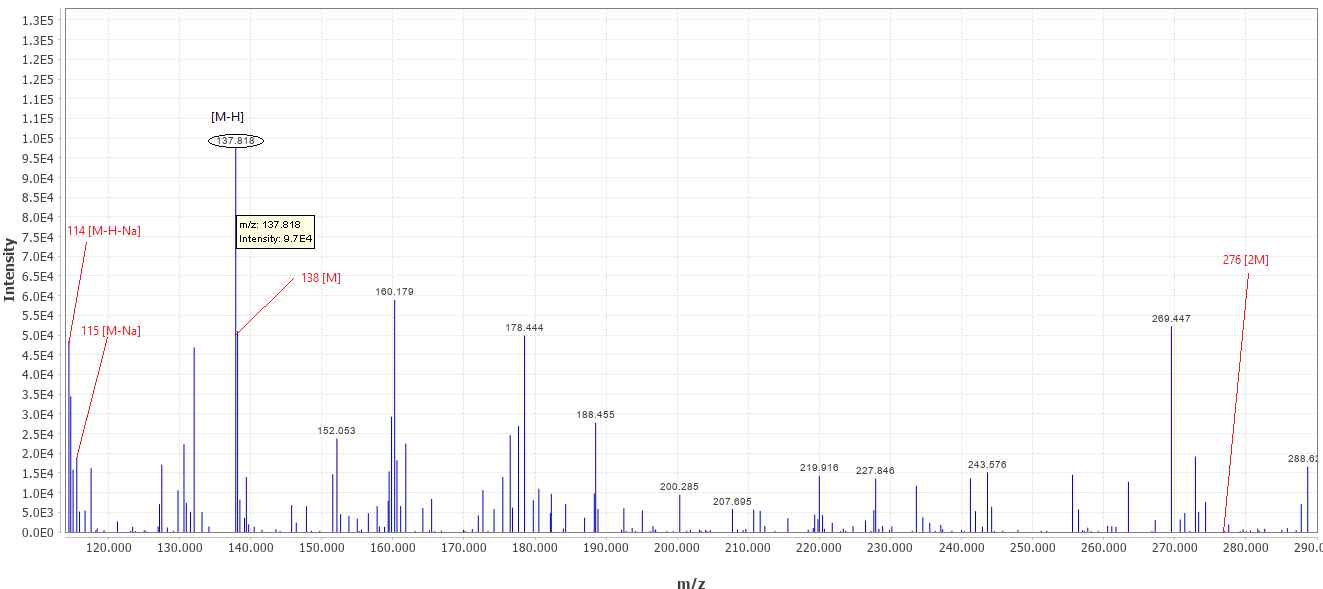


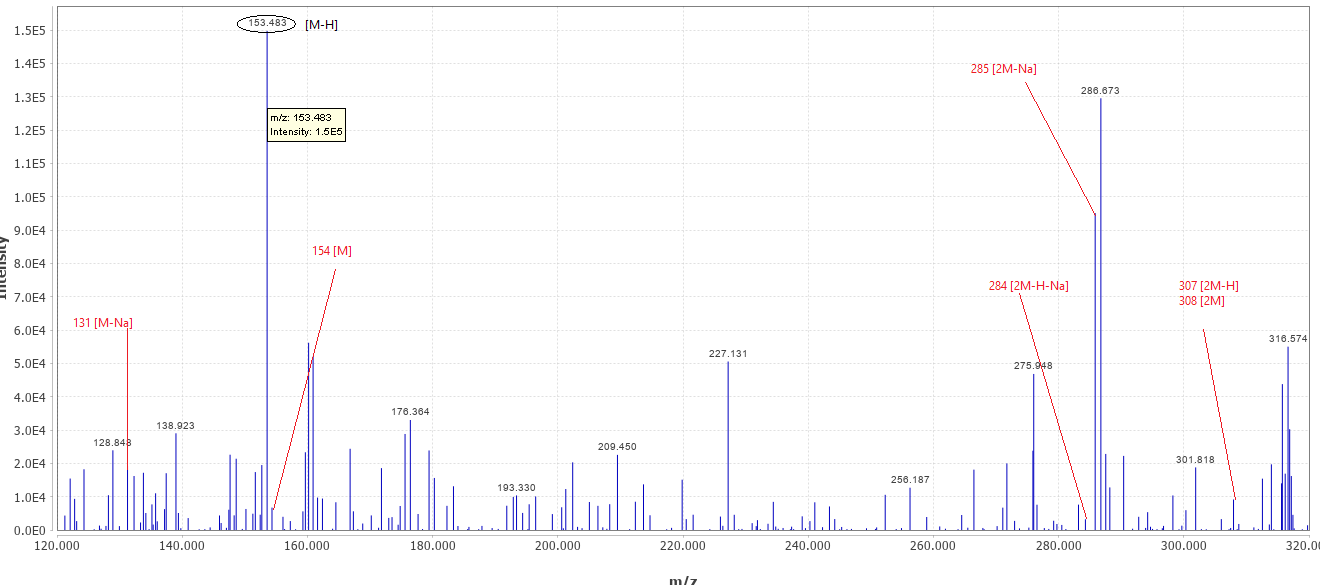


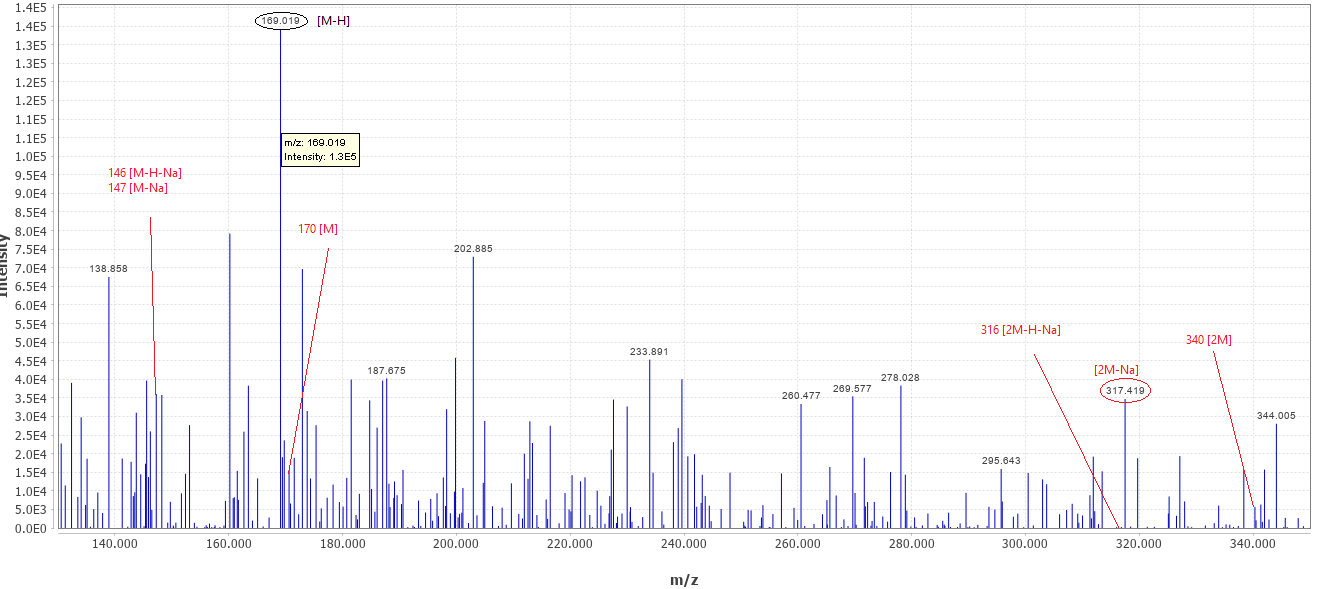


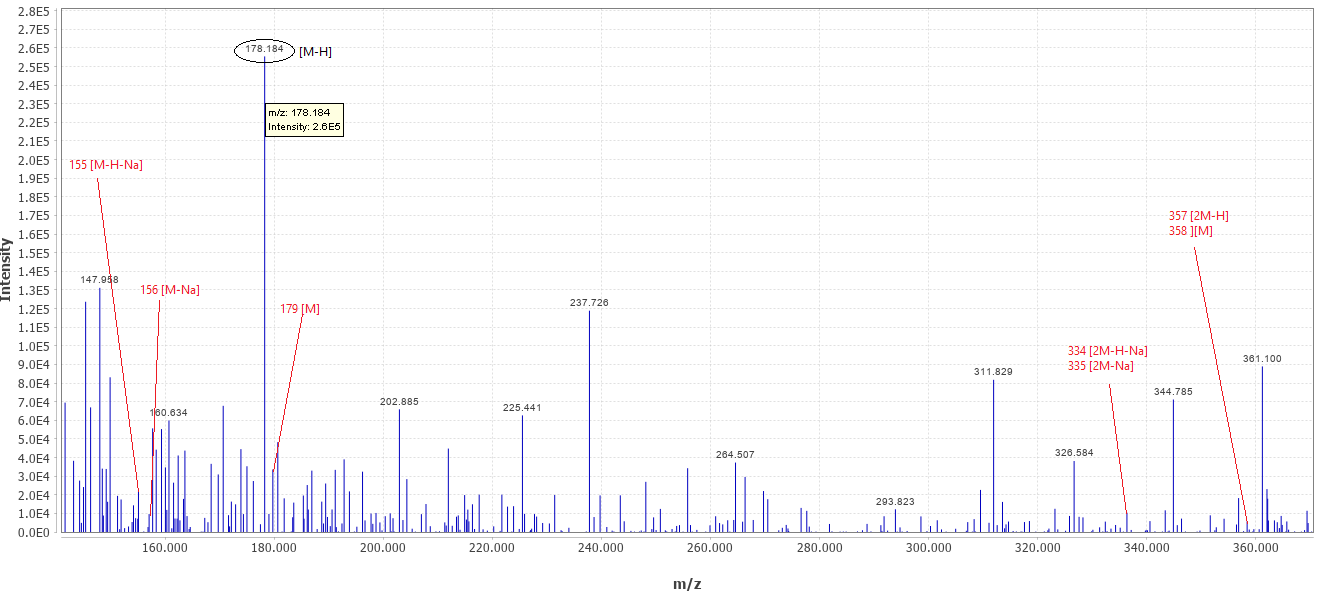


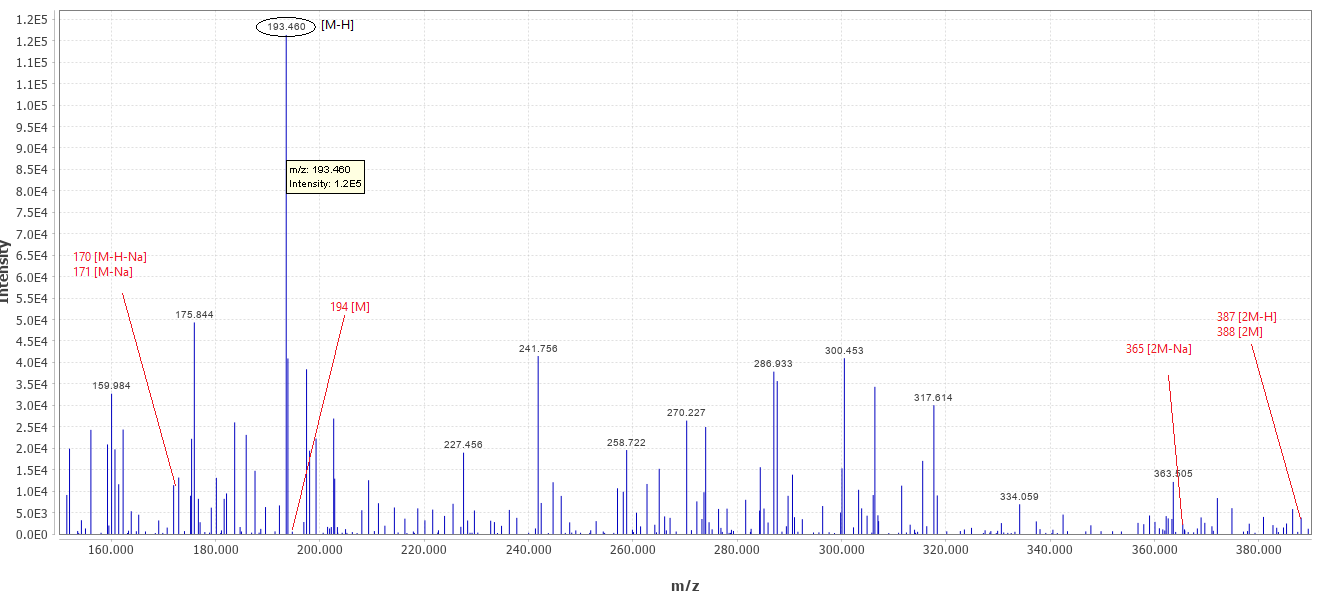

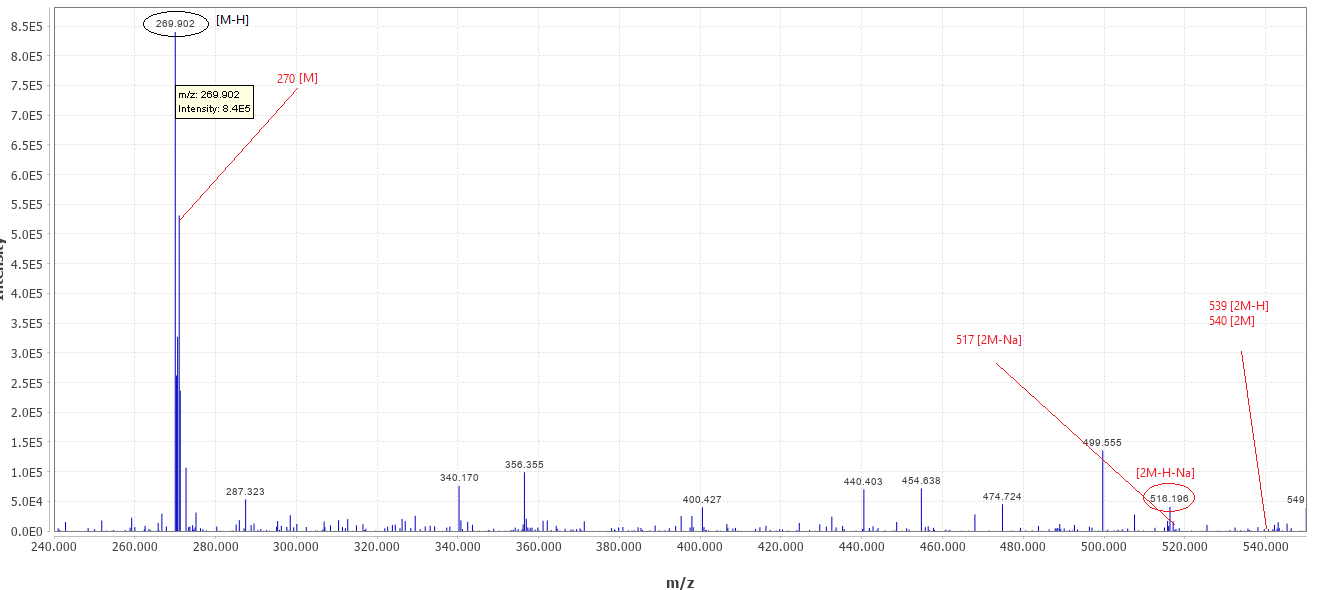

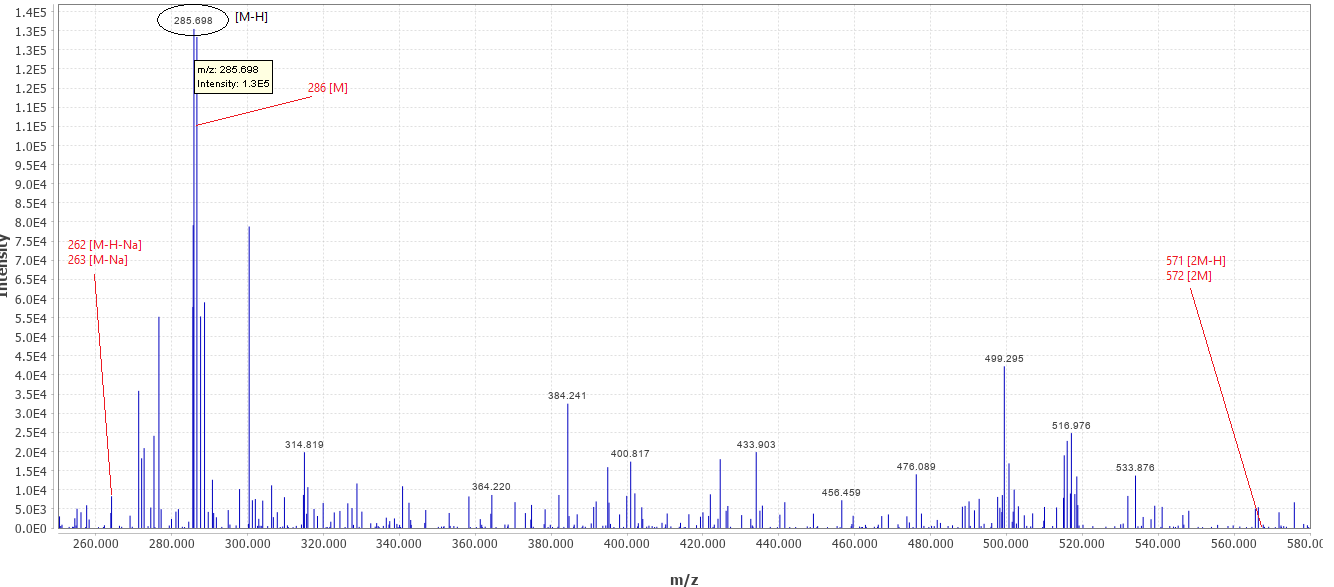

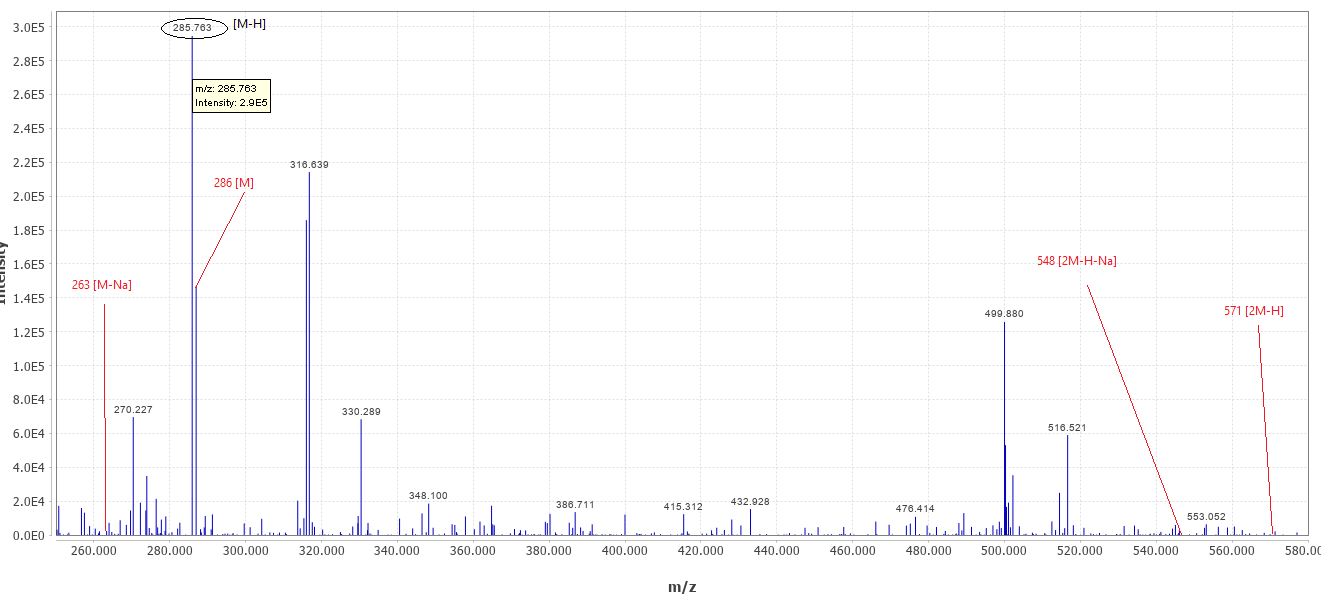

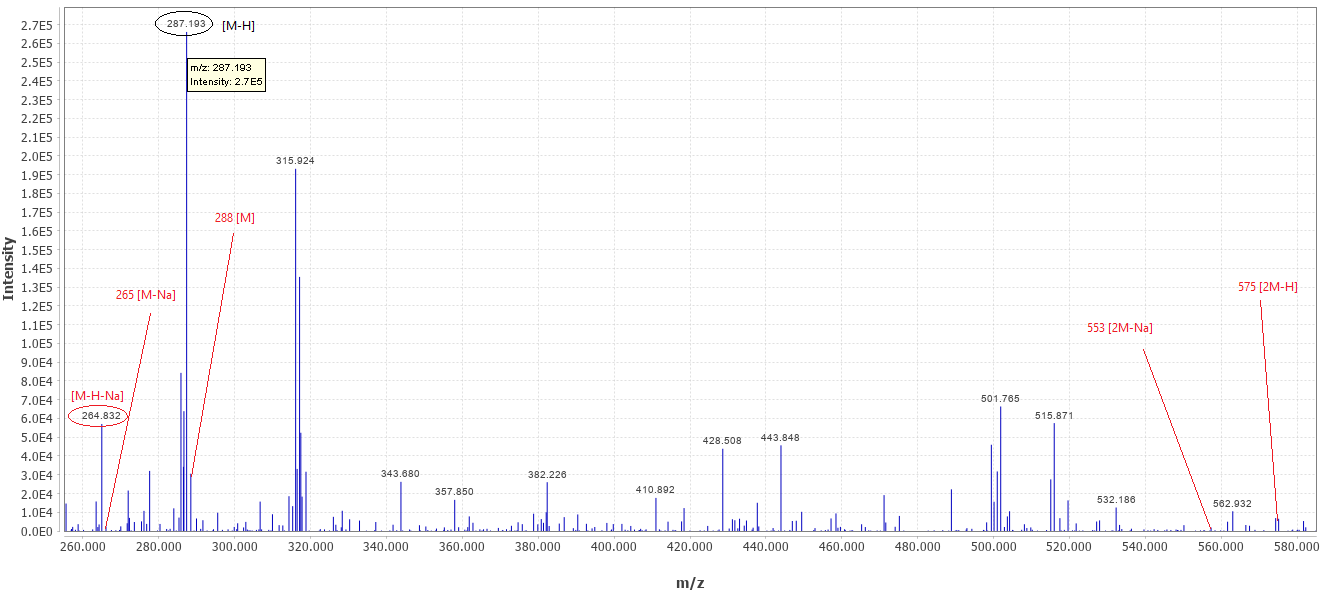


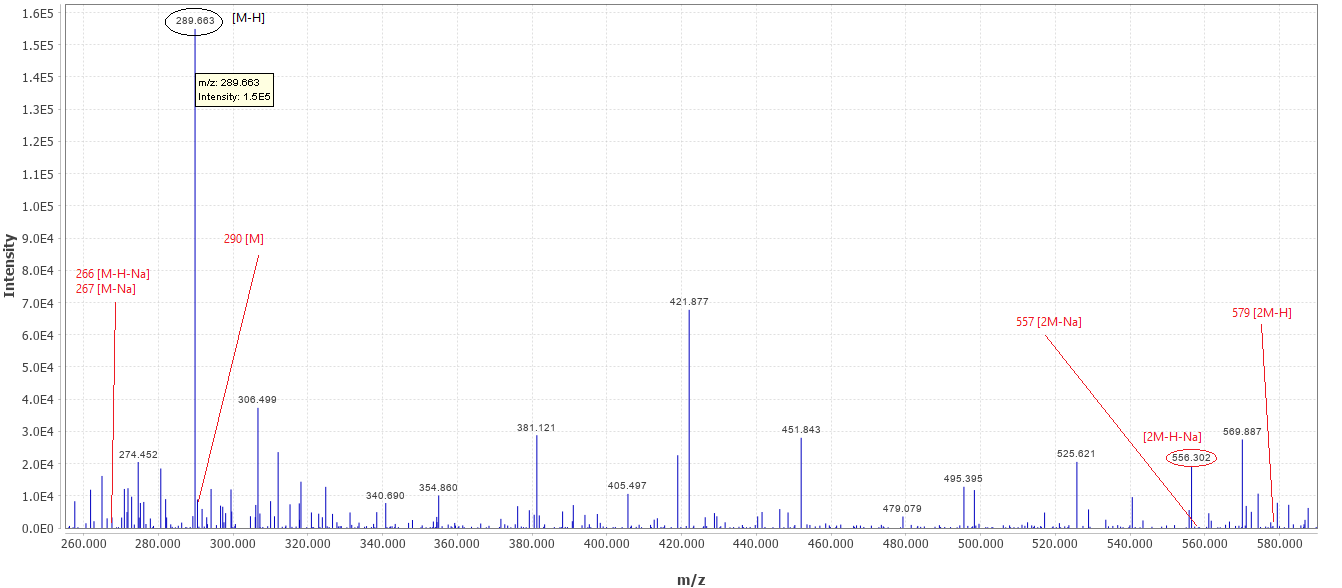


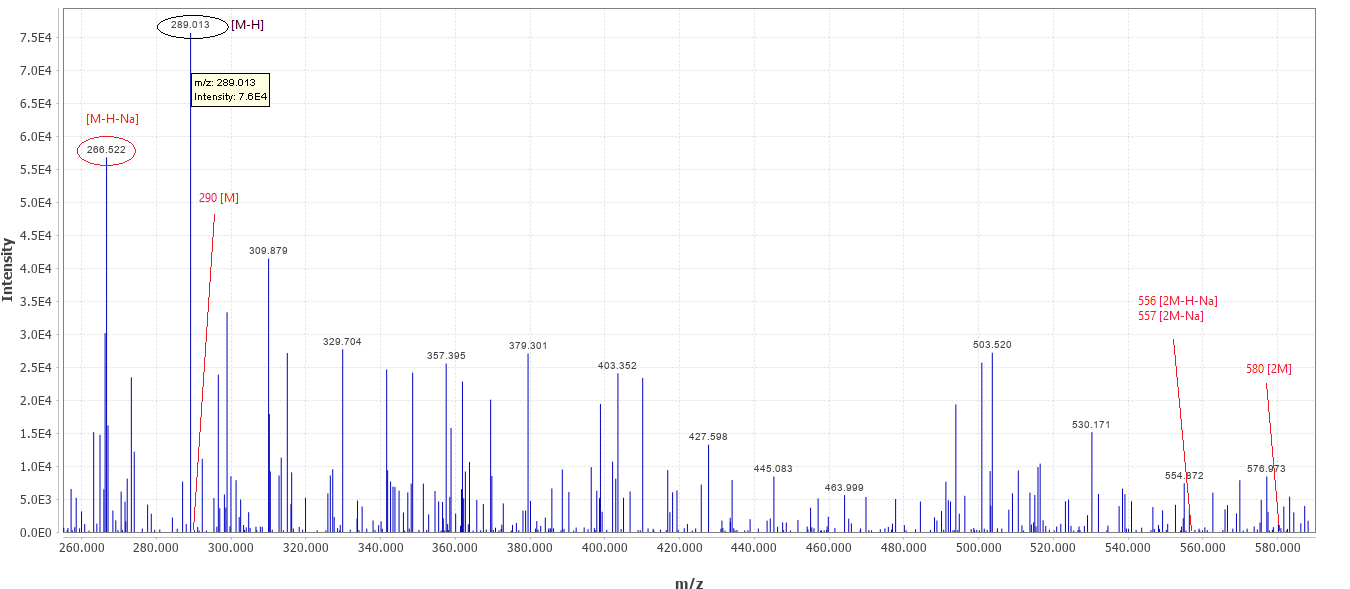

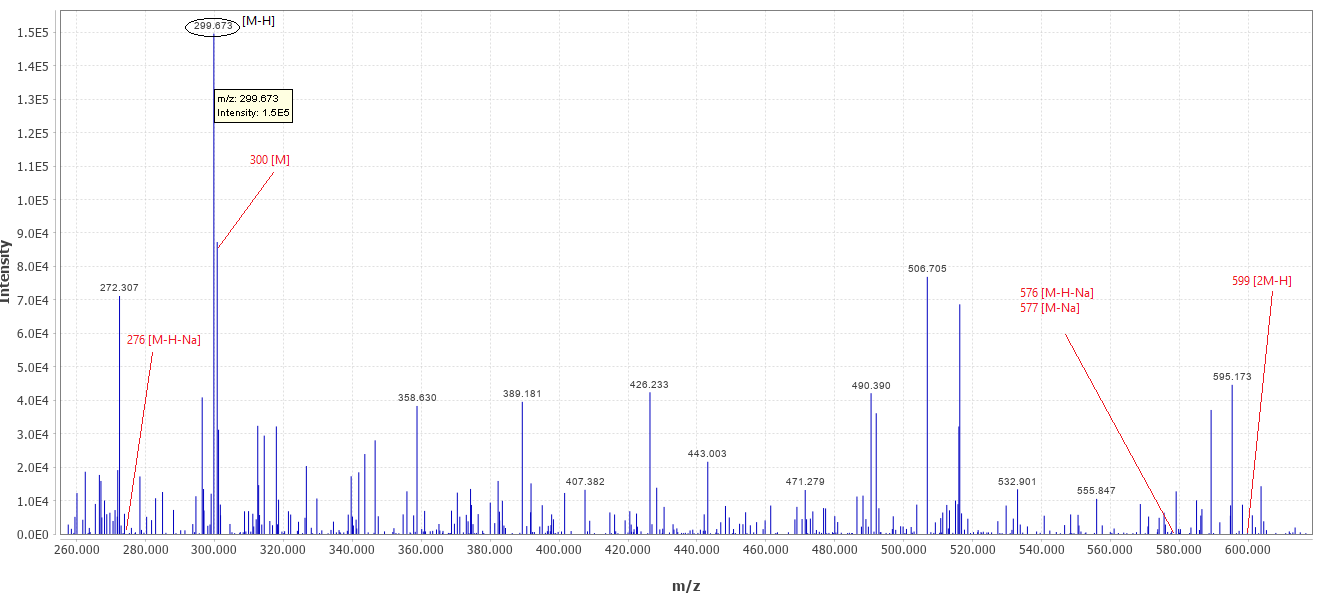

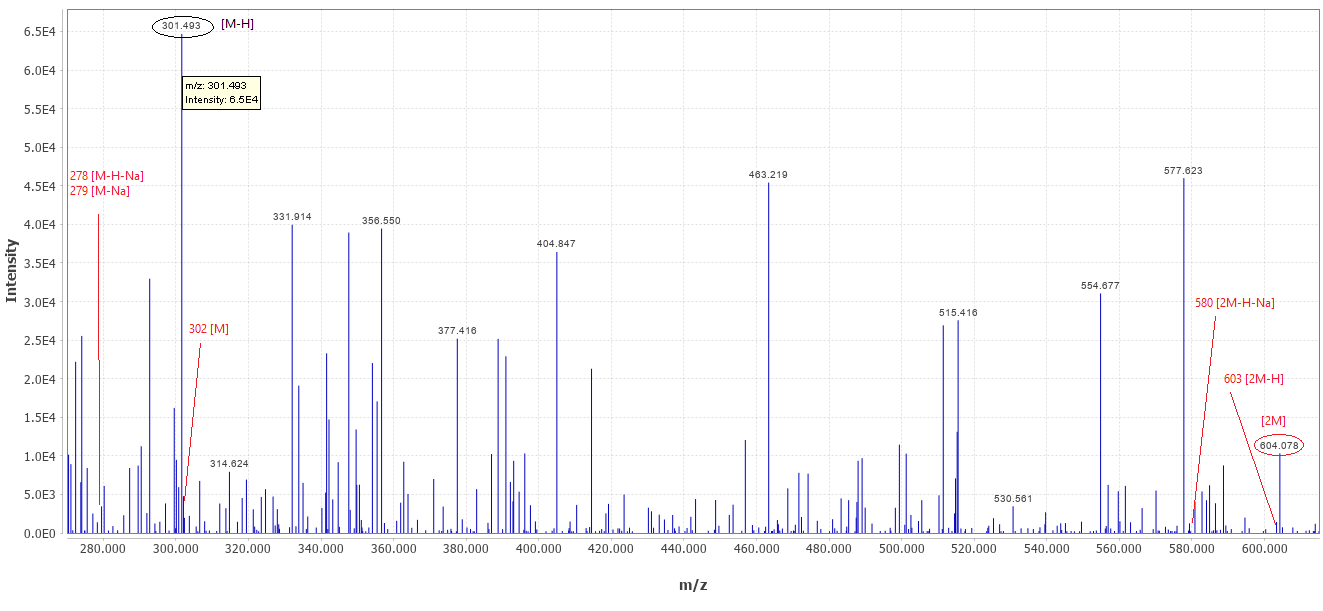

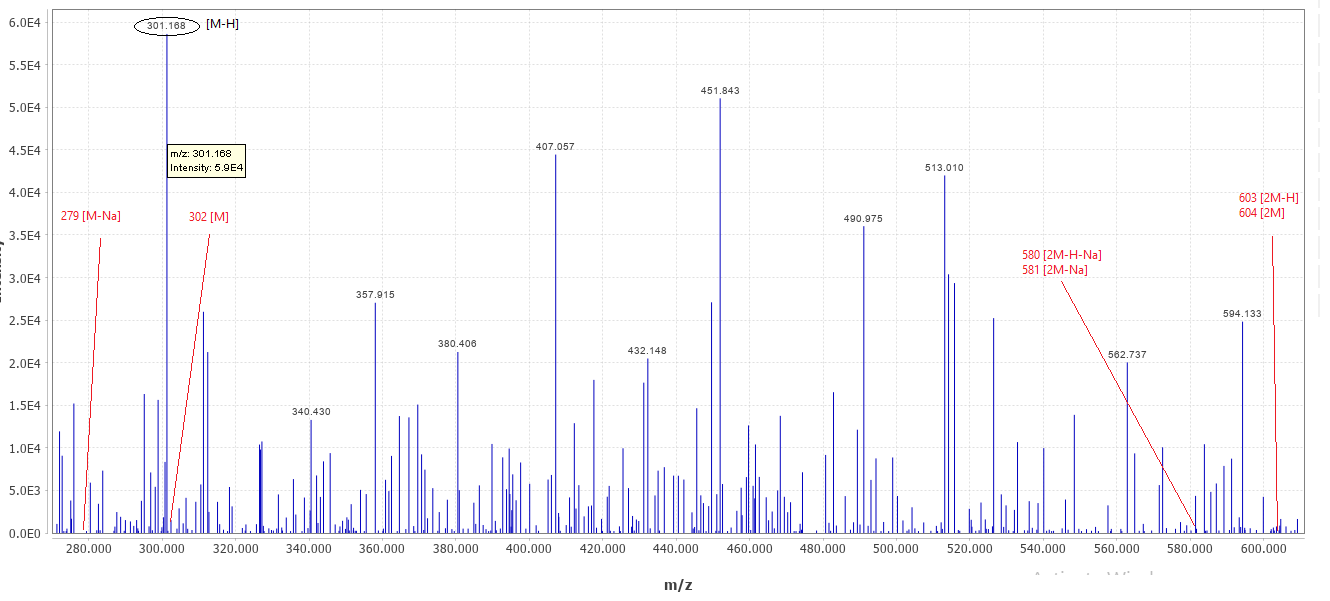

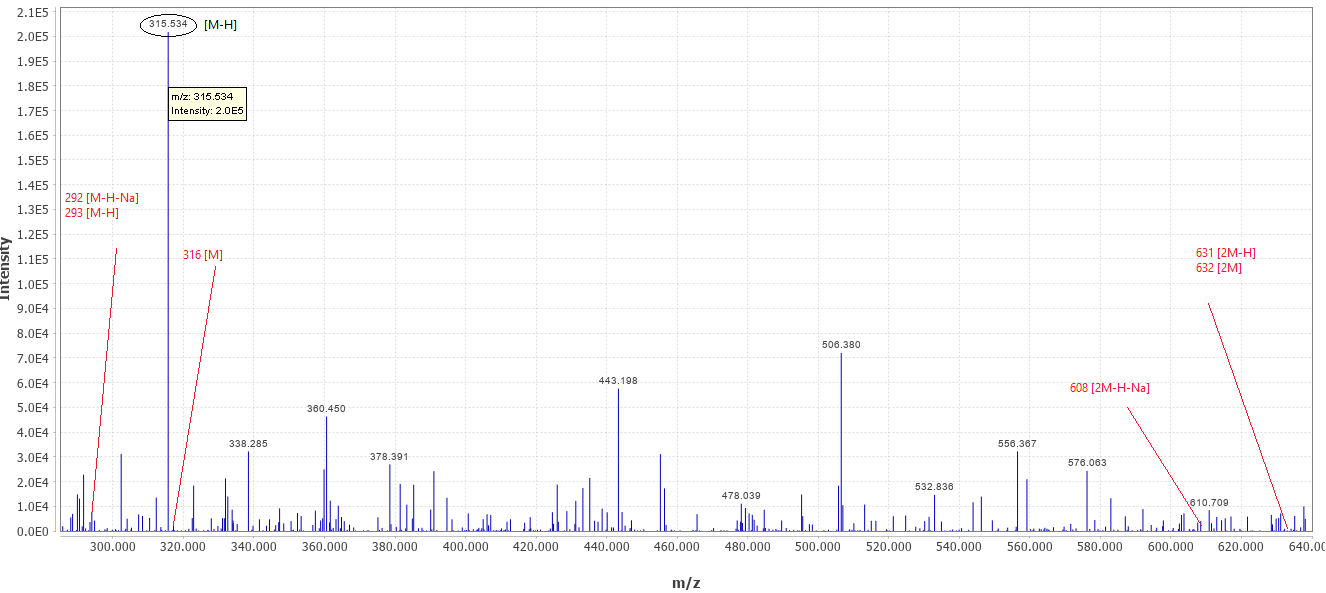

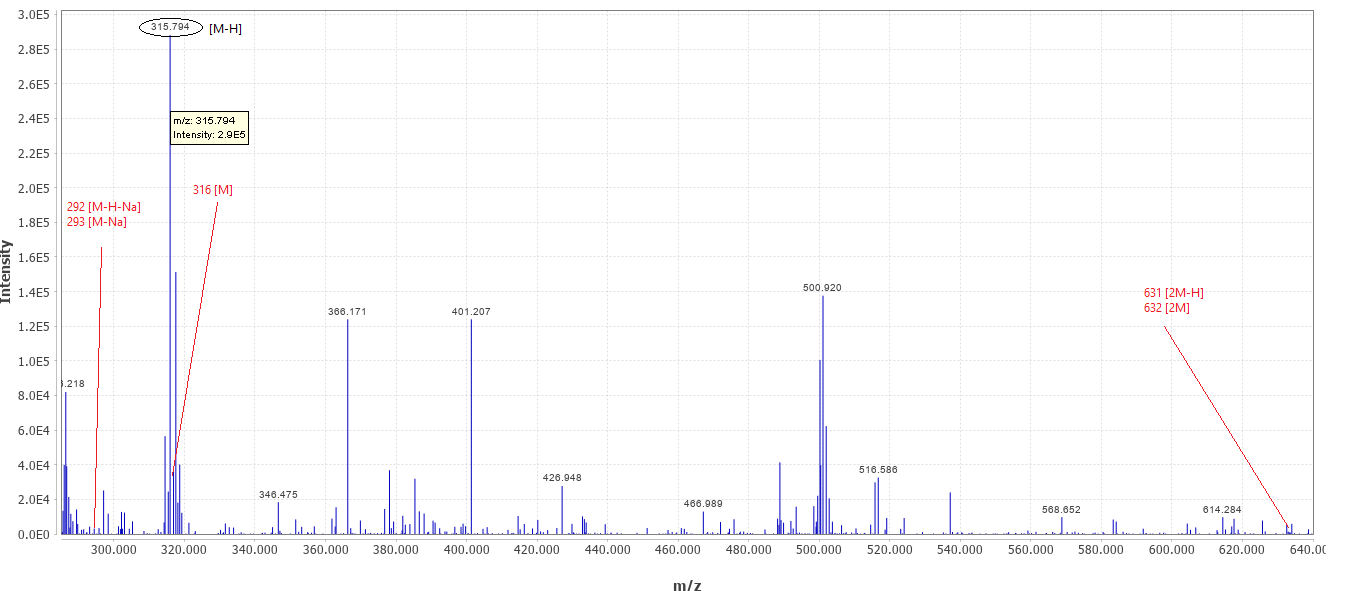

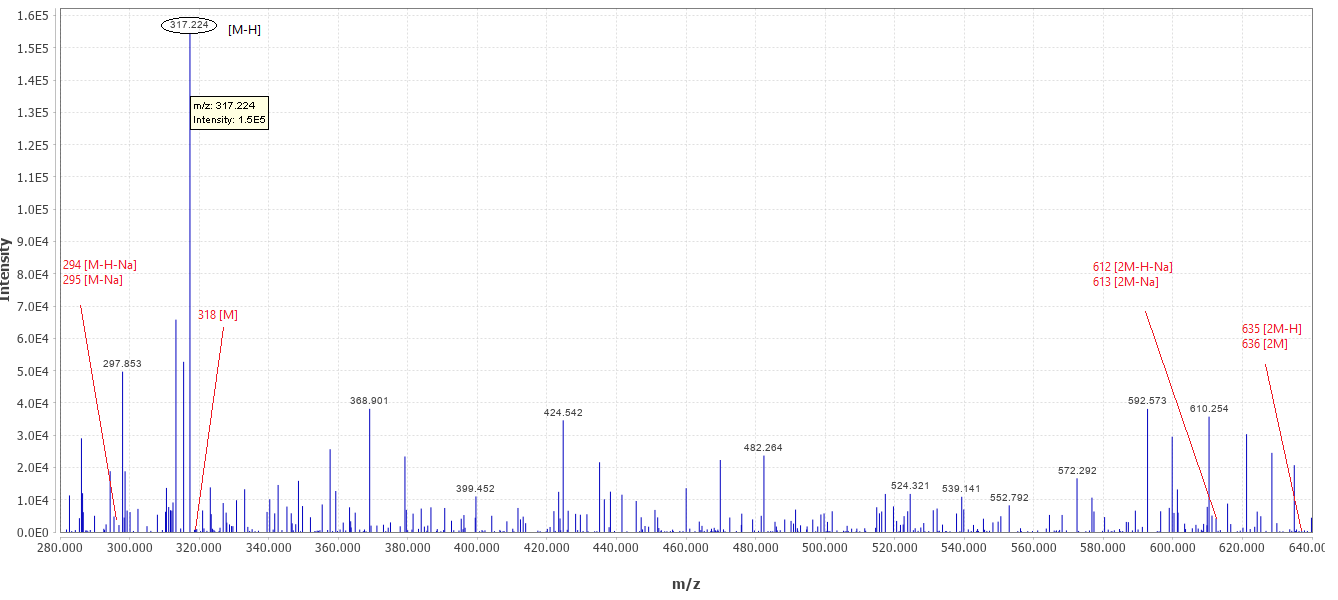

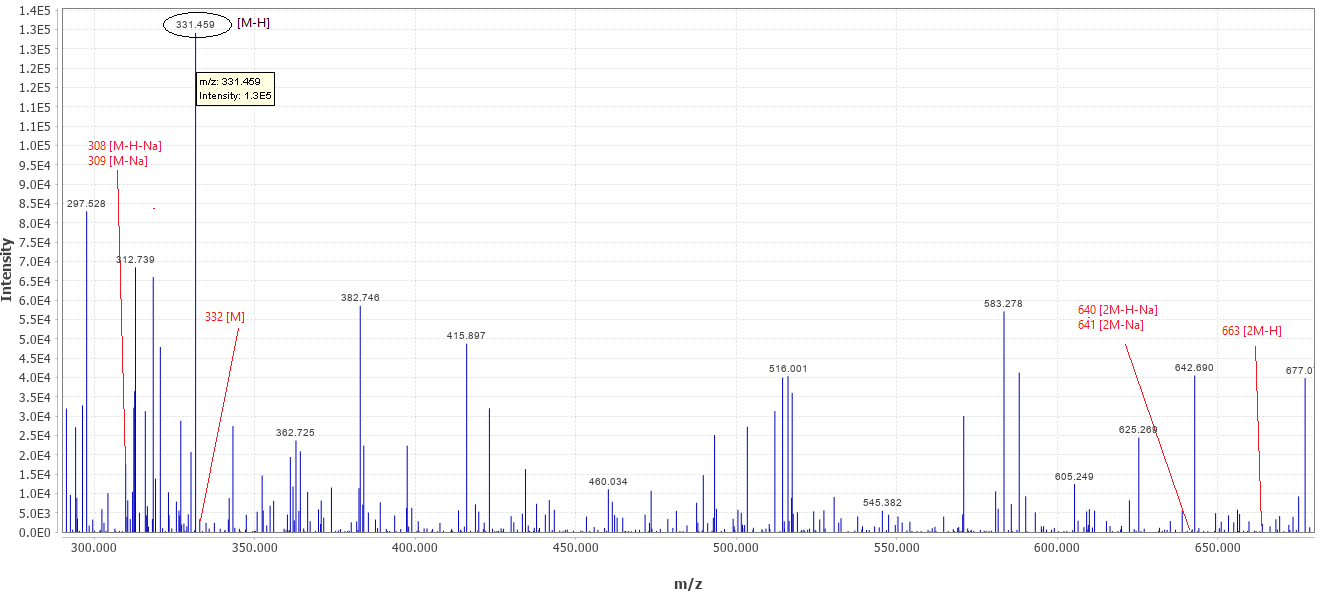

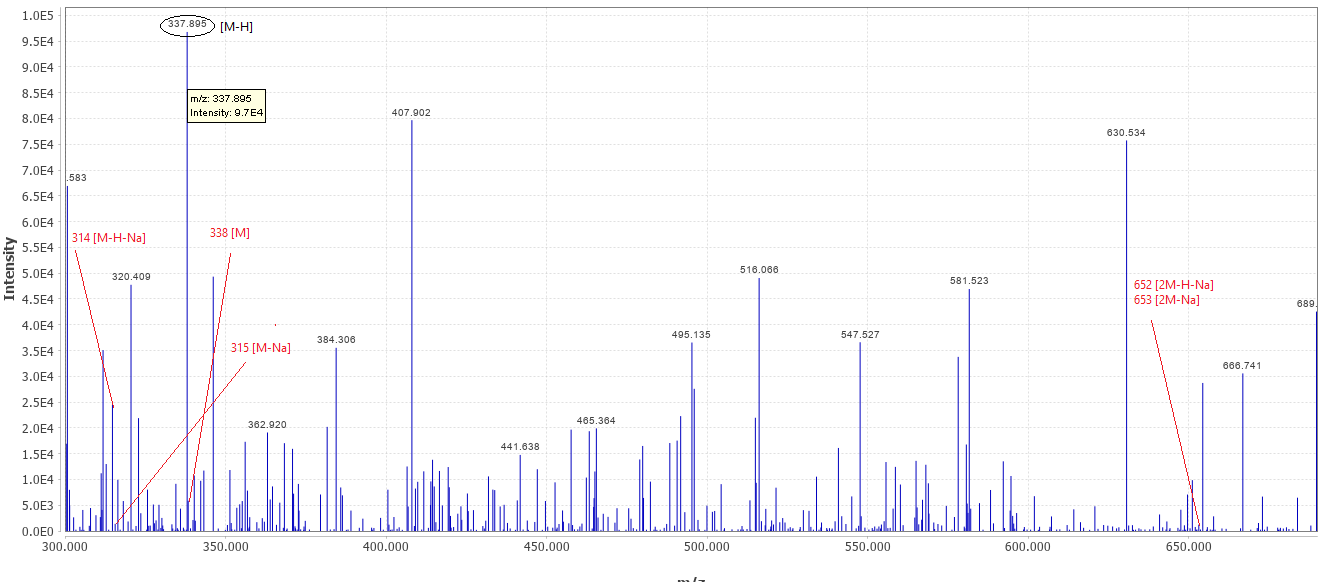

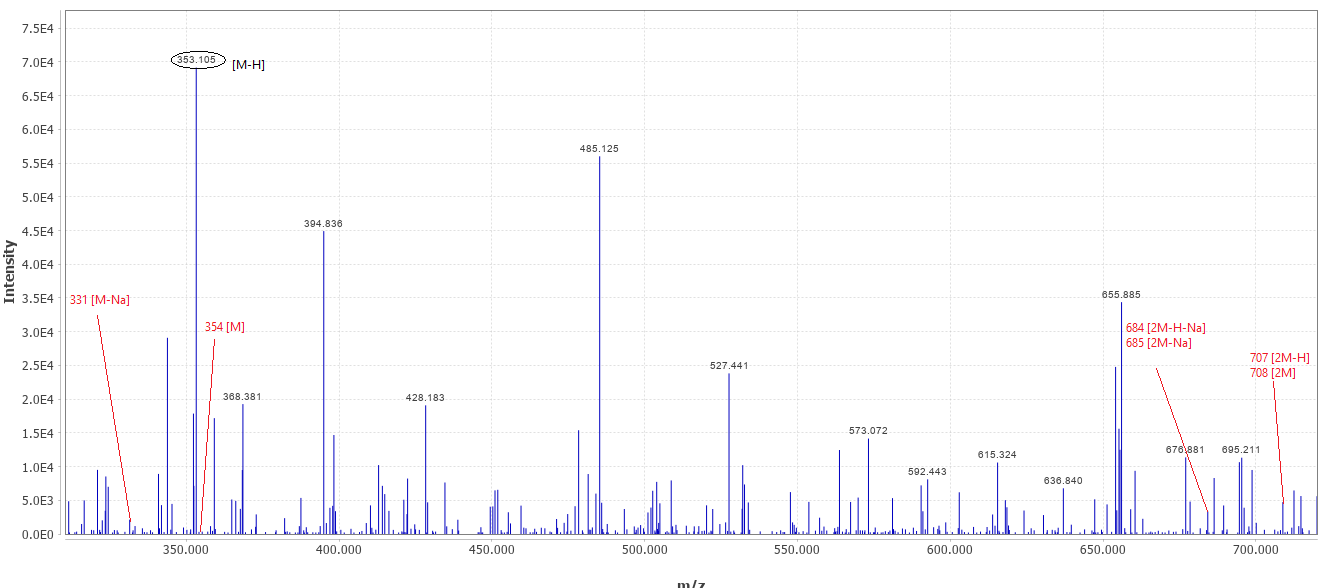

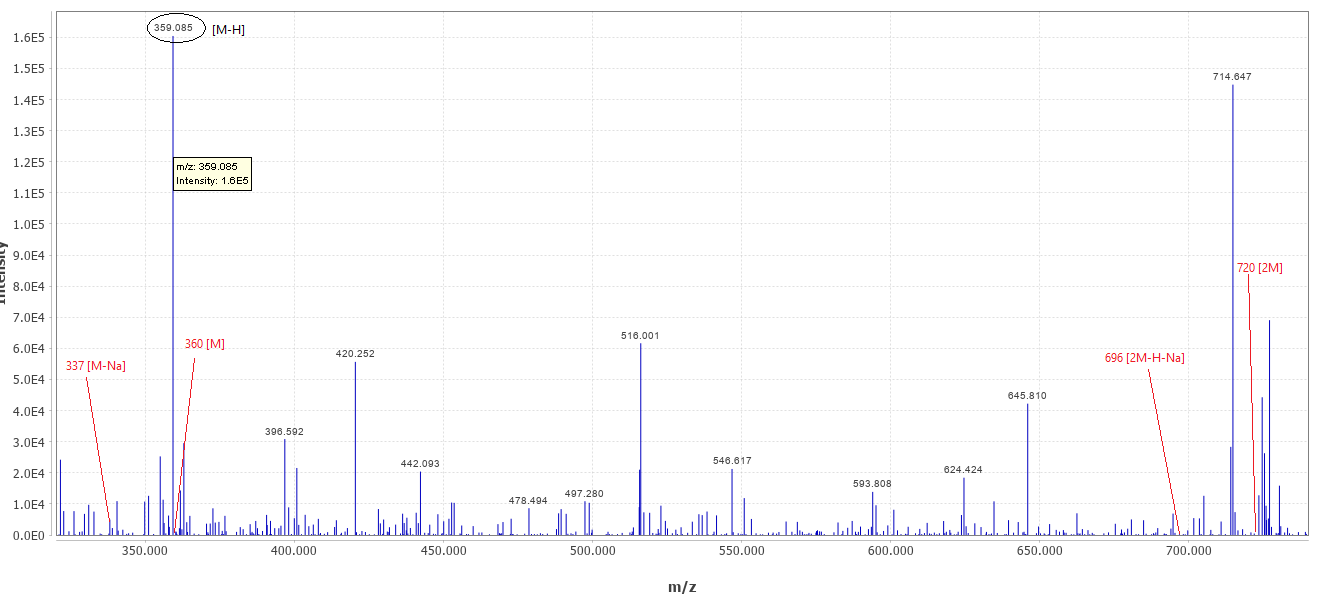

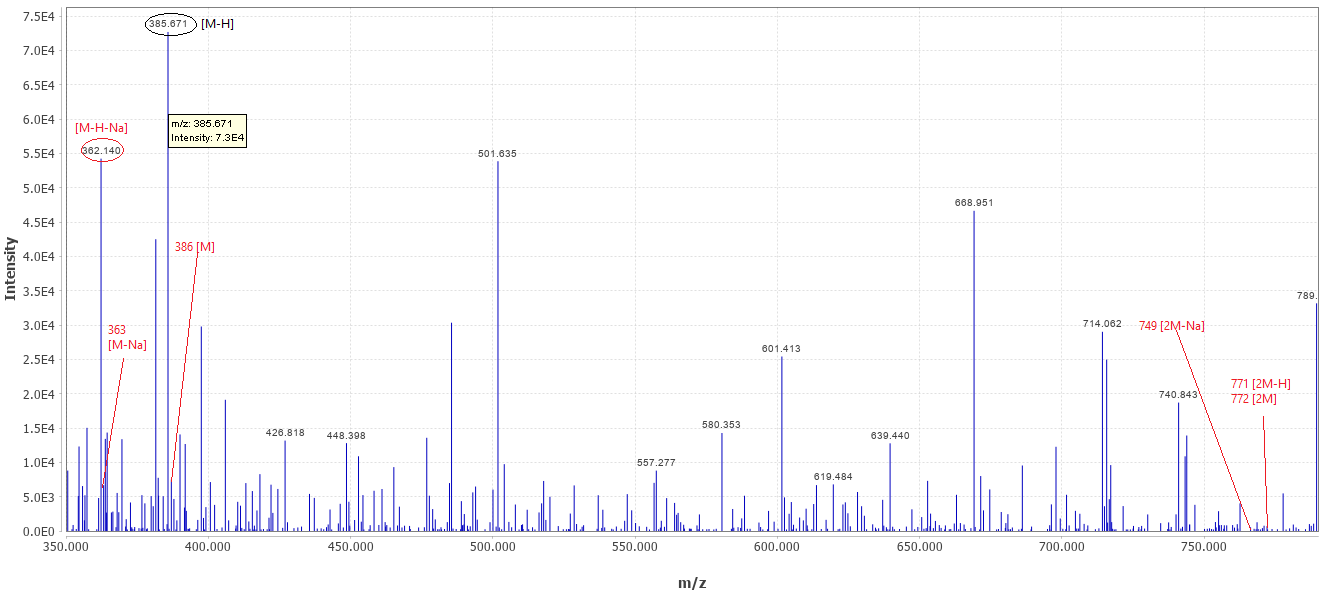

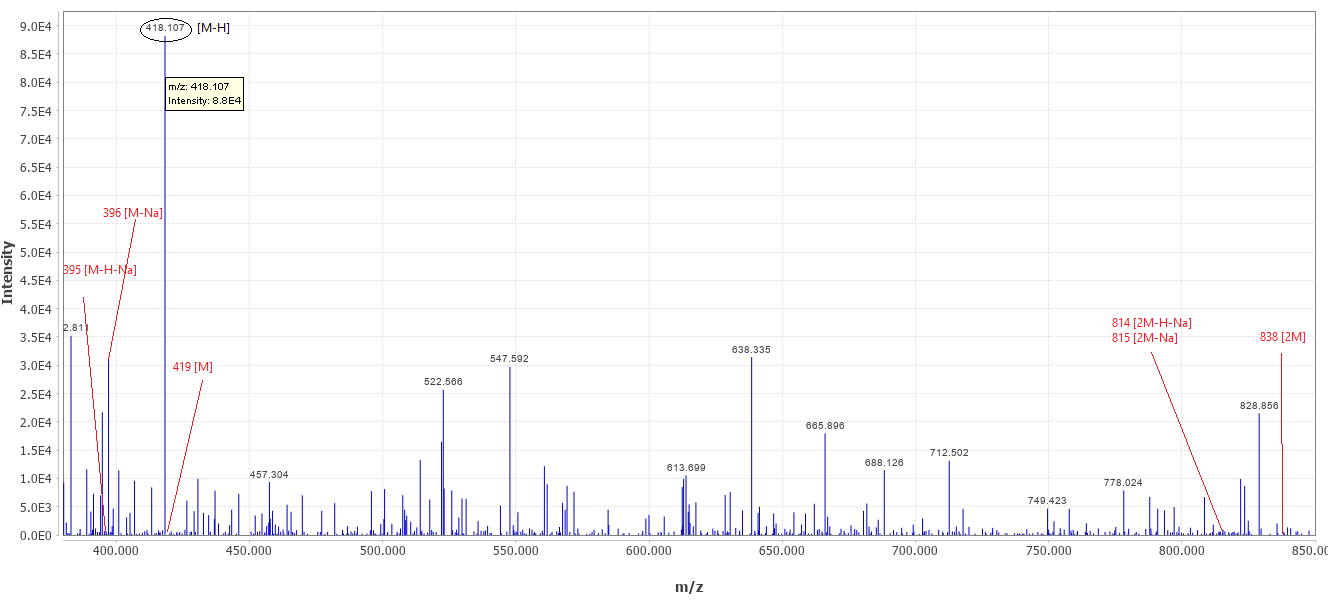

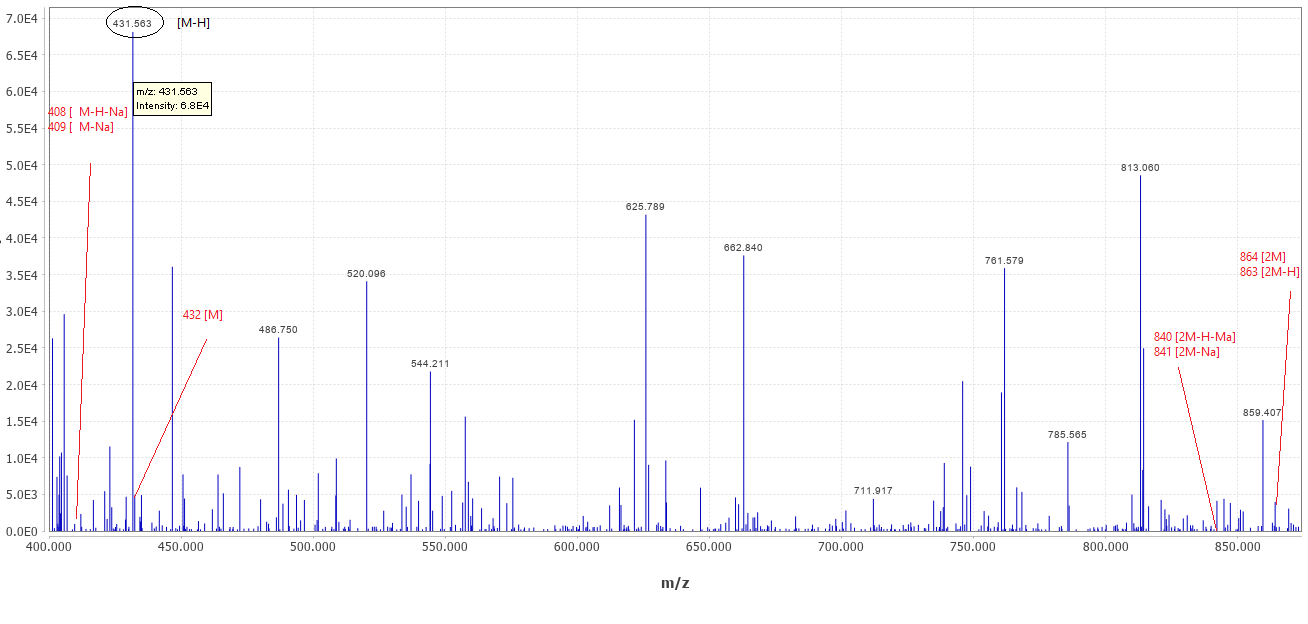

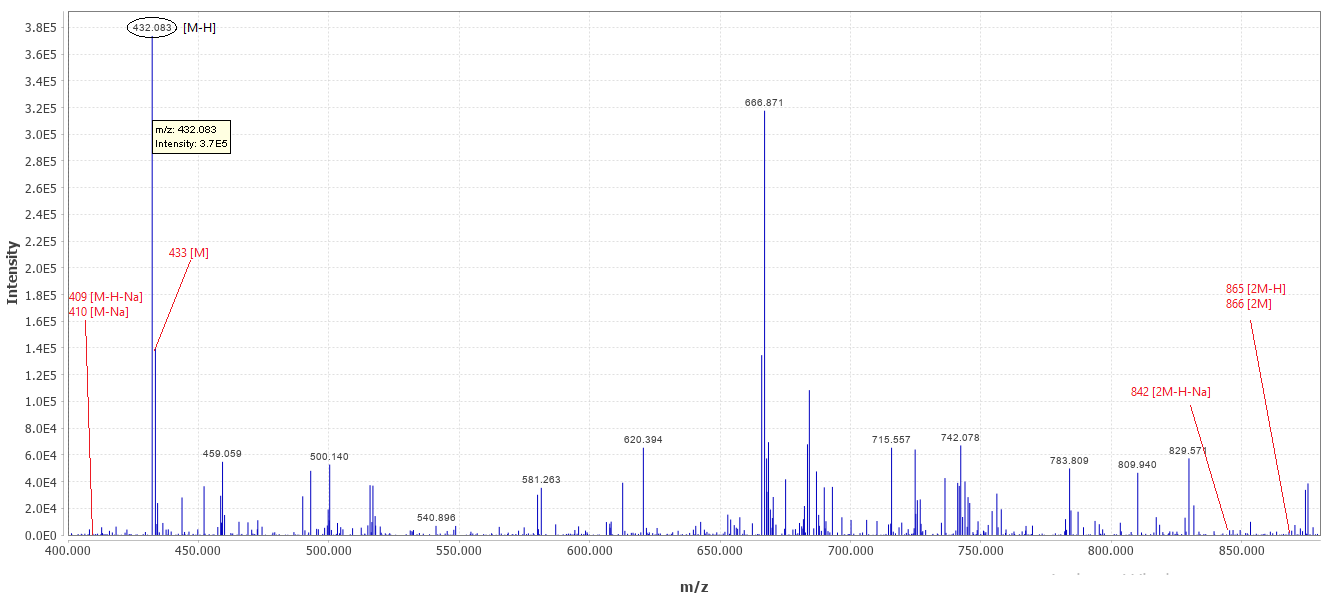

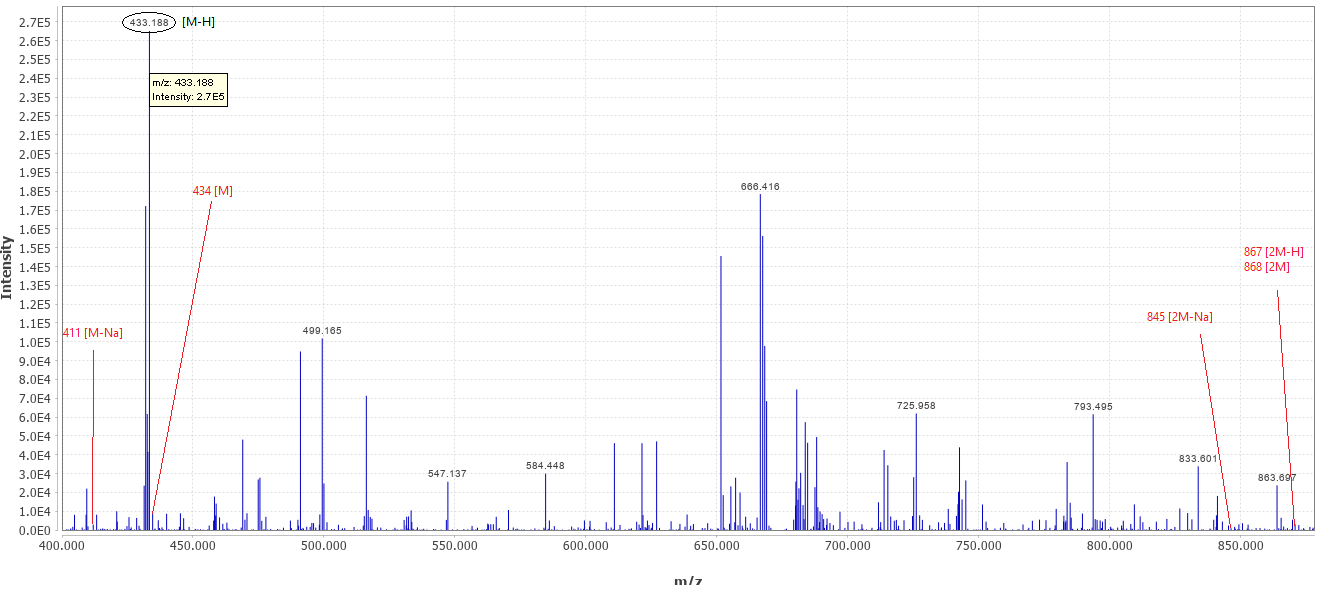

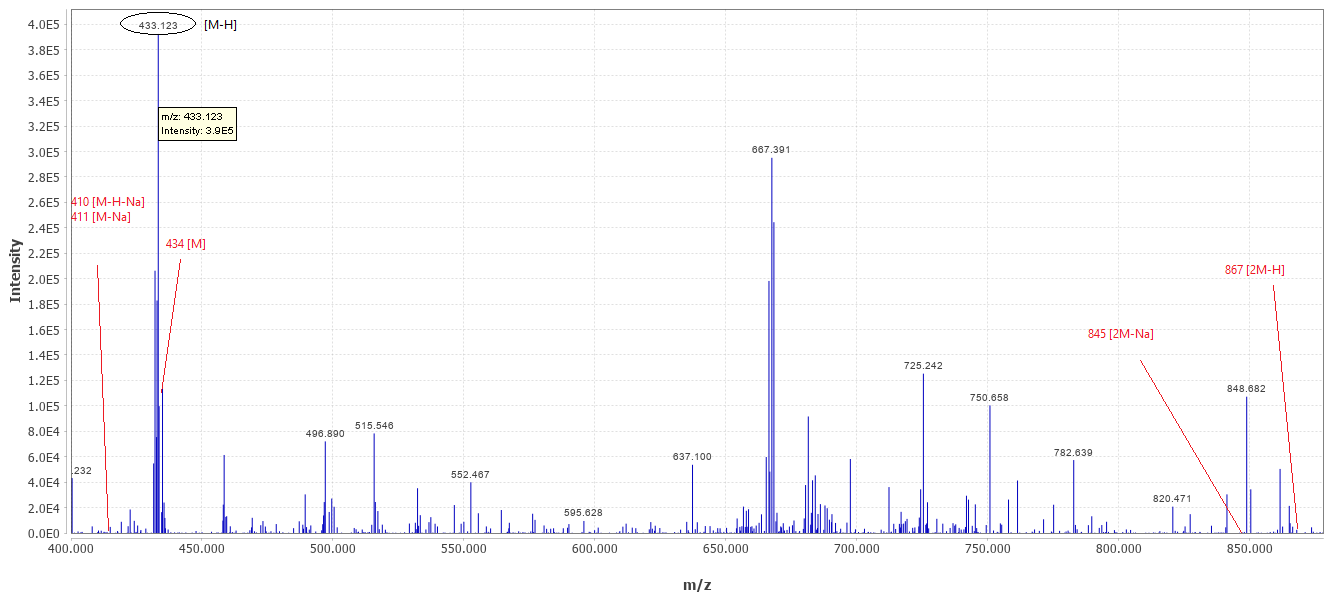

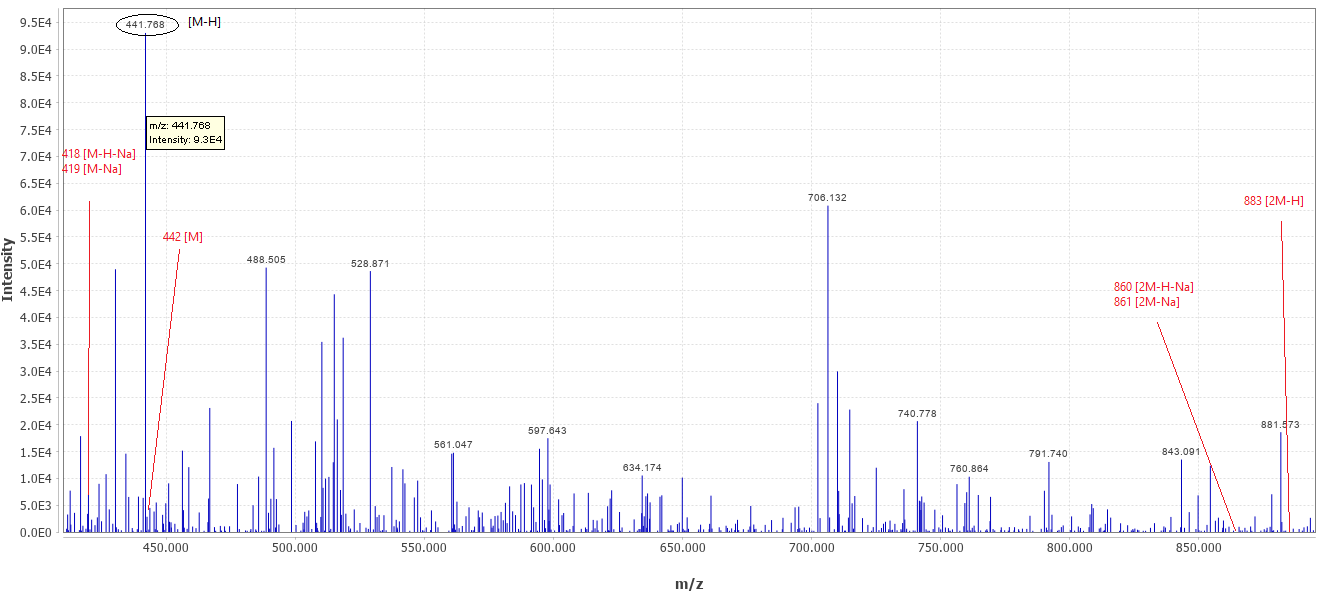

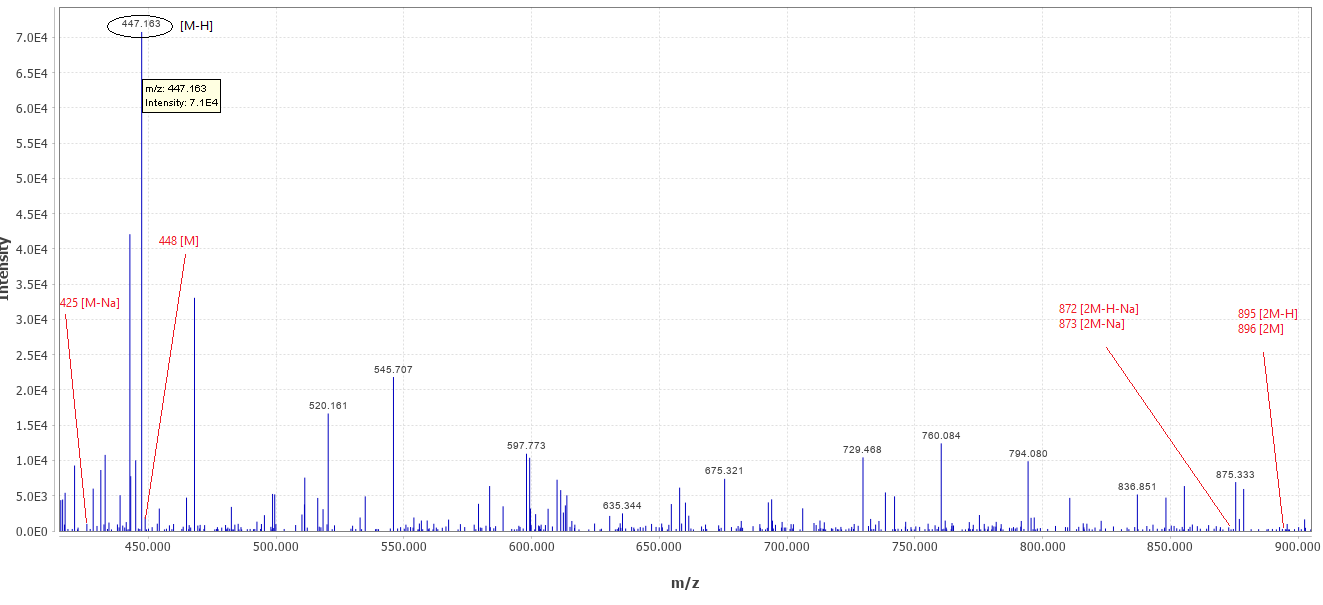

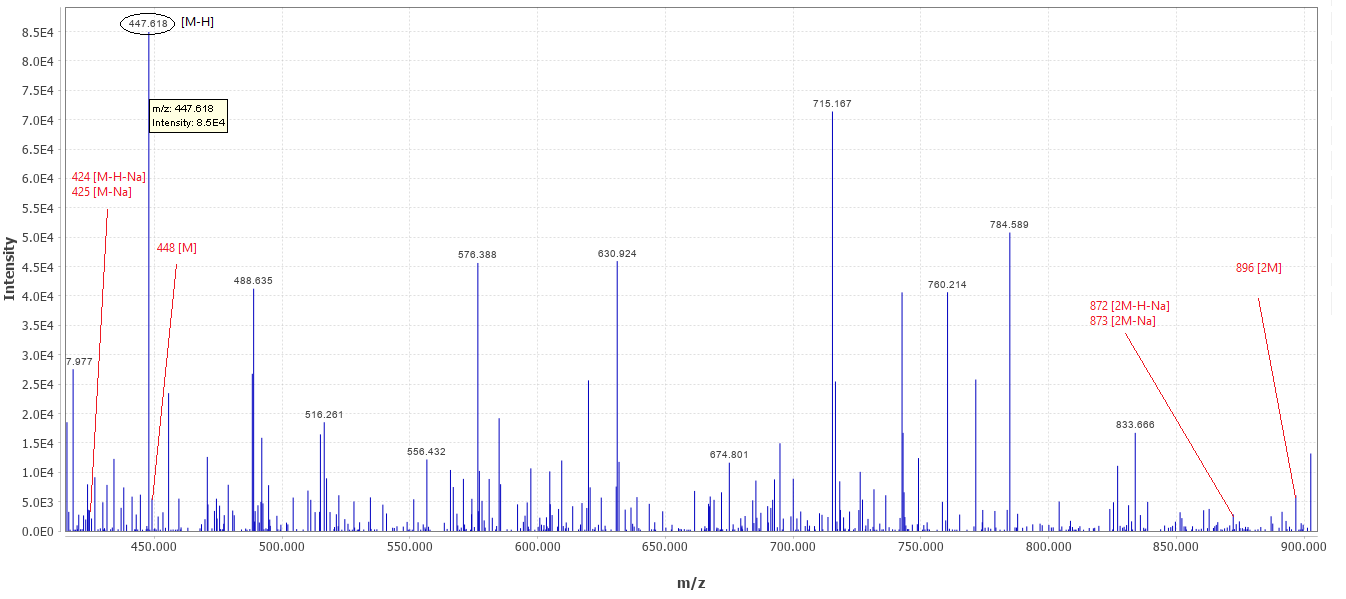

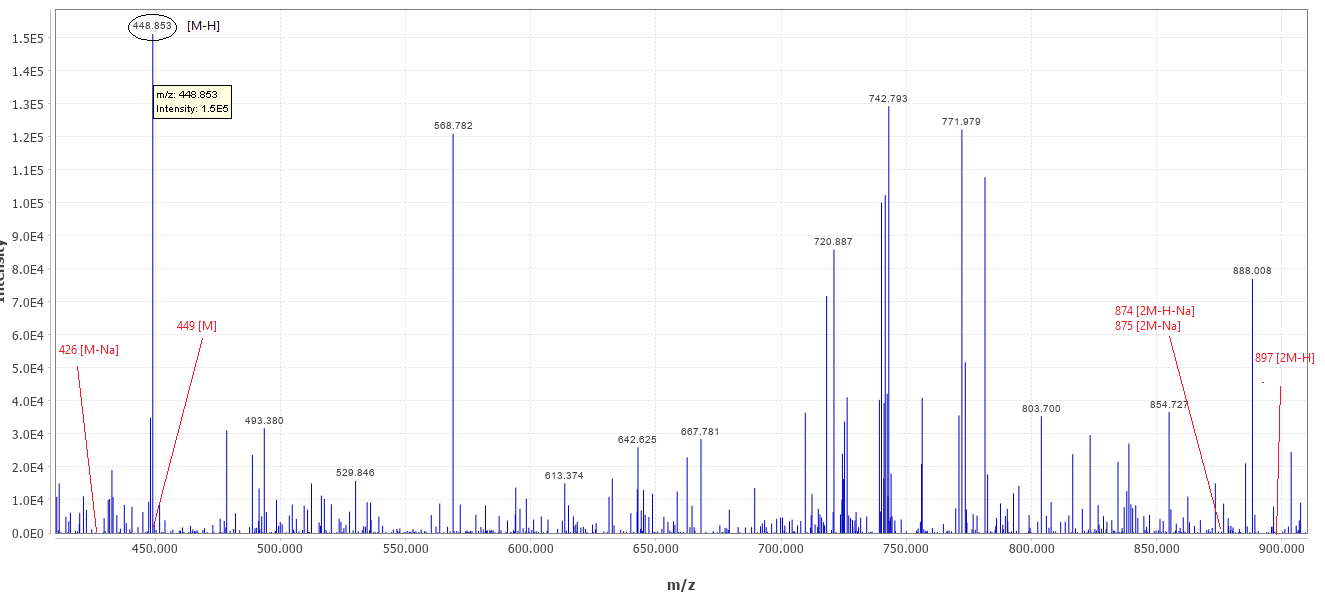

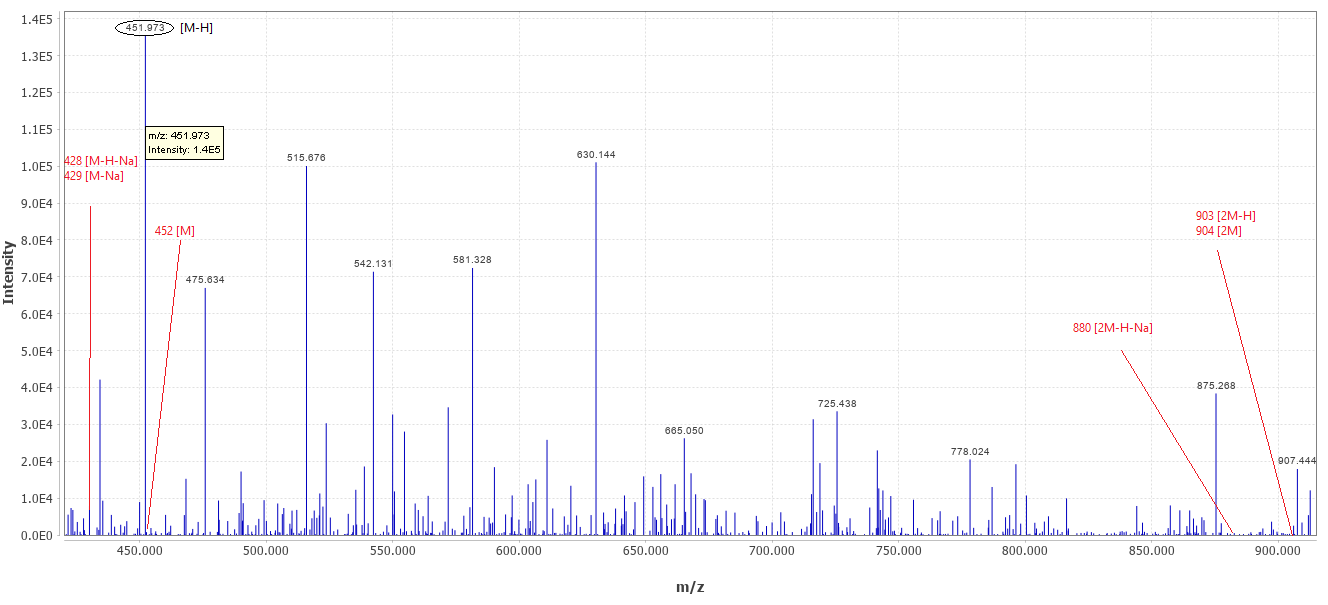

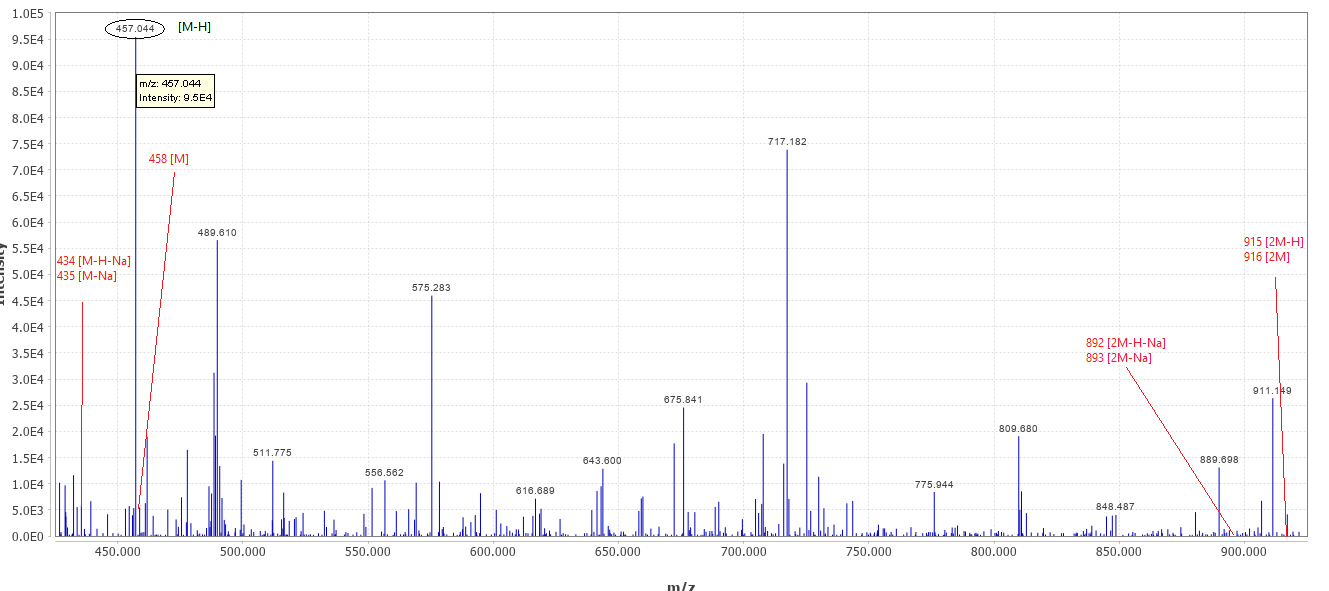

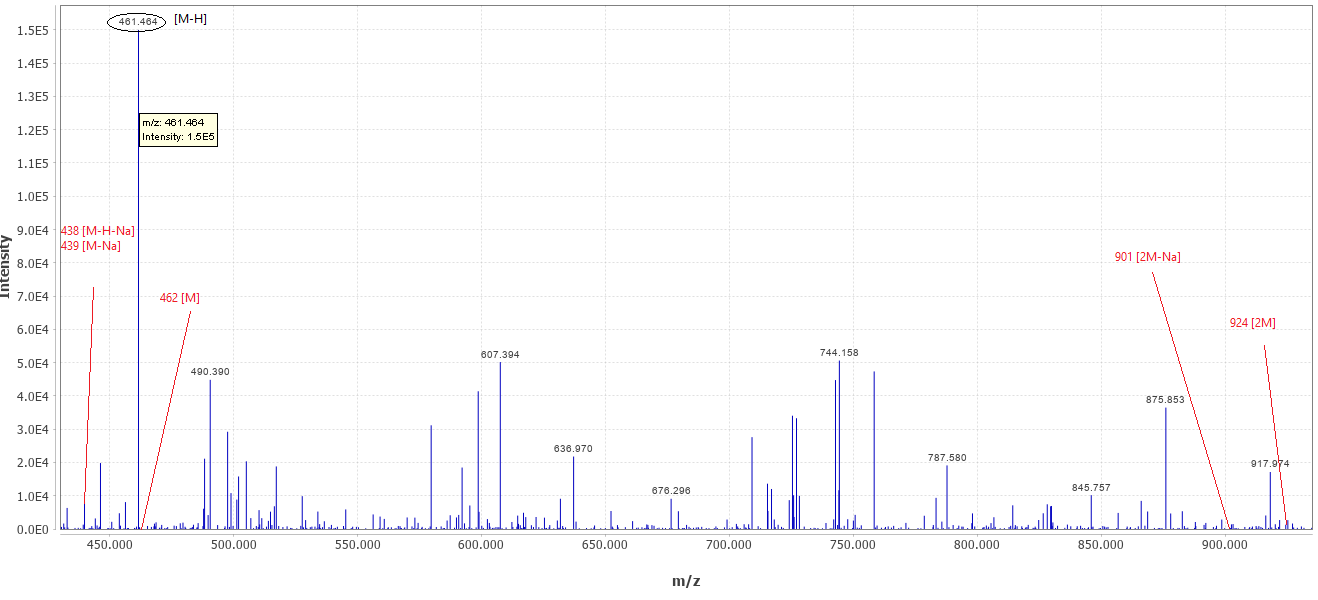

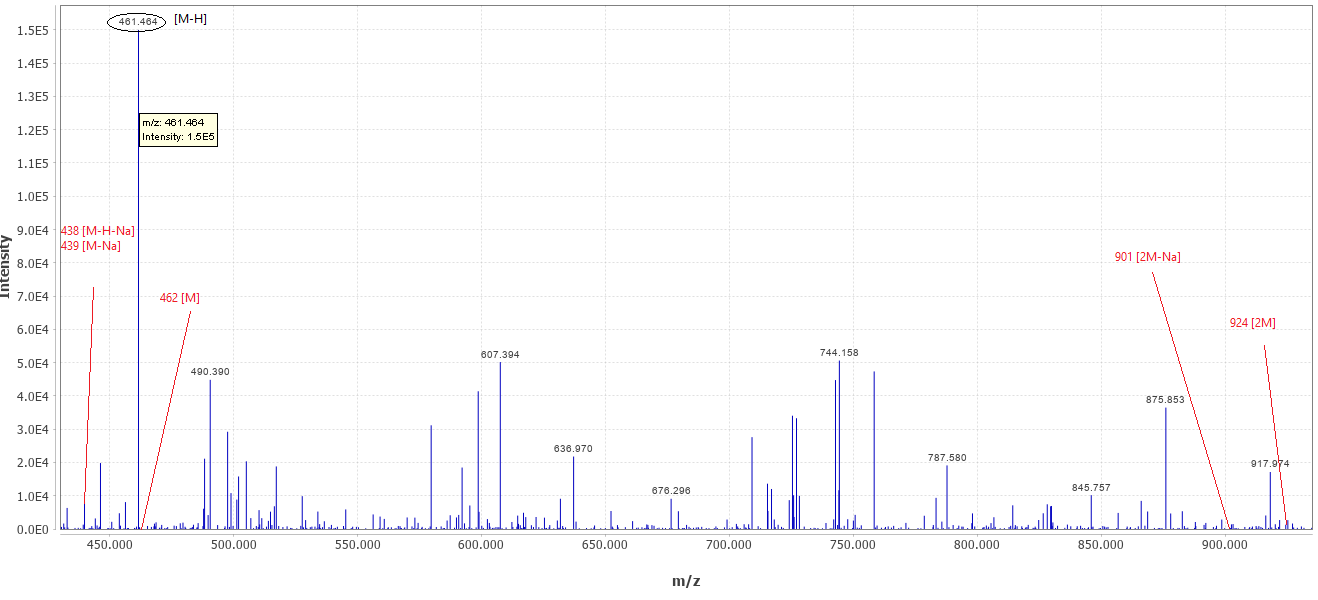

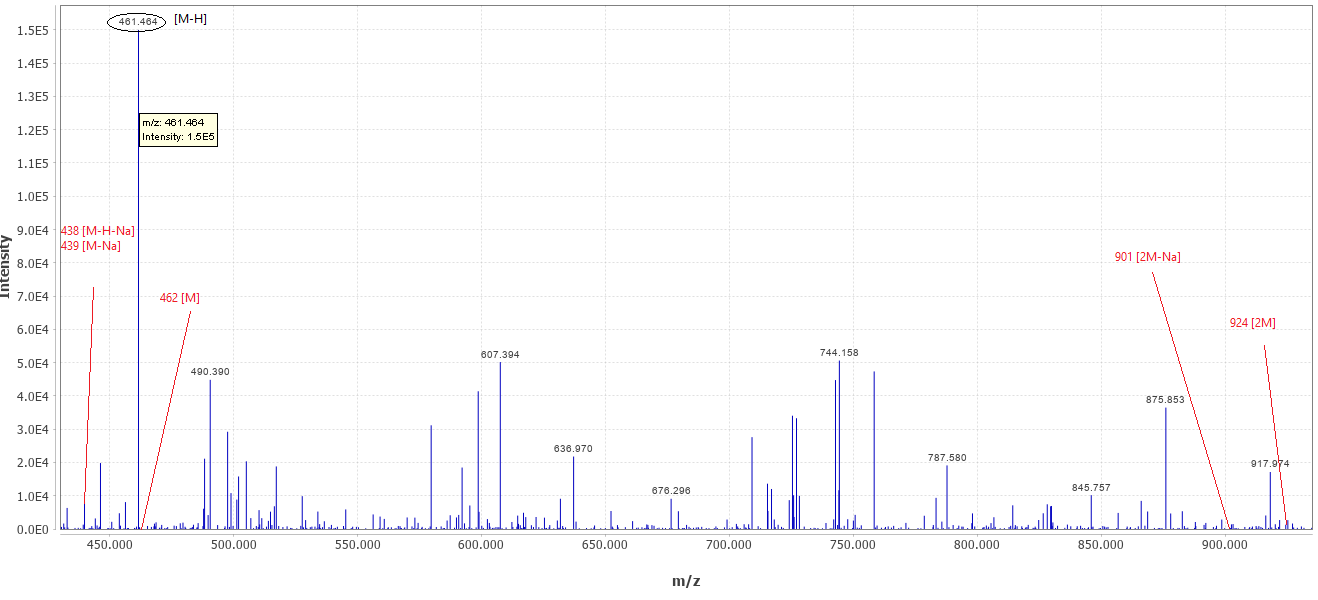

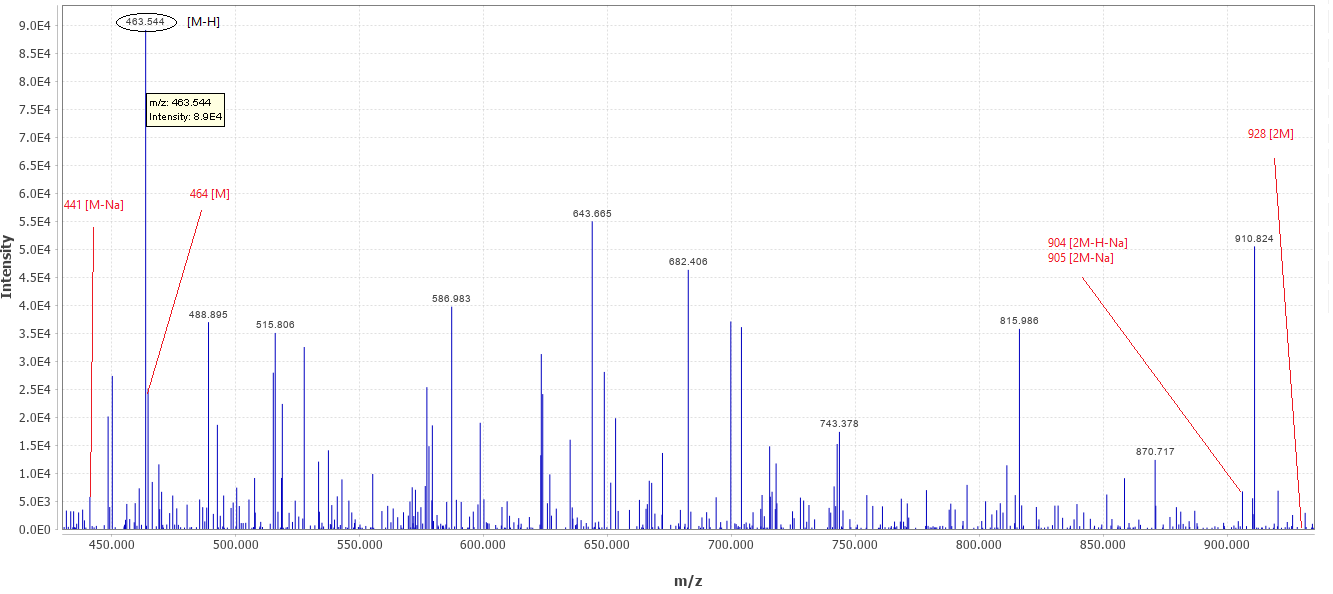

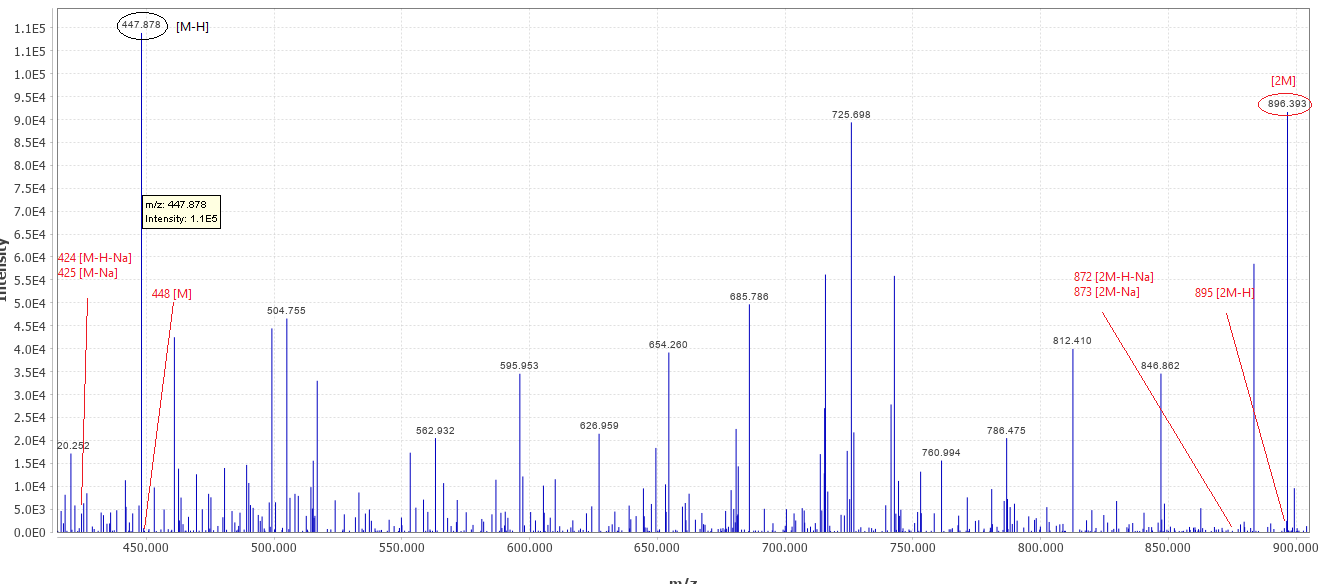

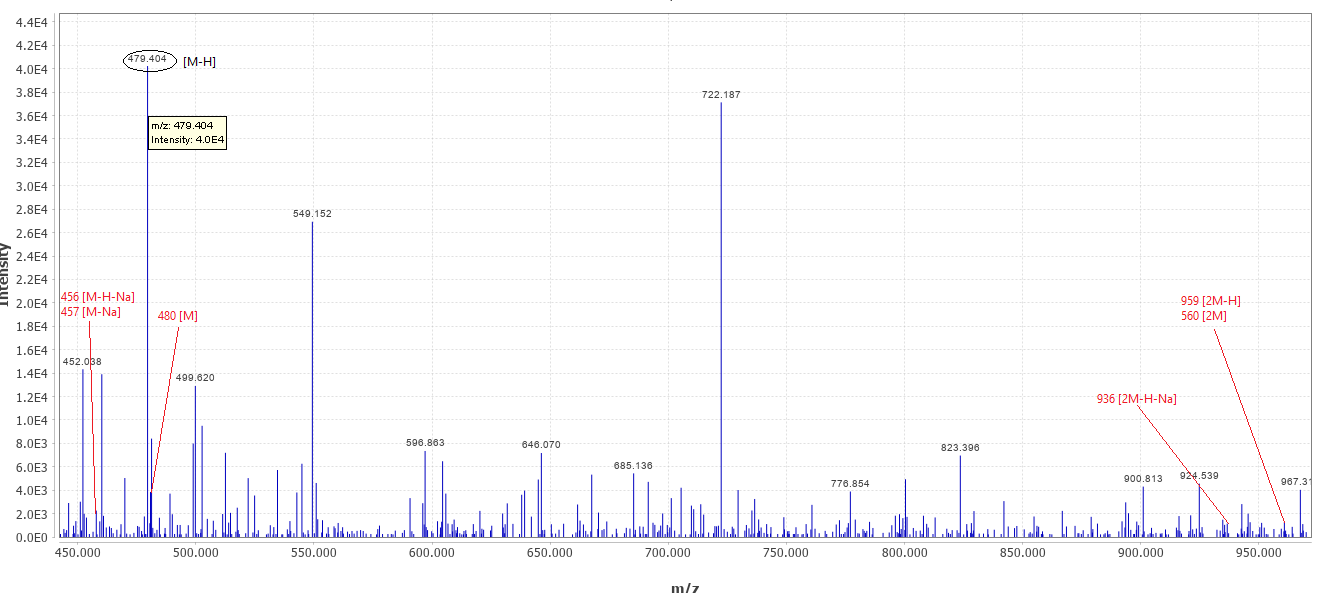

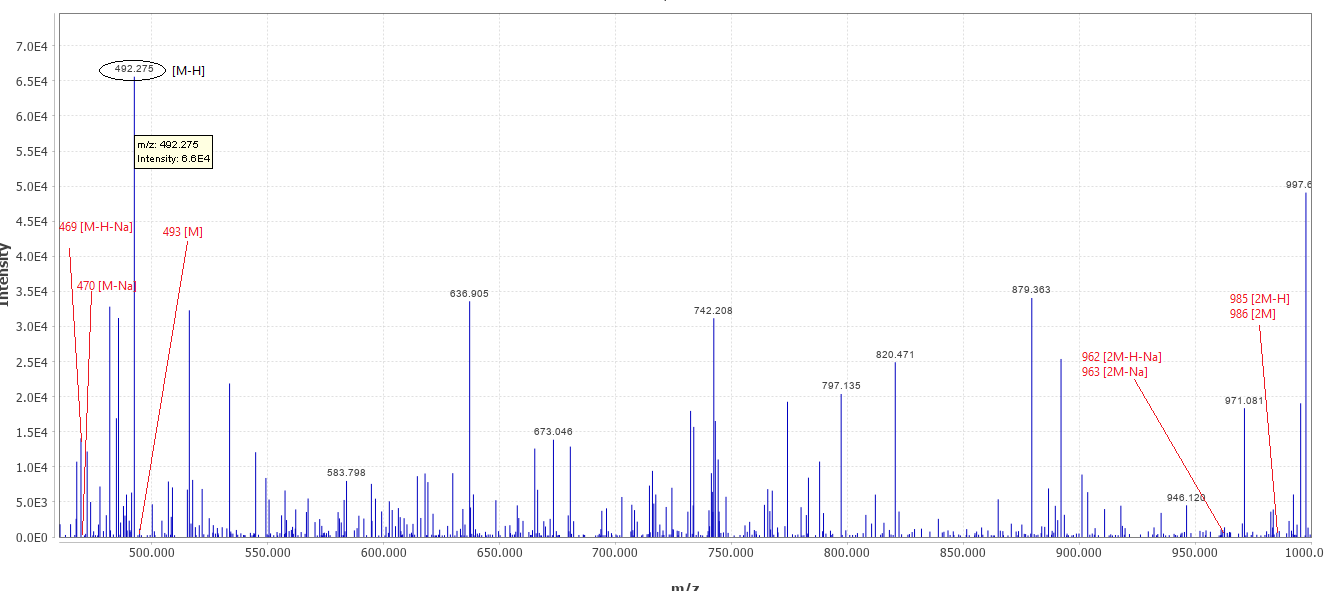

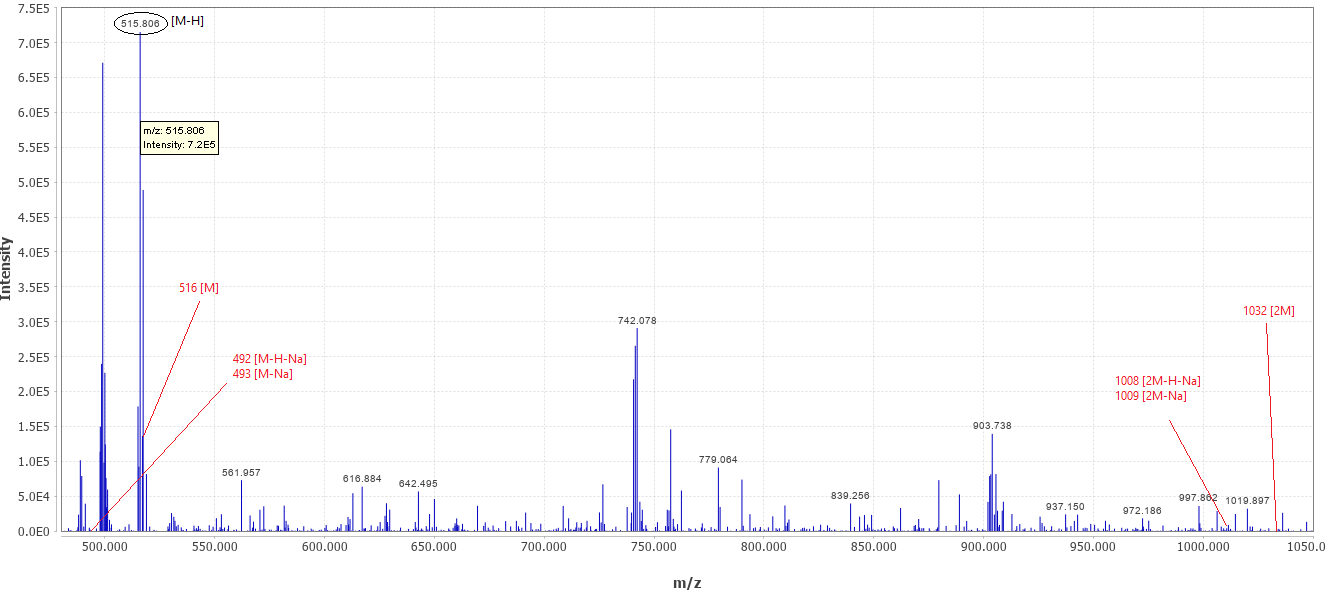

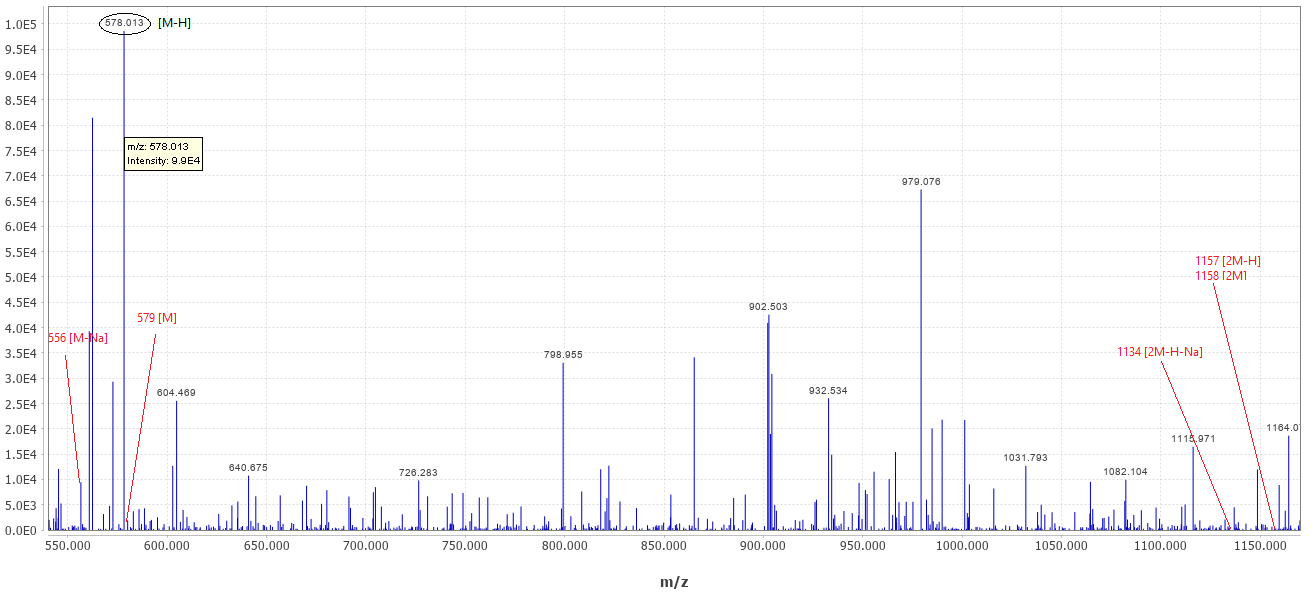

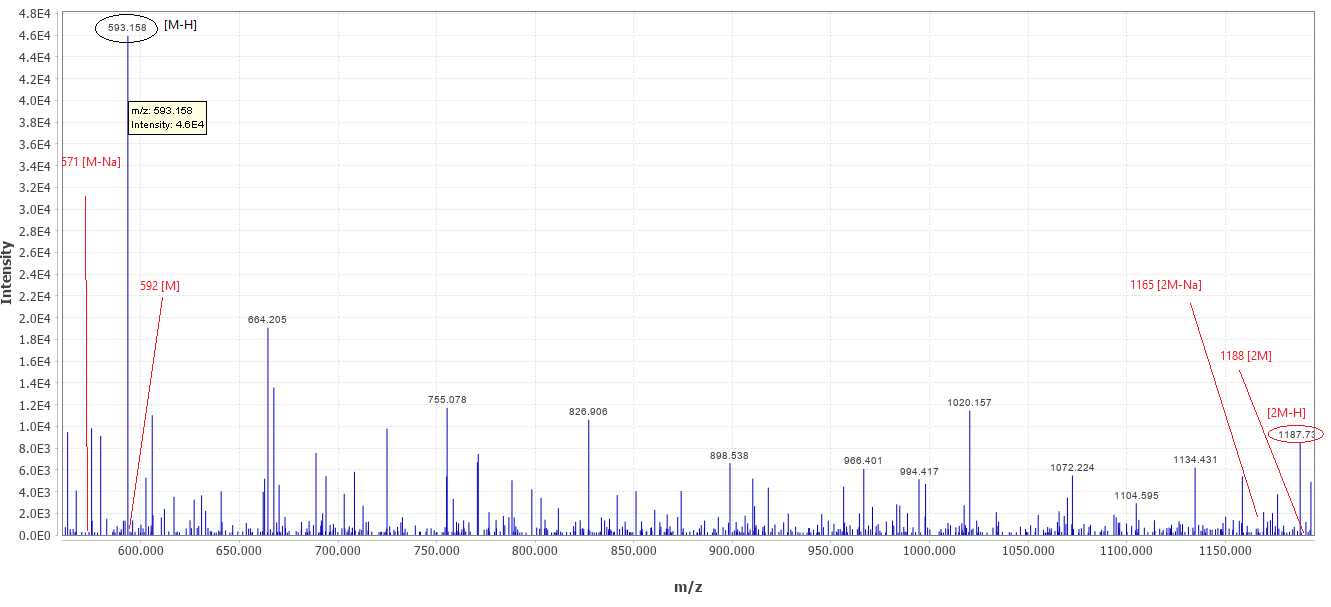

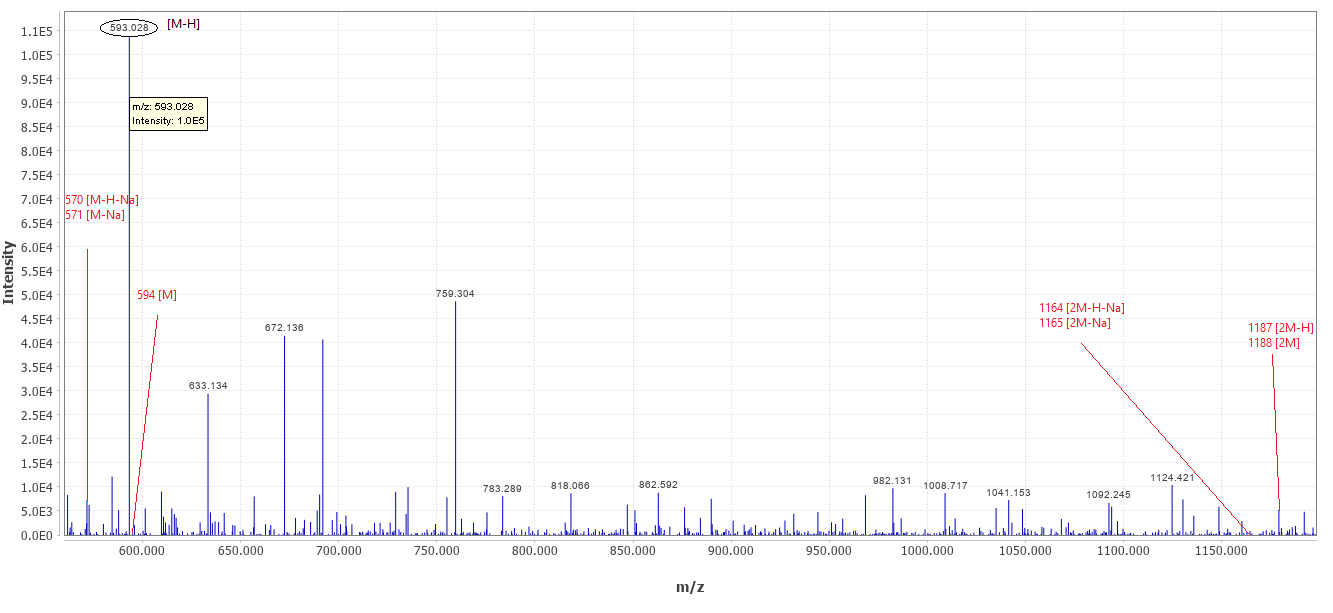

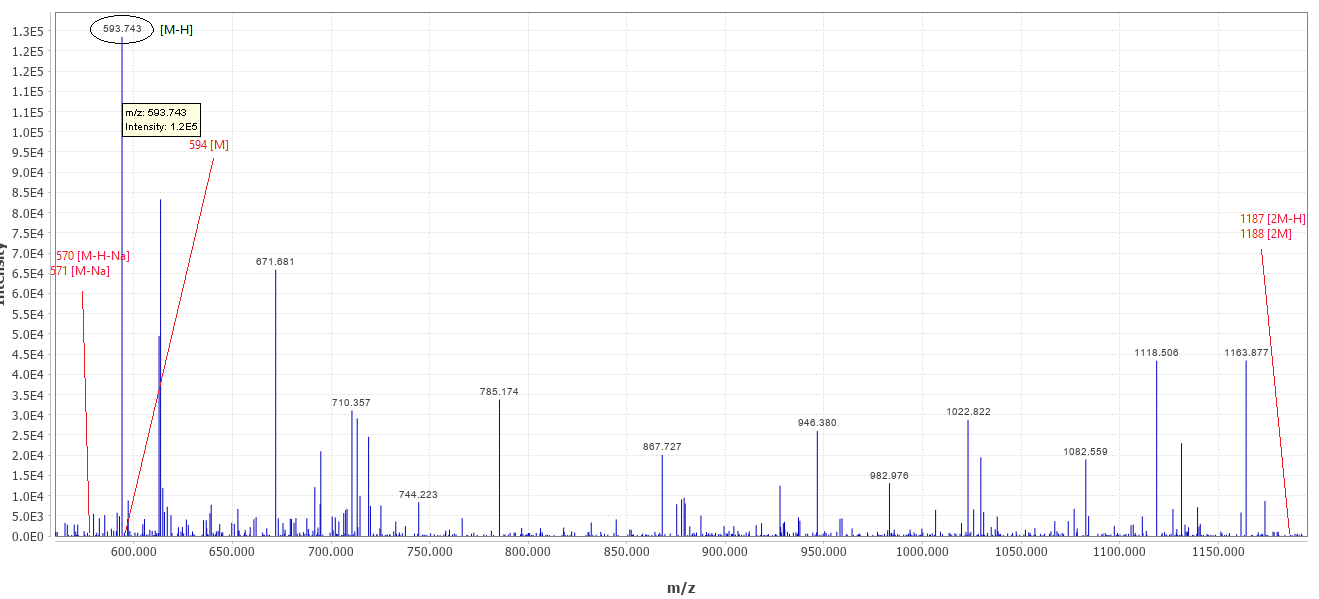

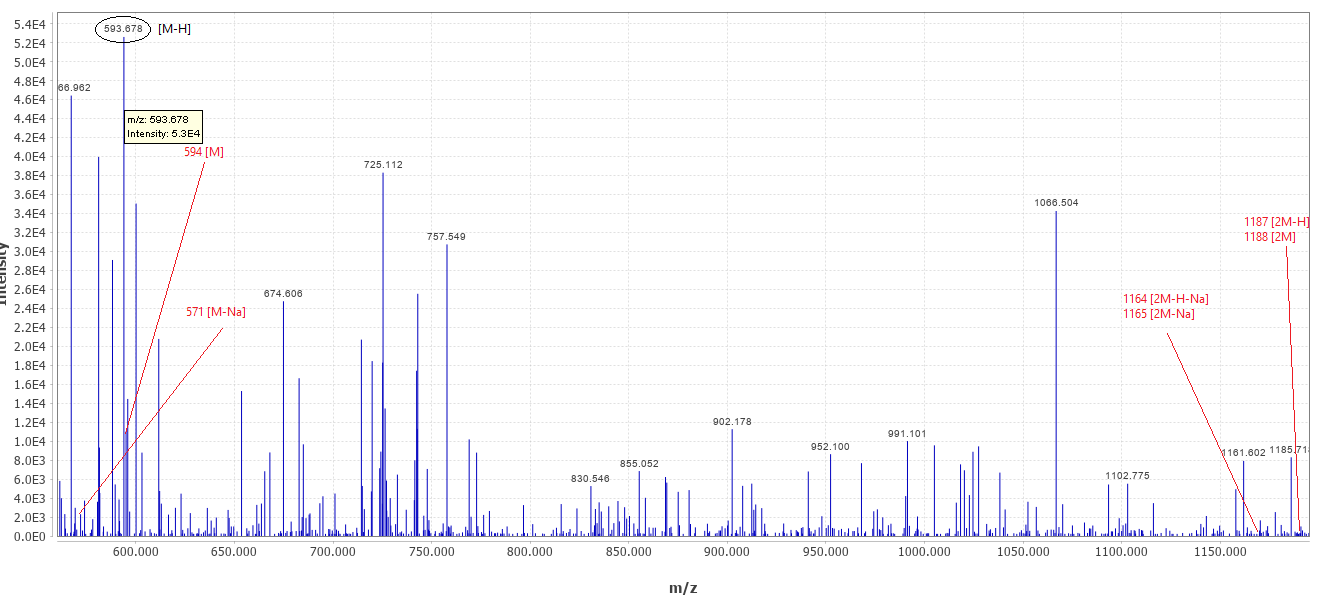

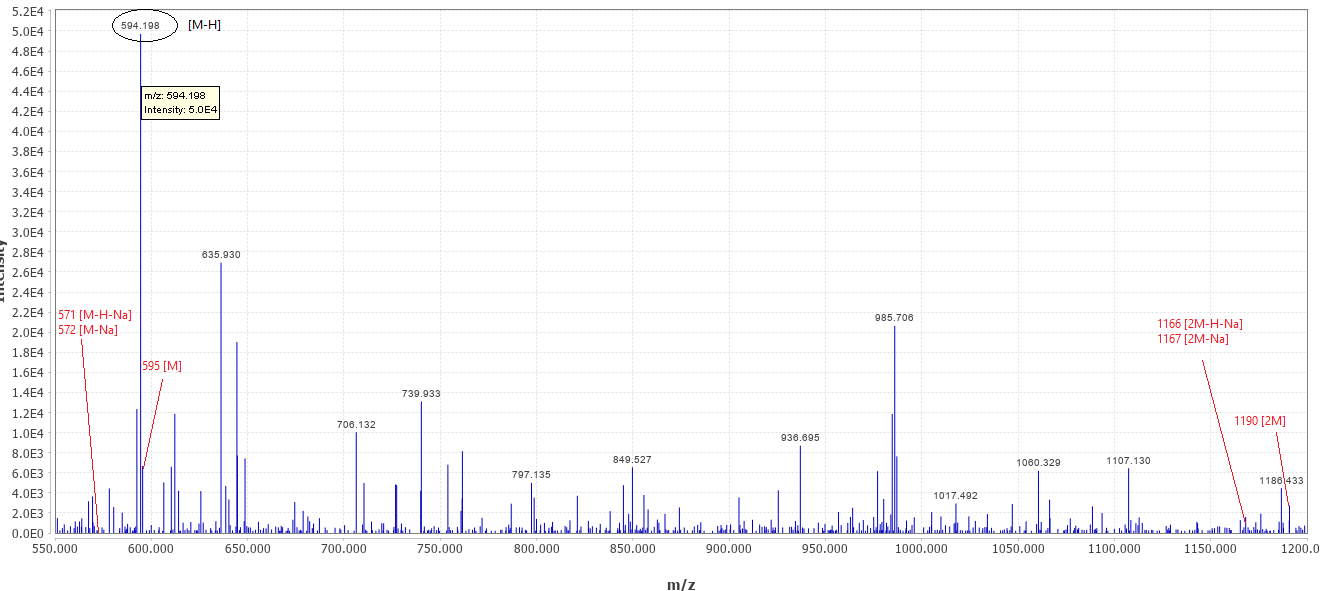

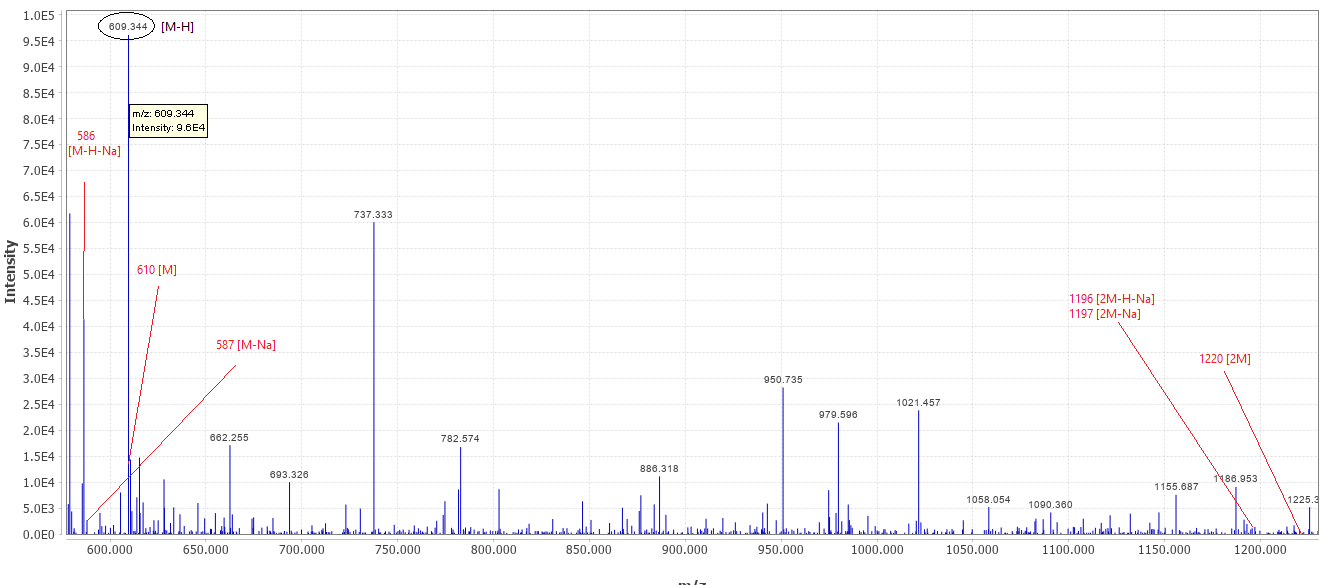

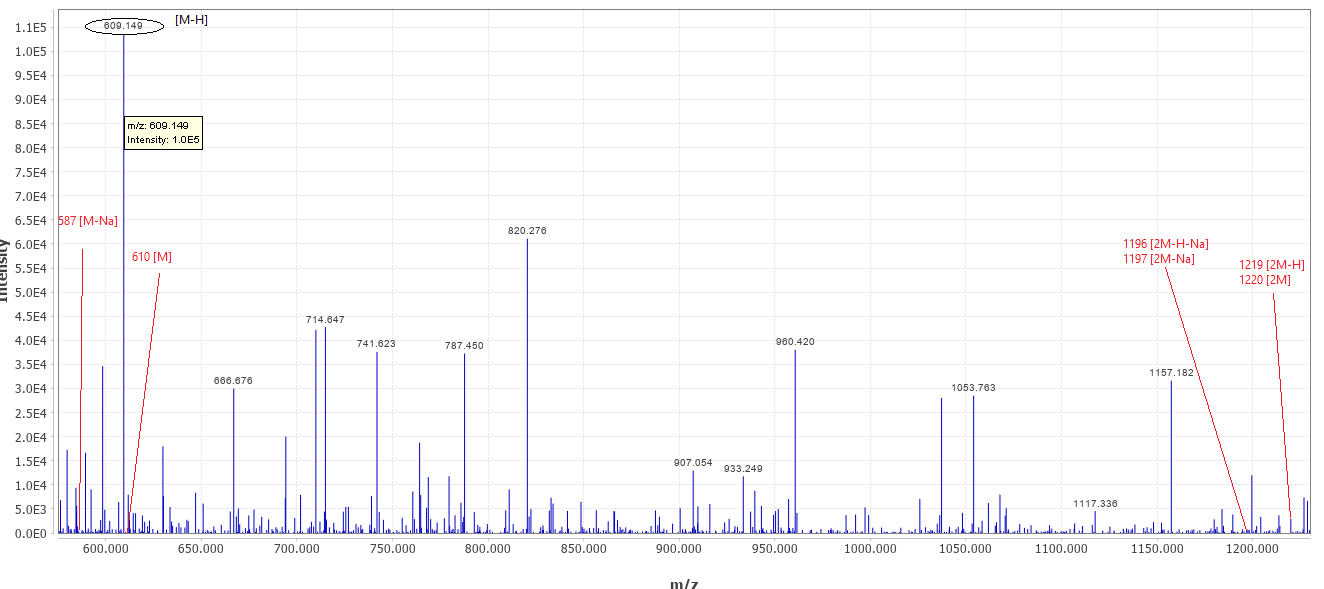

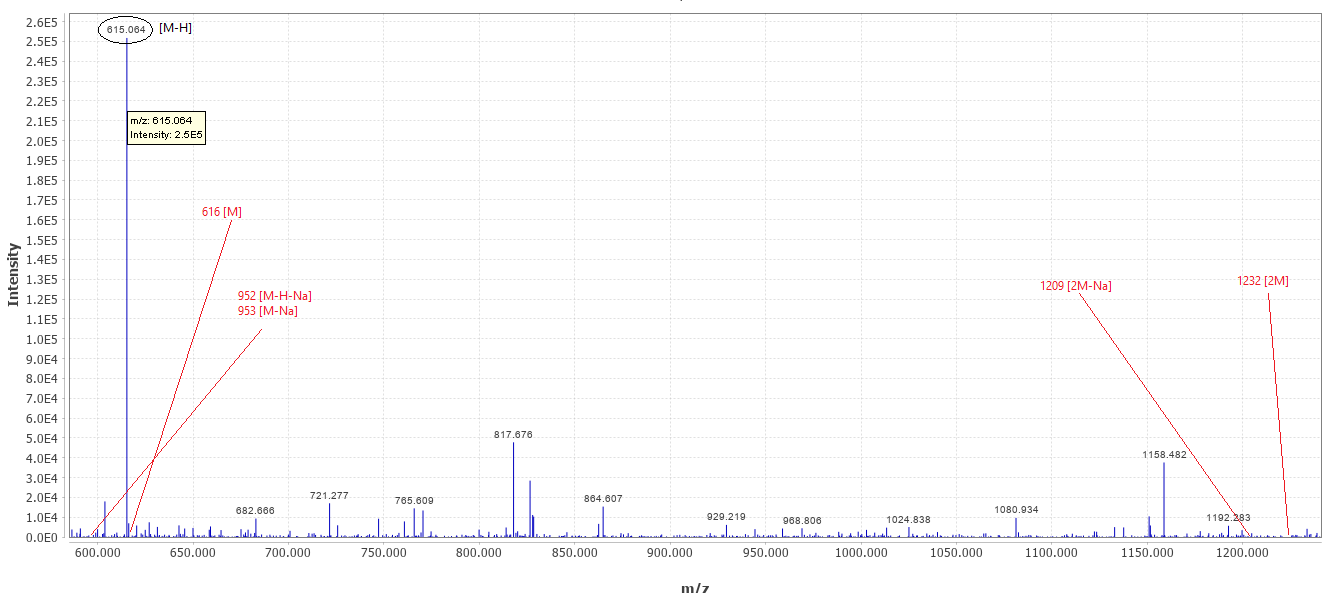

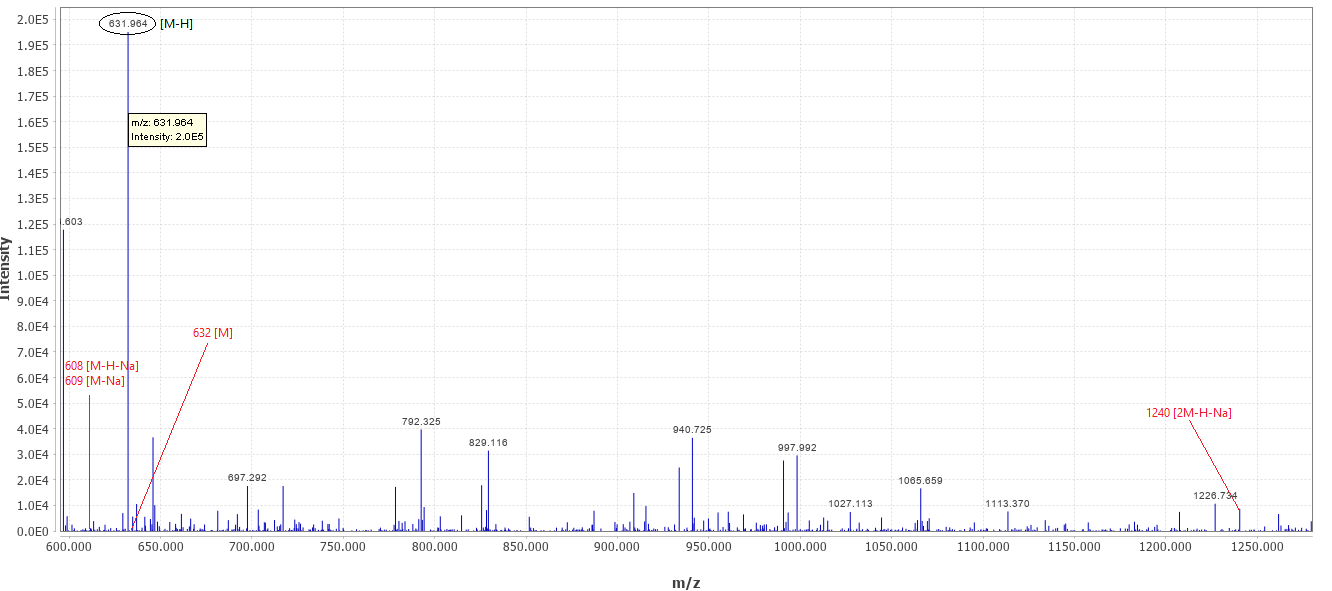

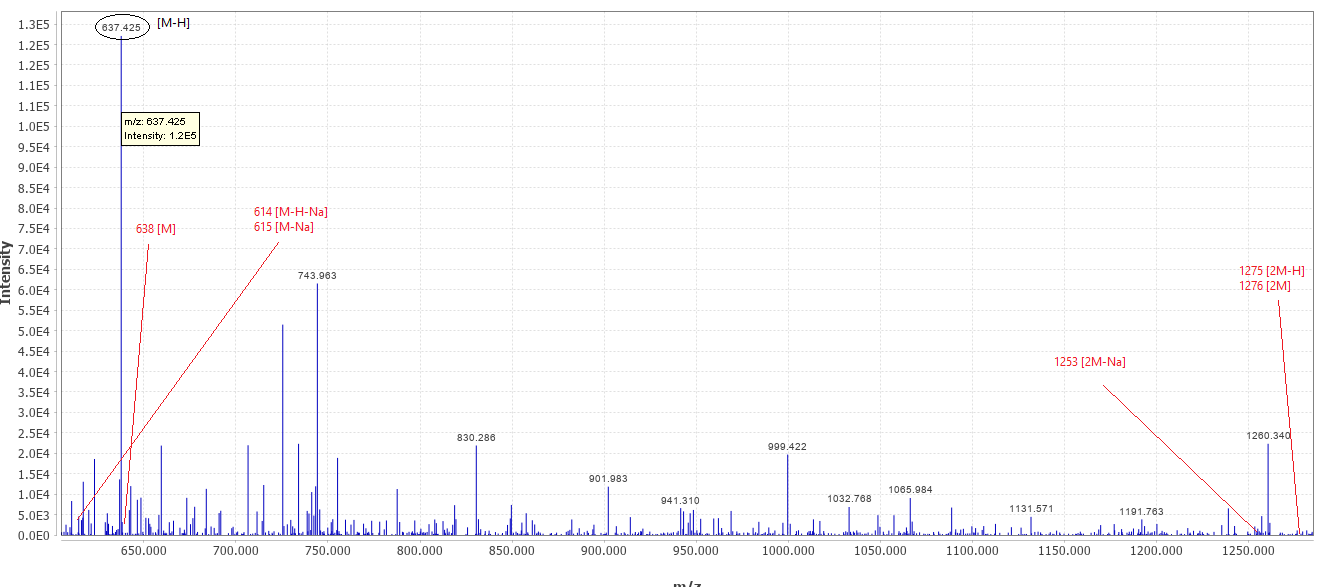

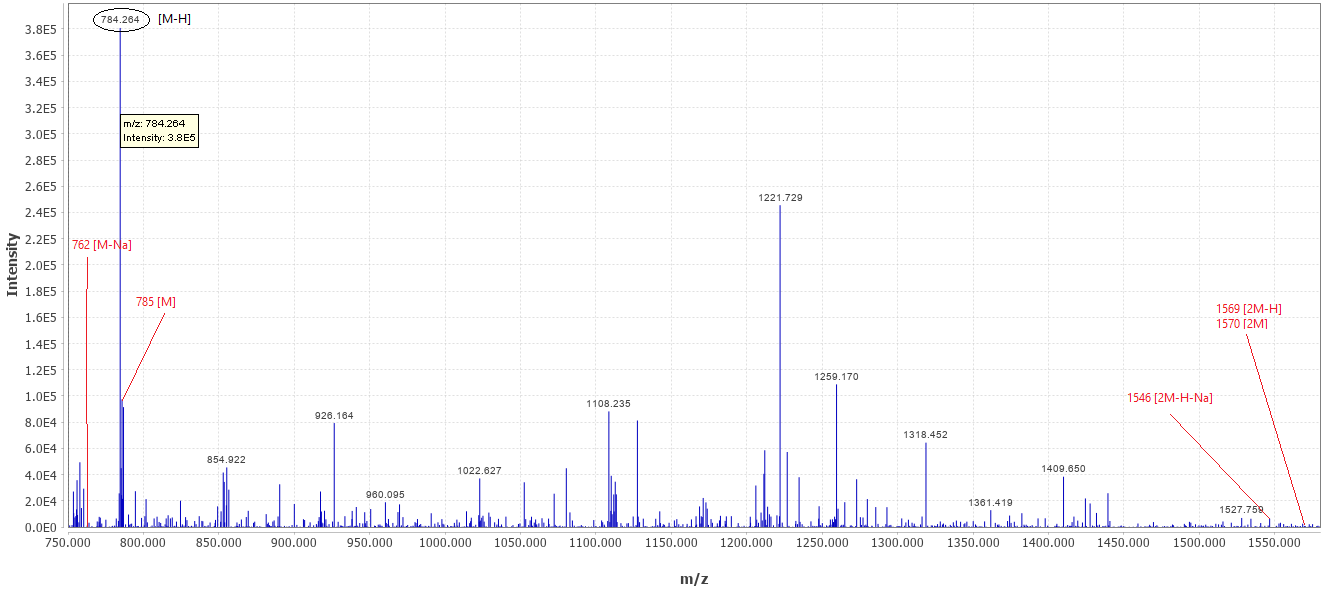

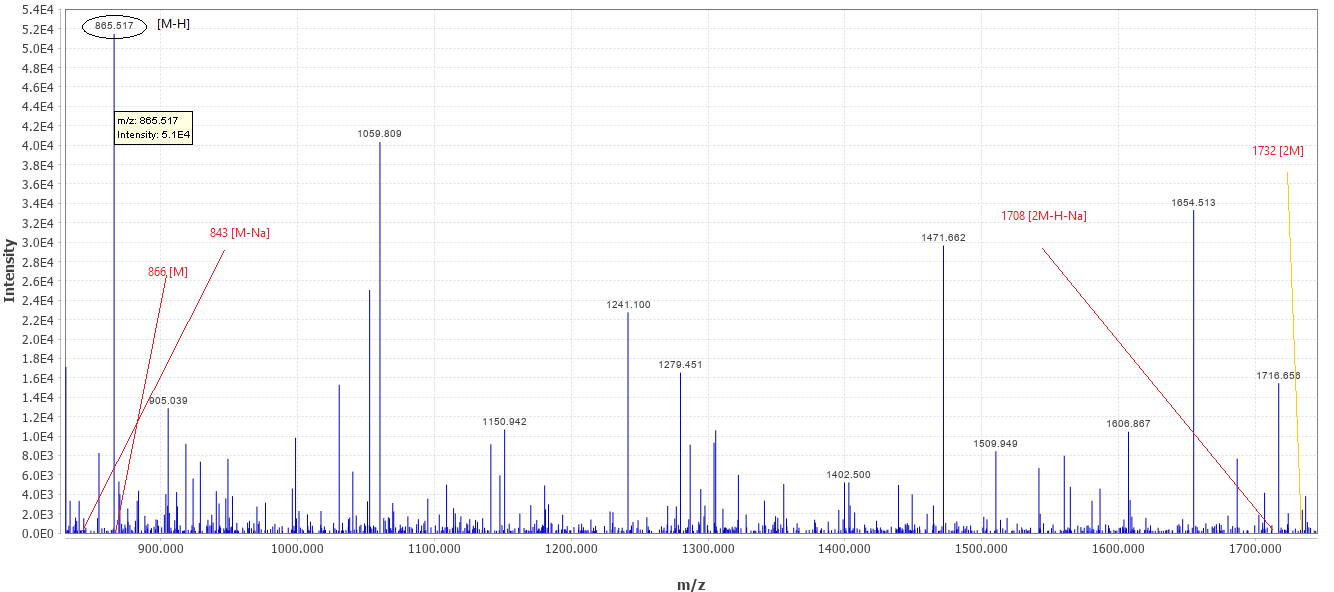

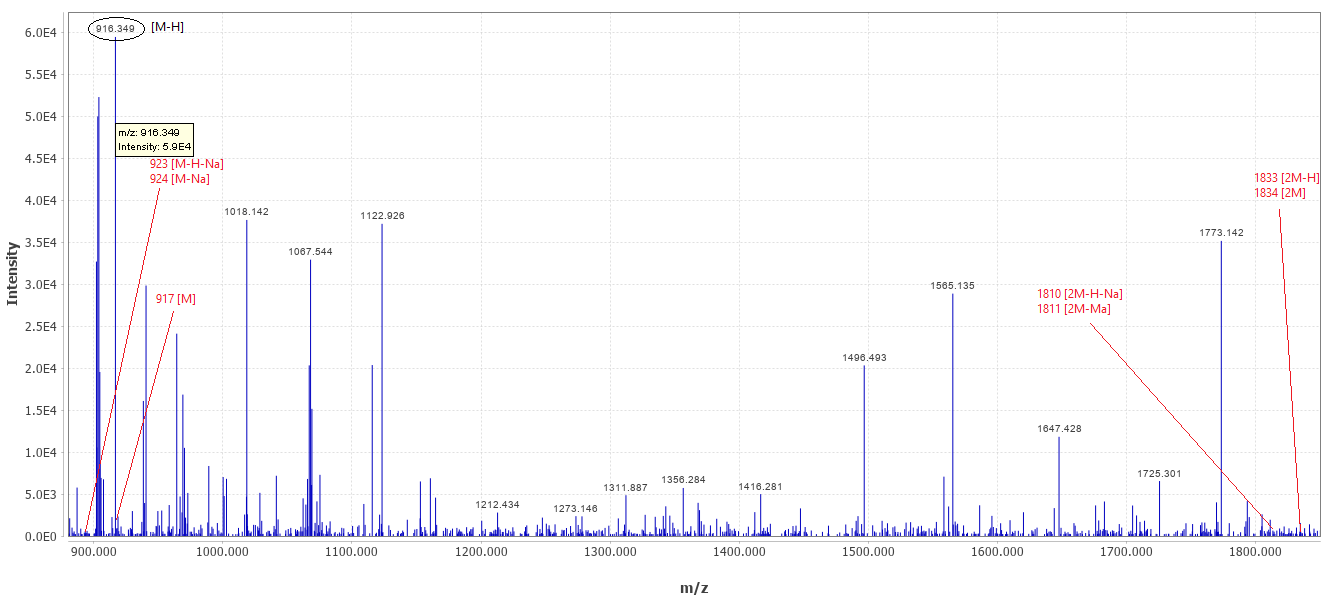

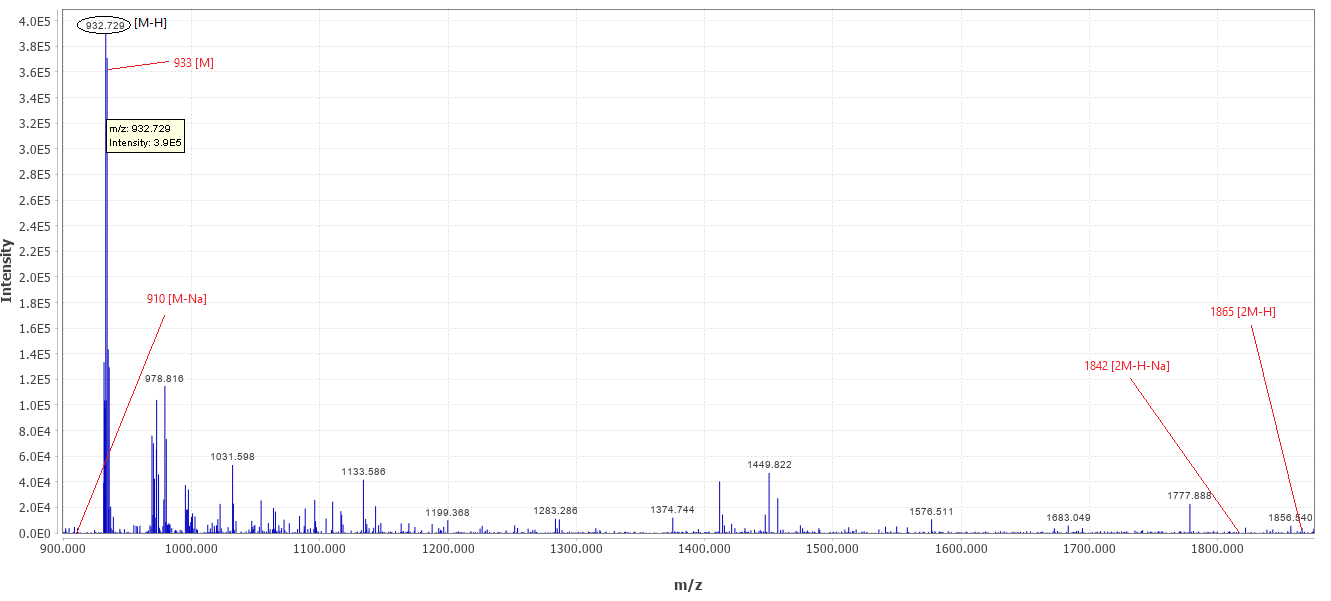

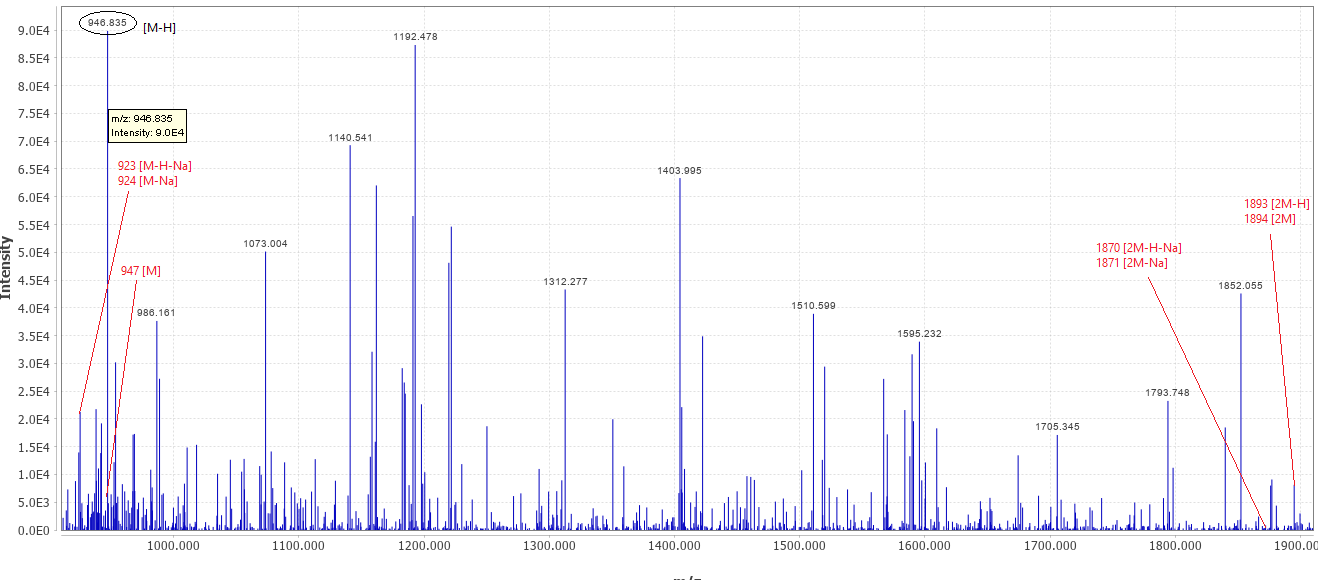


**Fig S3**. Mass spectra and corresponding adducts in all identified compounds of hydro-methanolic extract of *C.pentagyna* fruit.


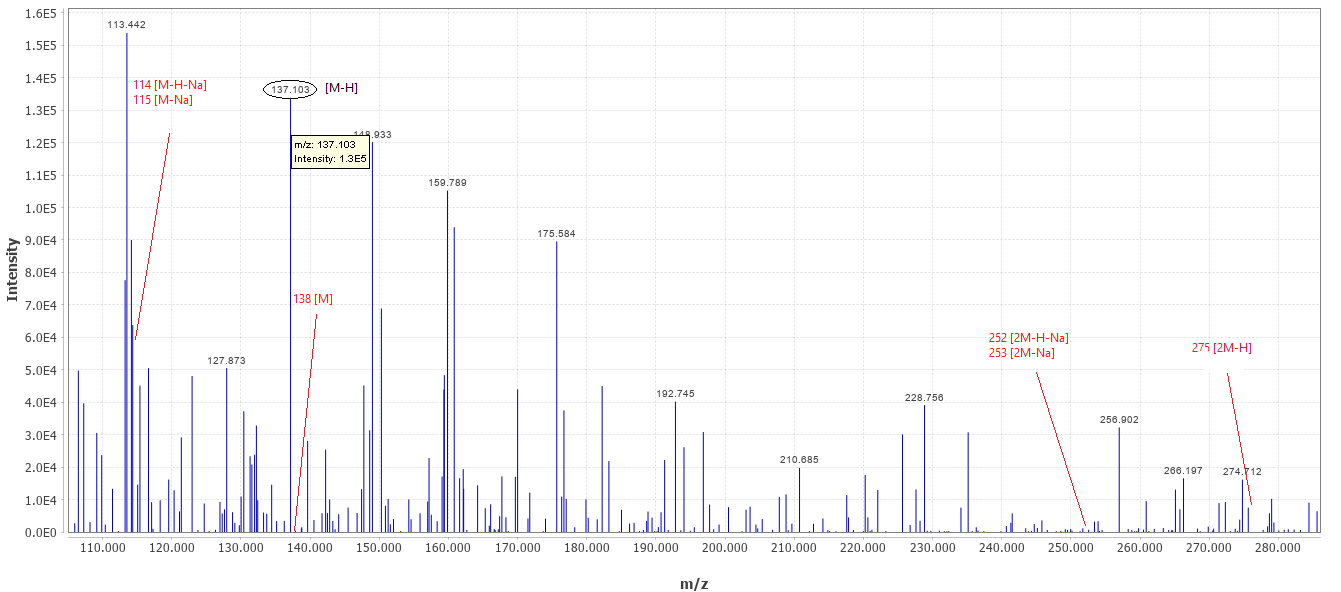

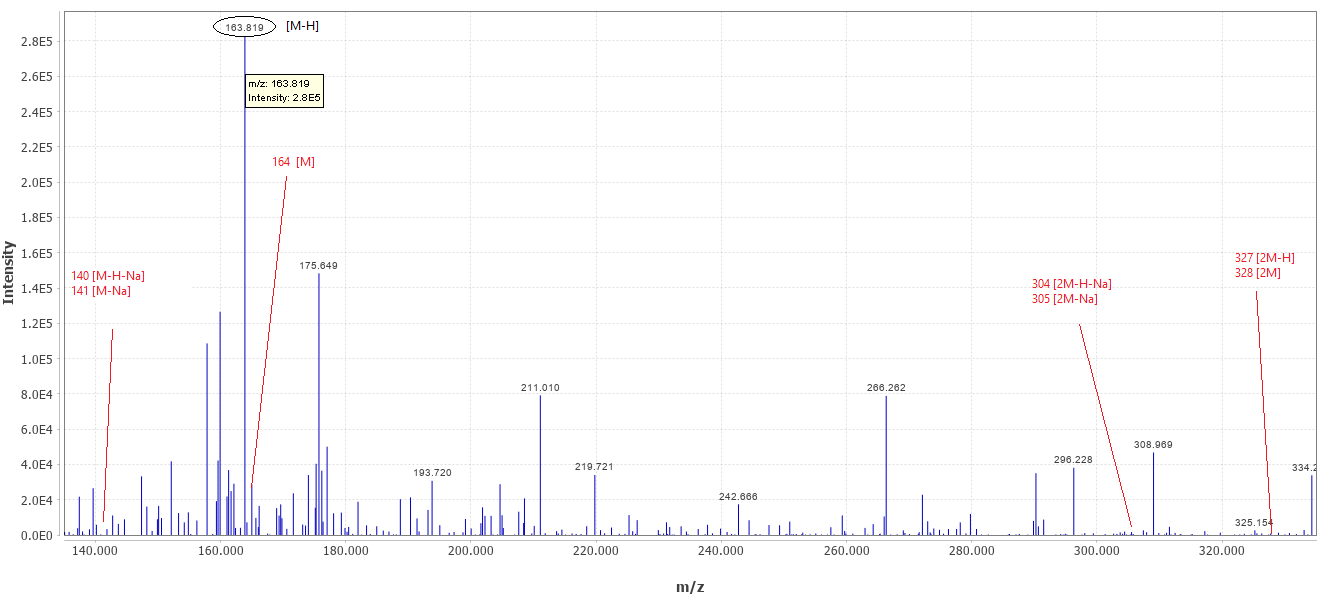

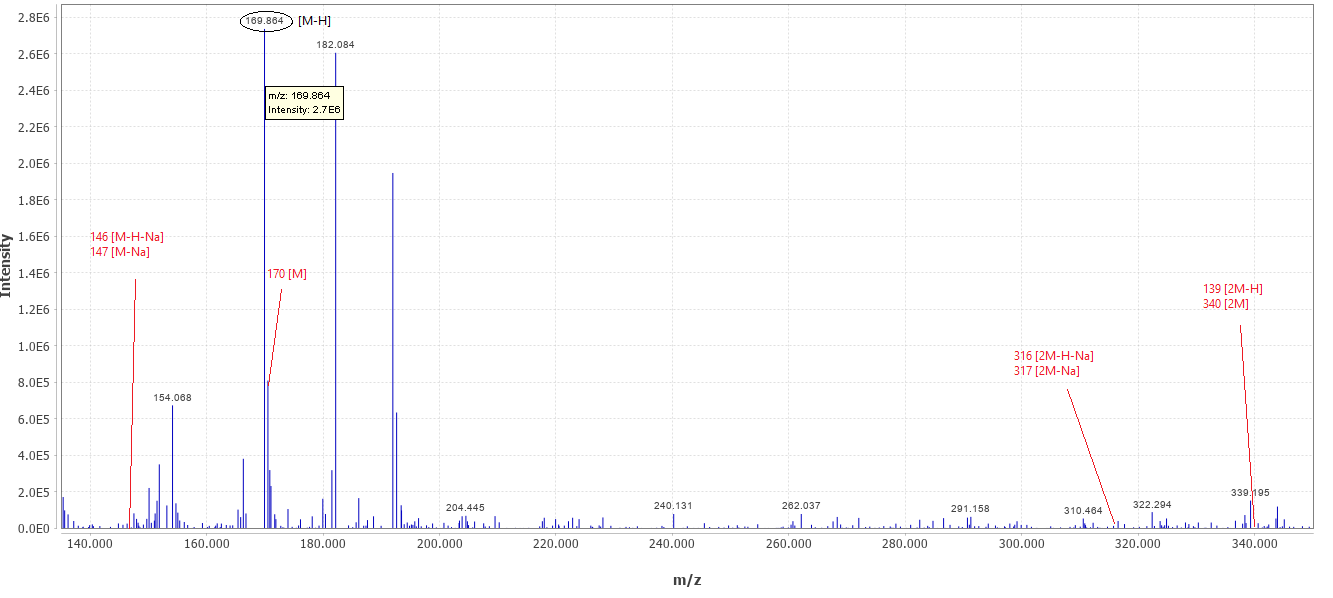

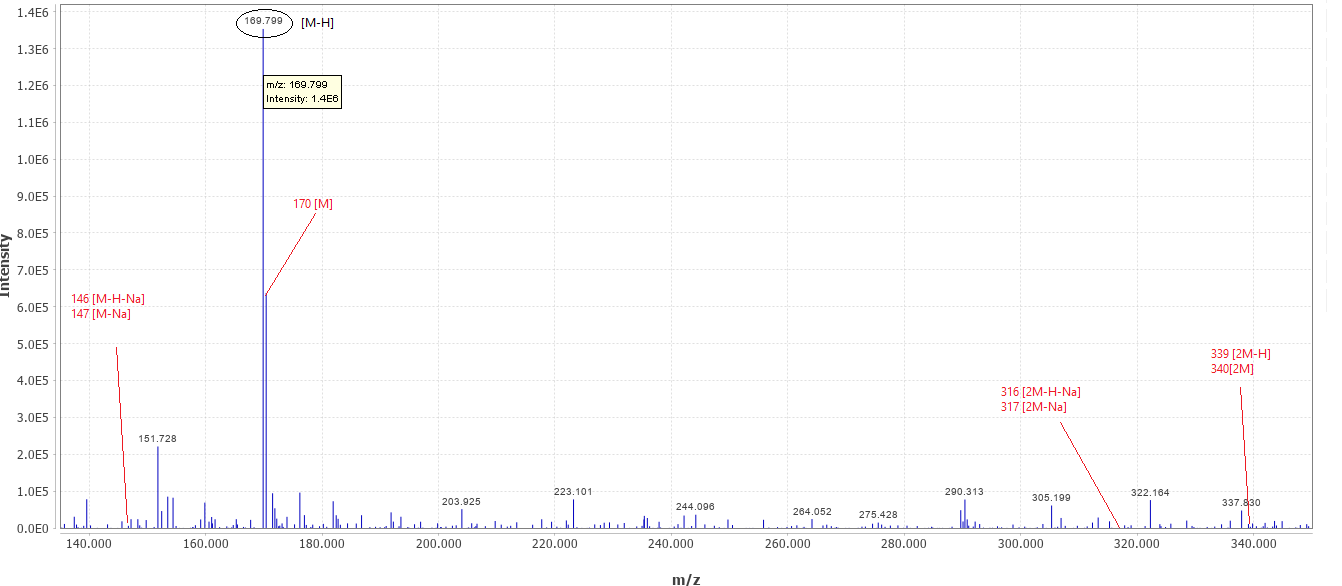

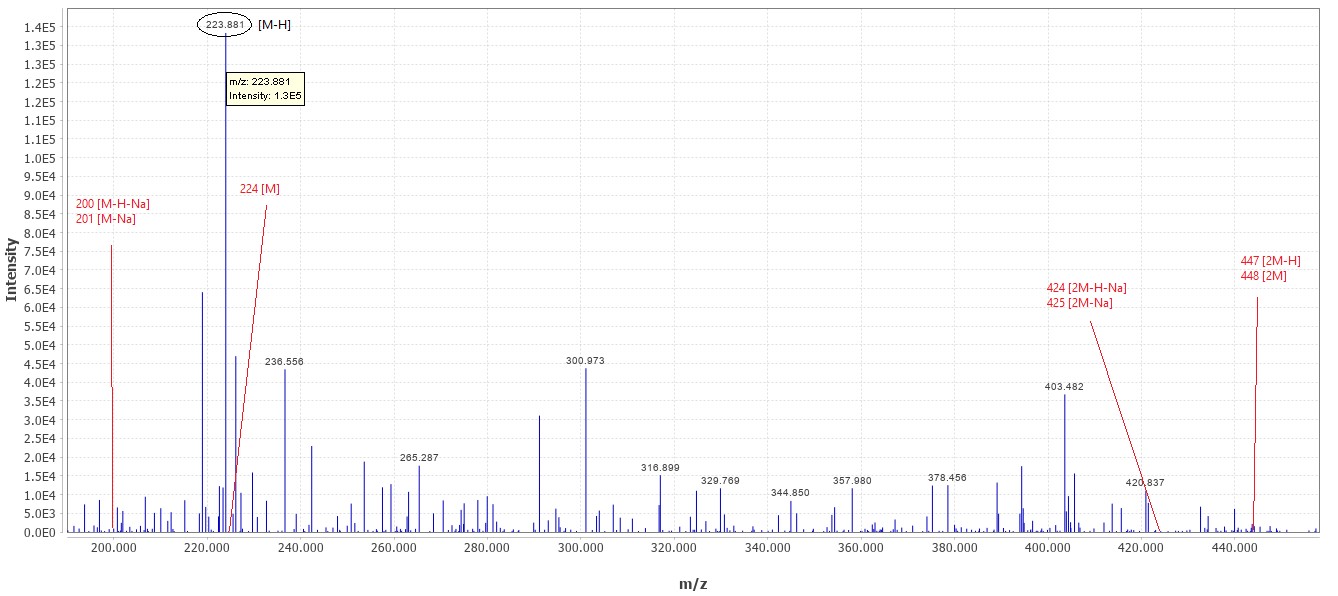

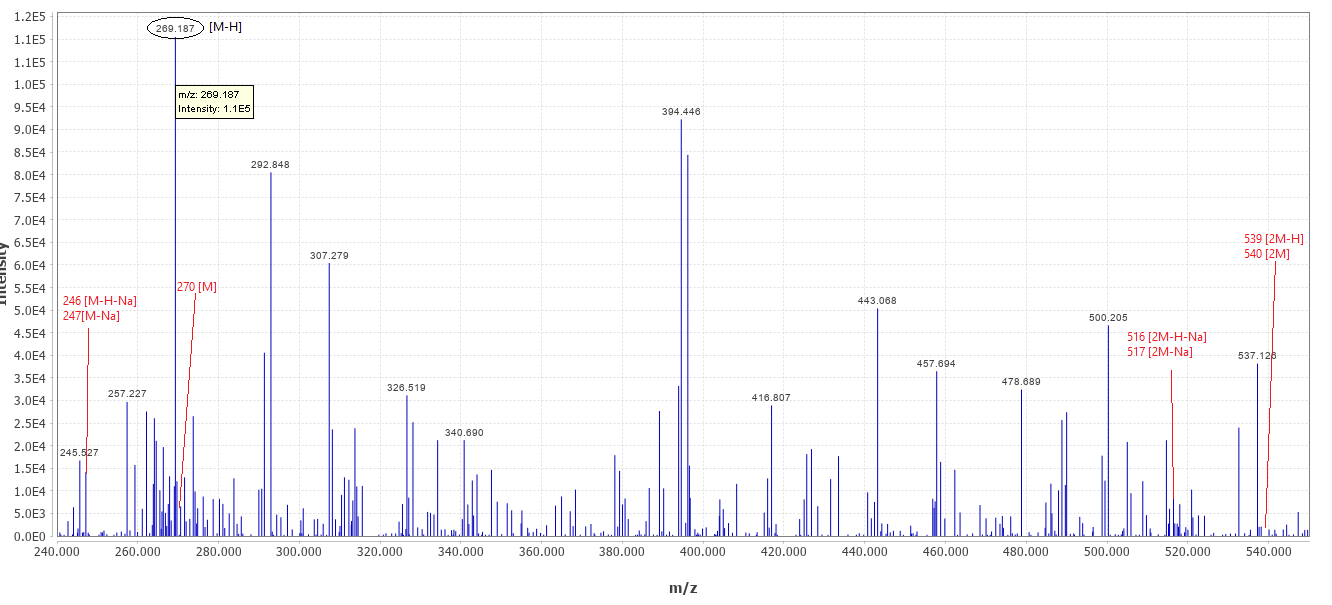

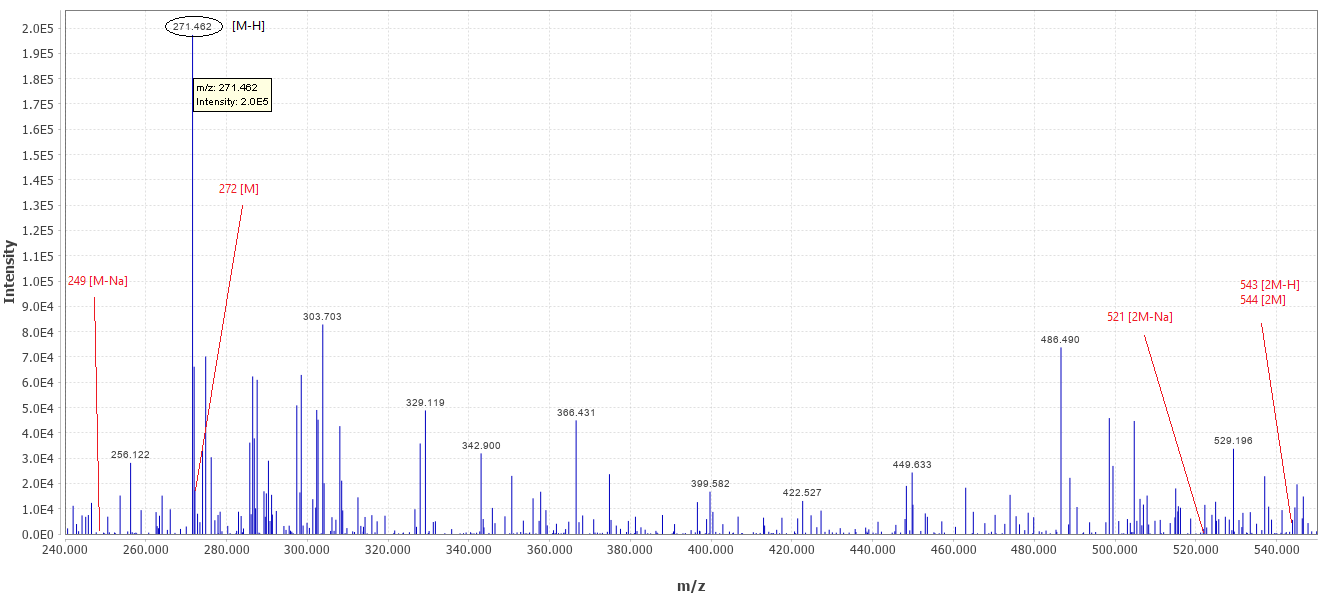

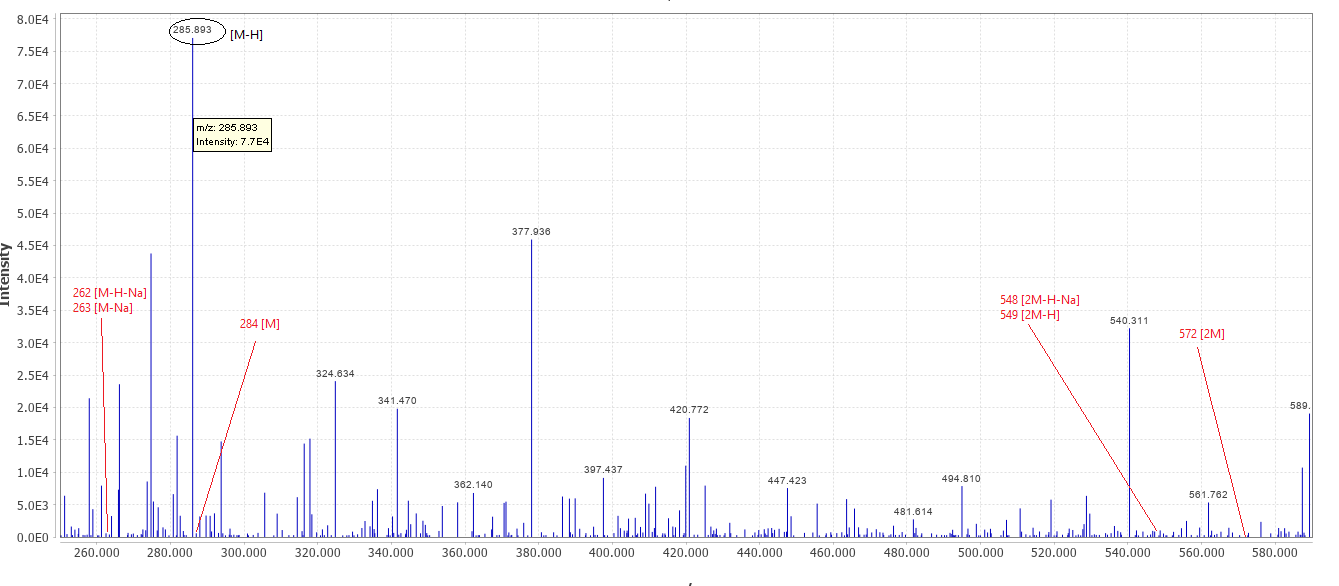

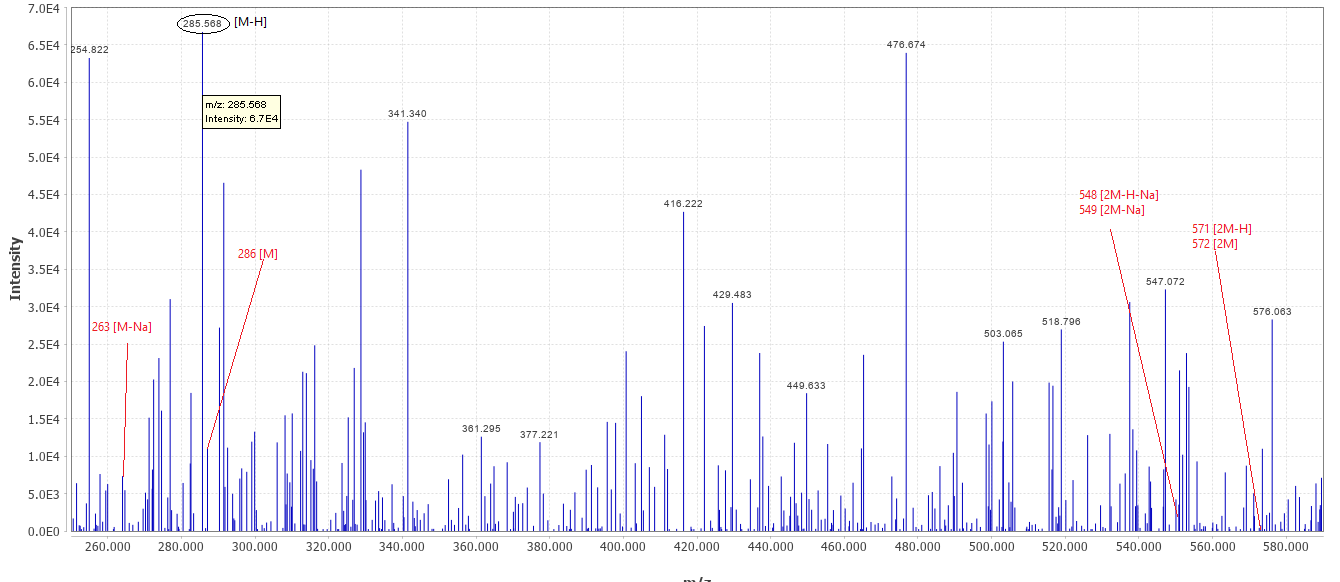

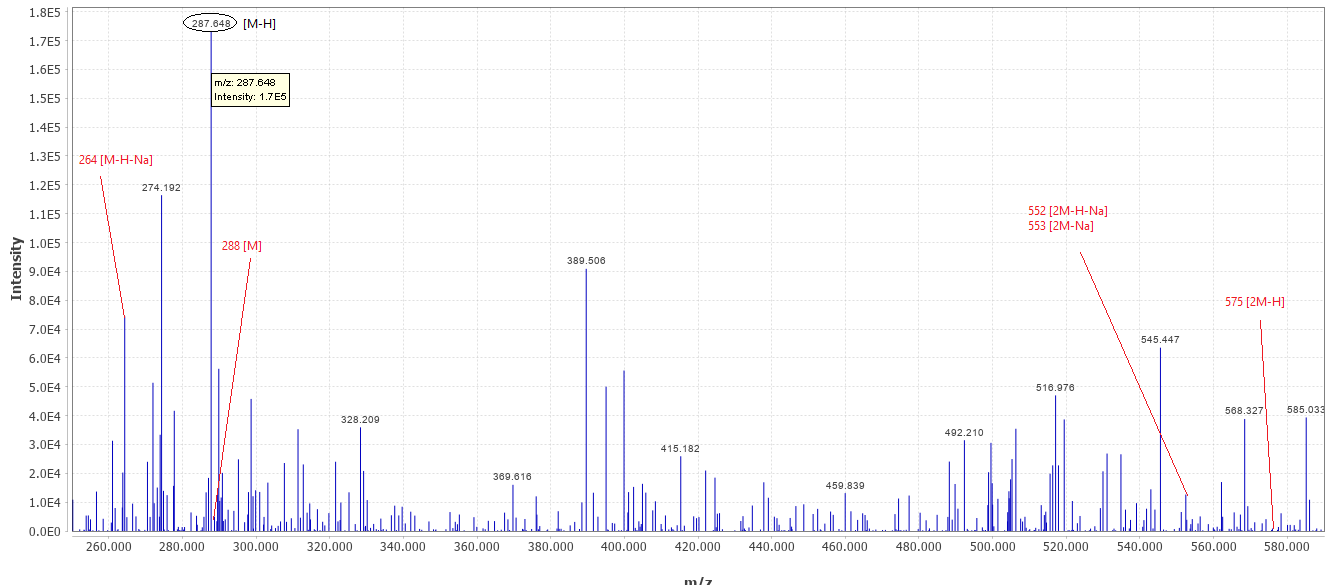

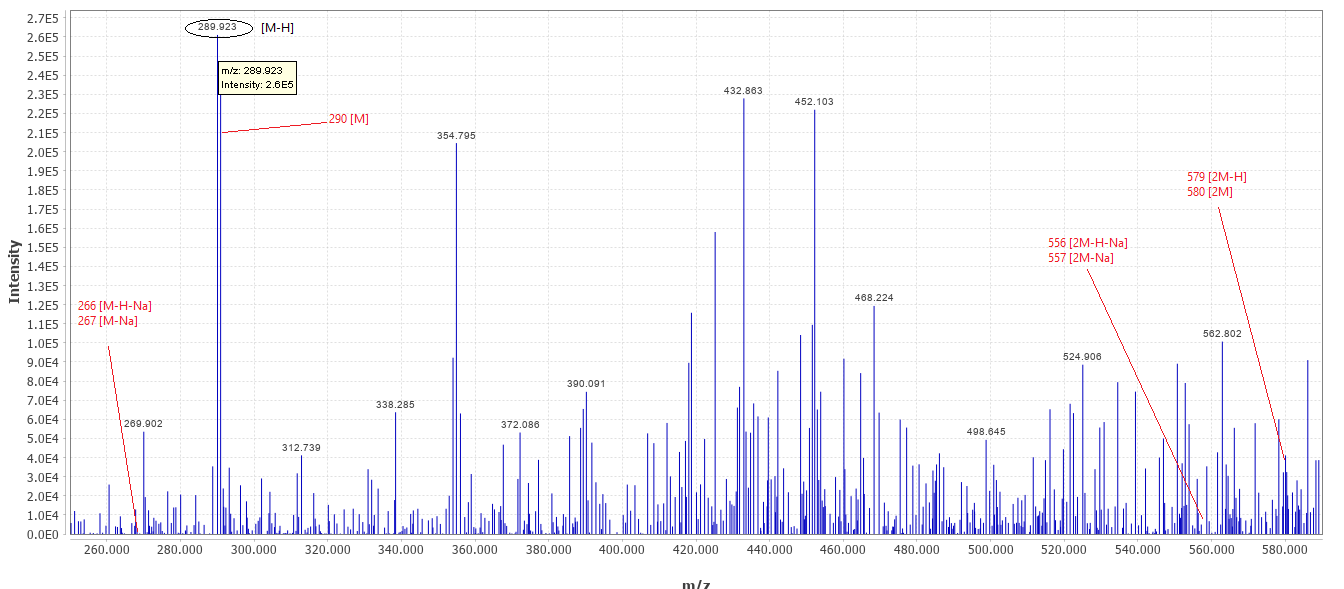

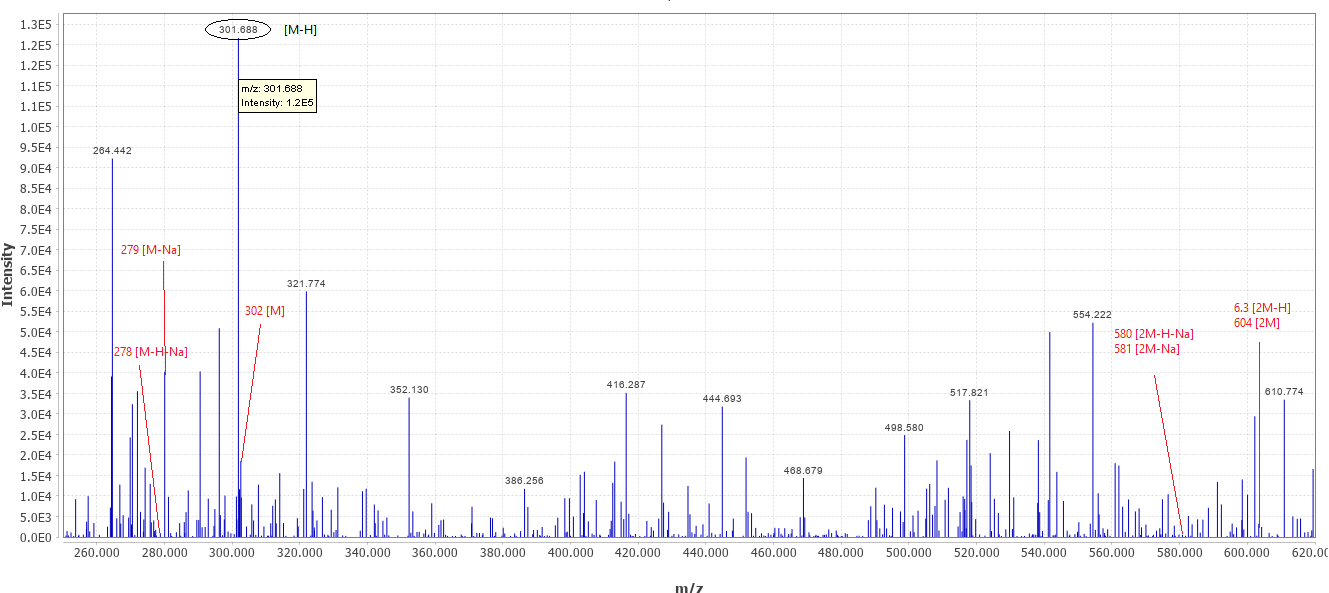

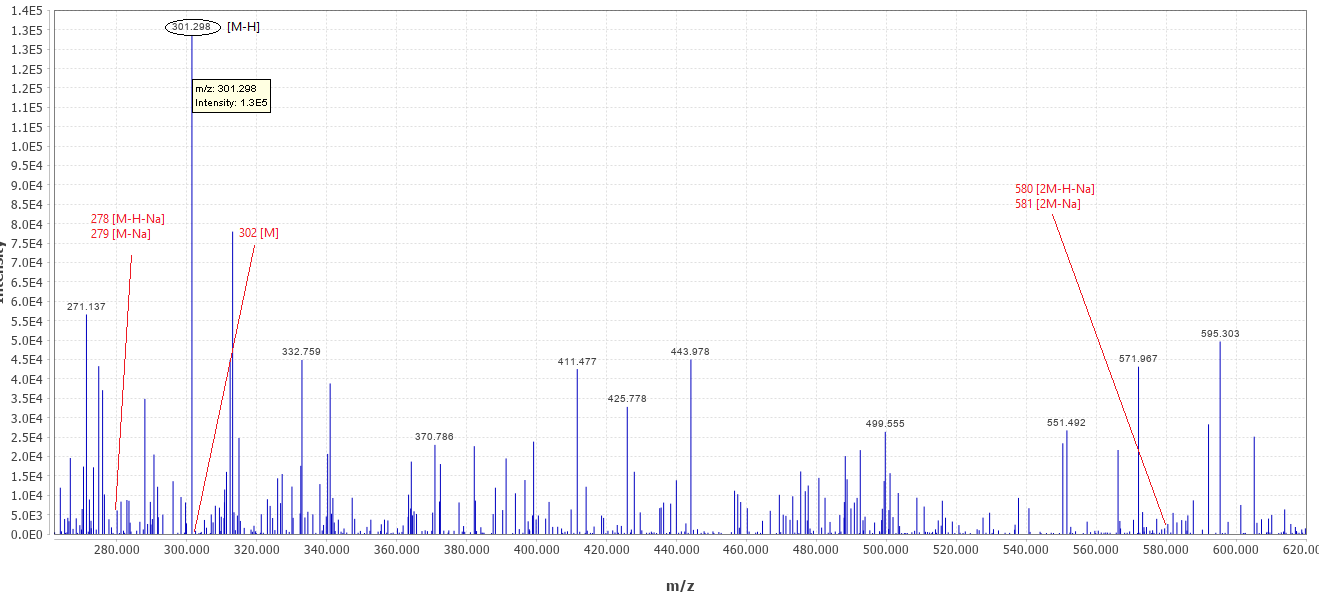

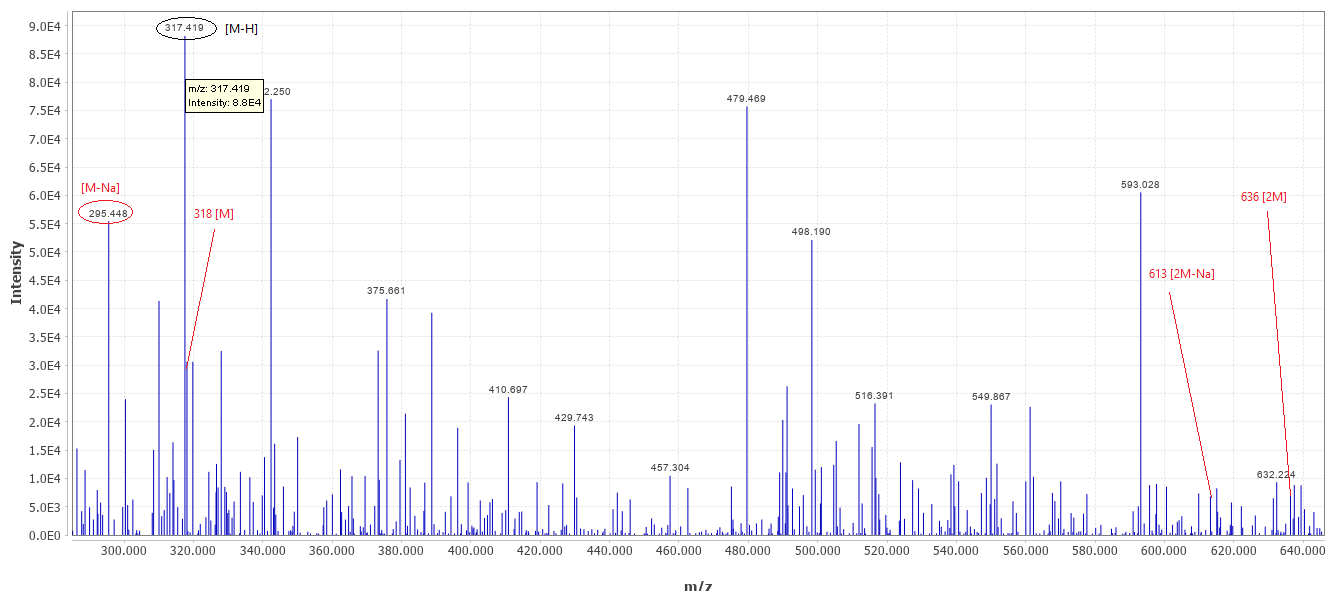

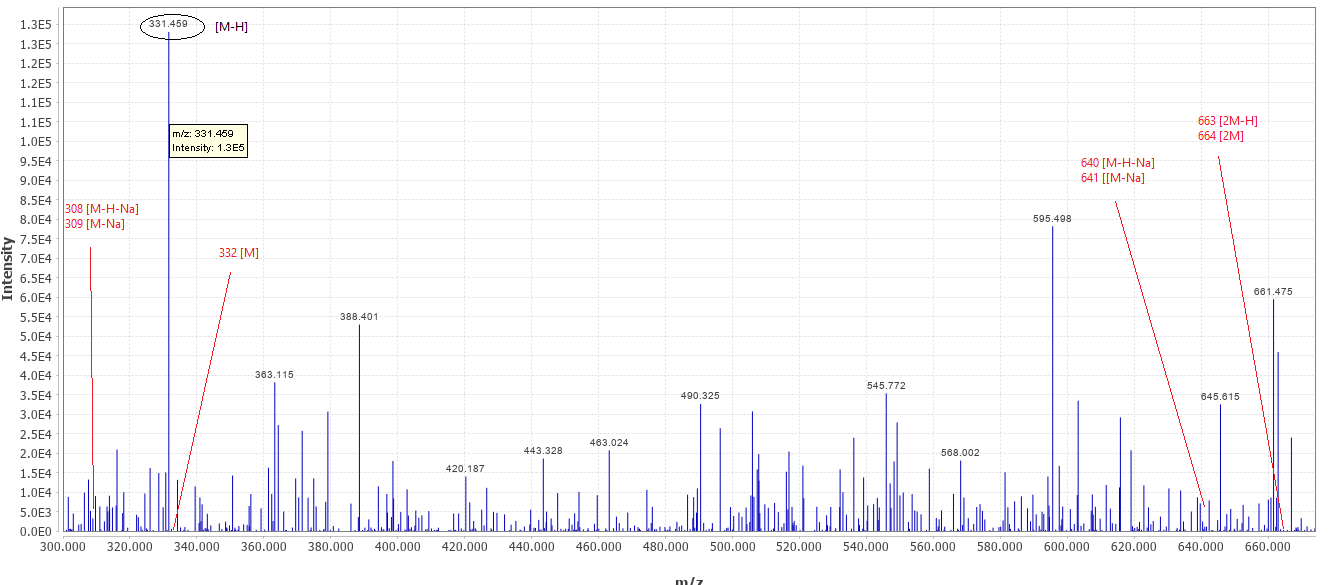

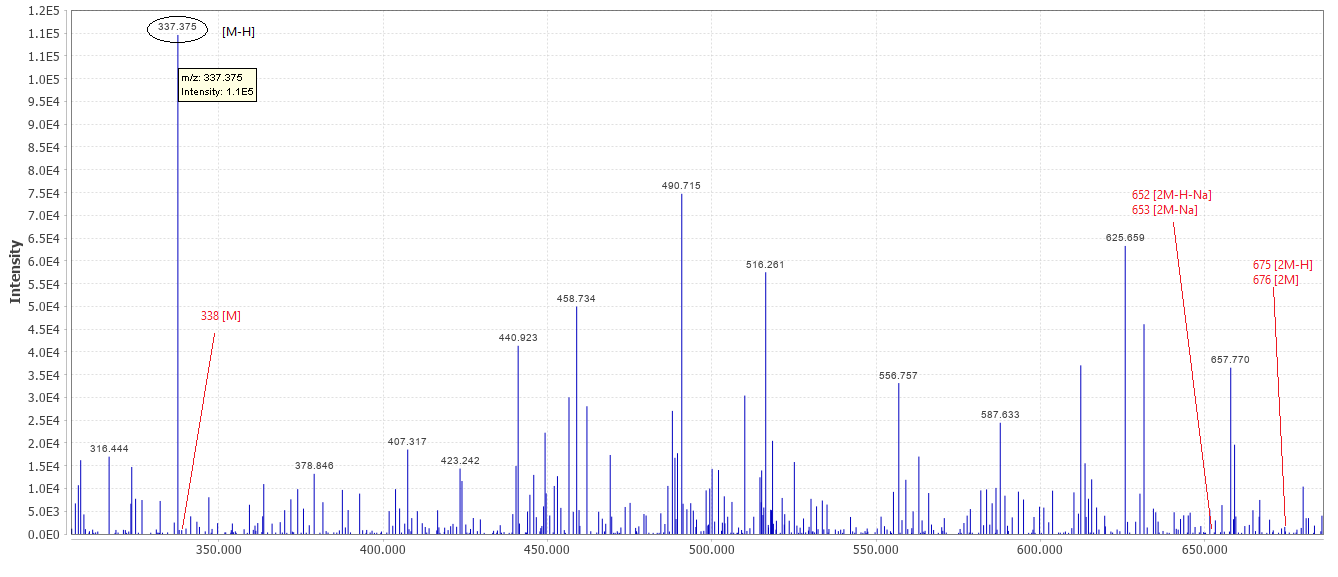

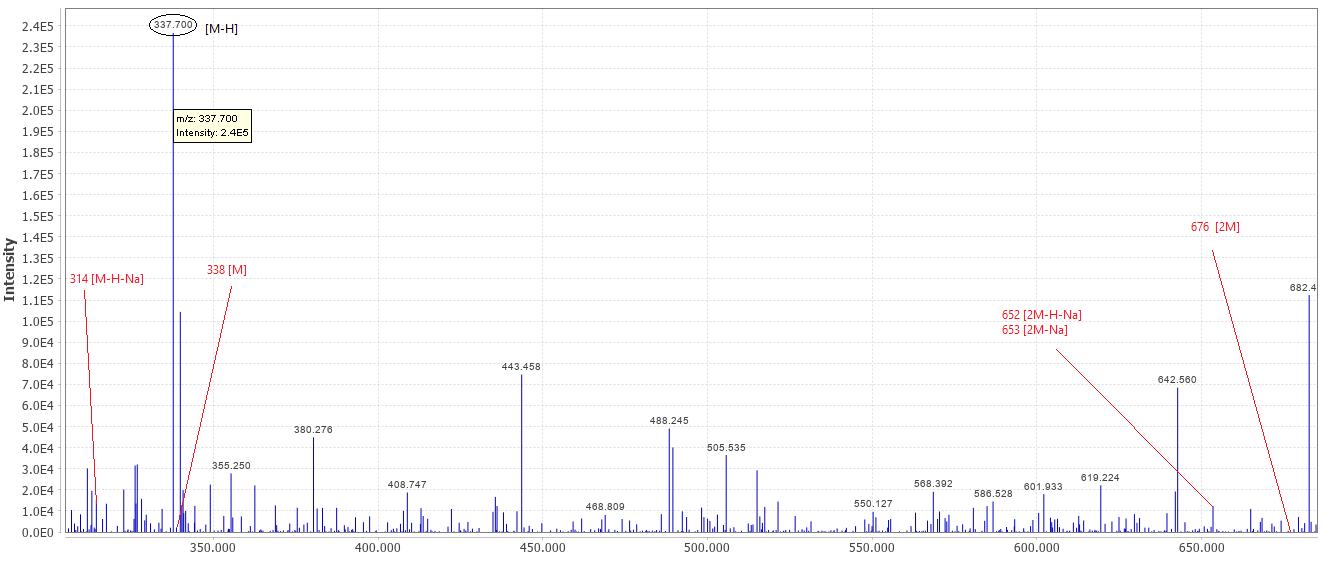

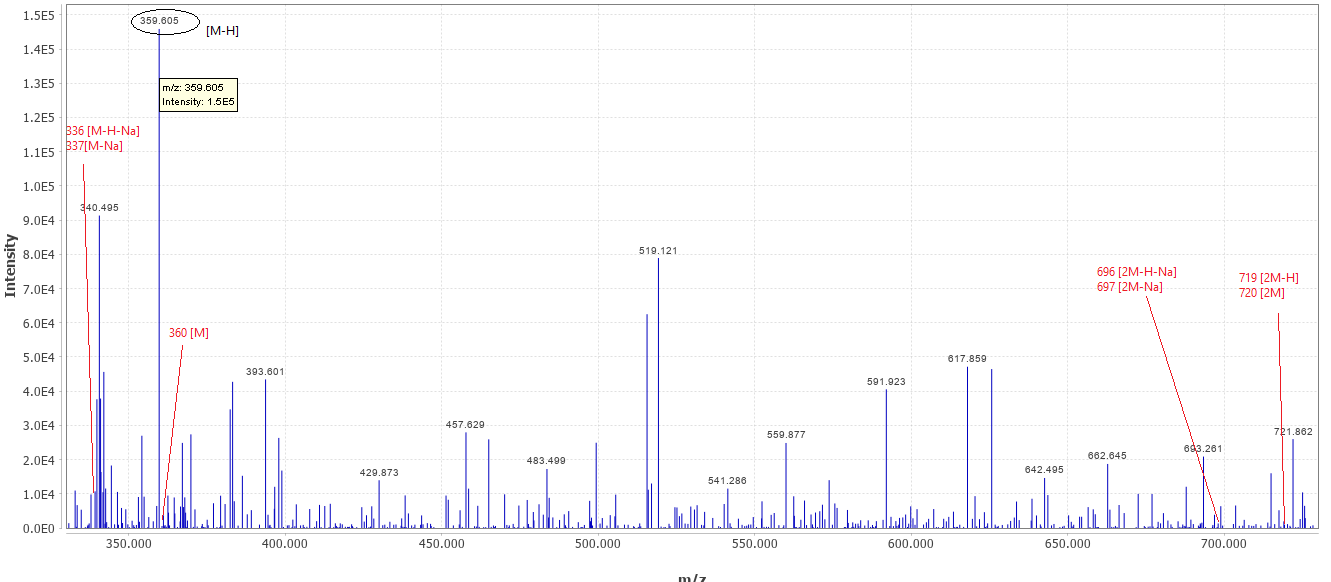

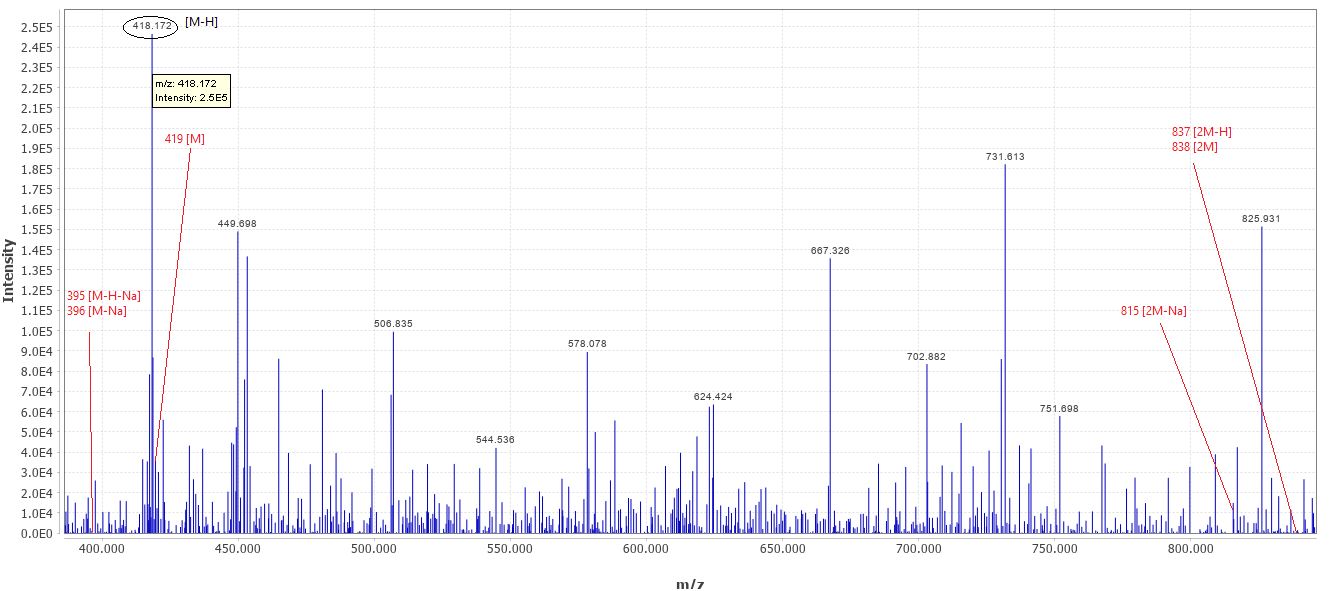

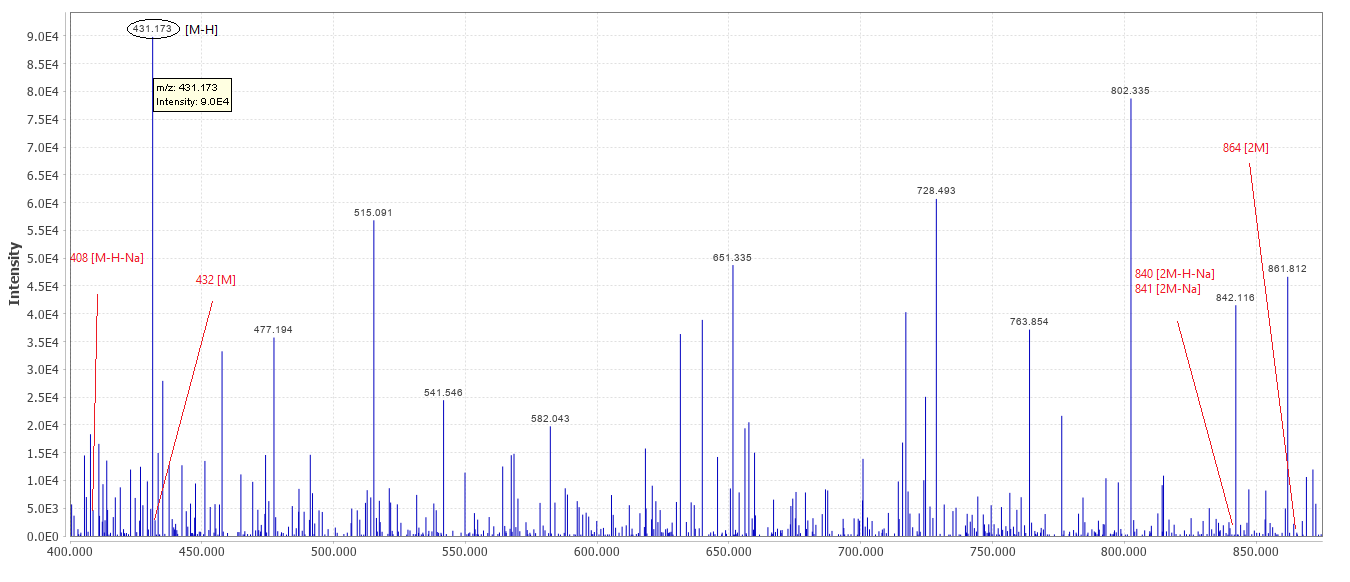

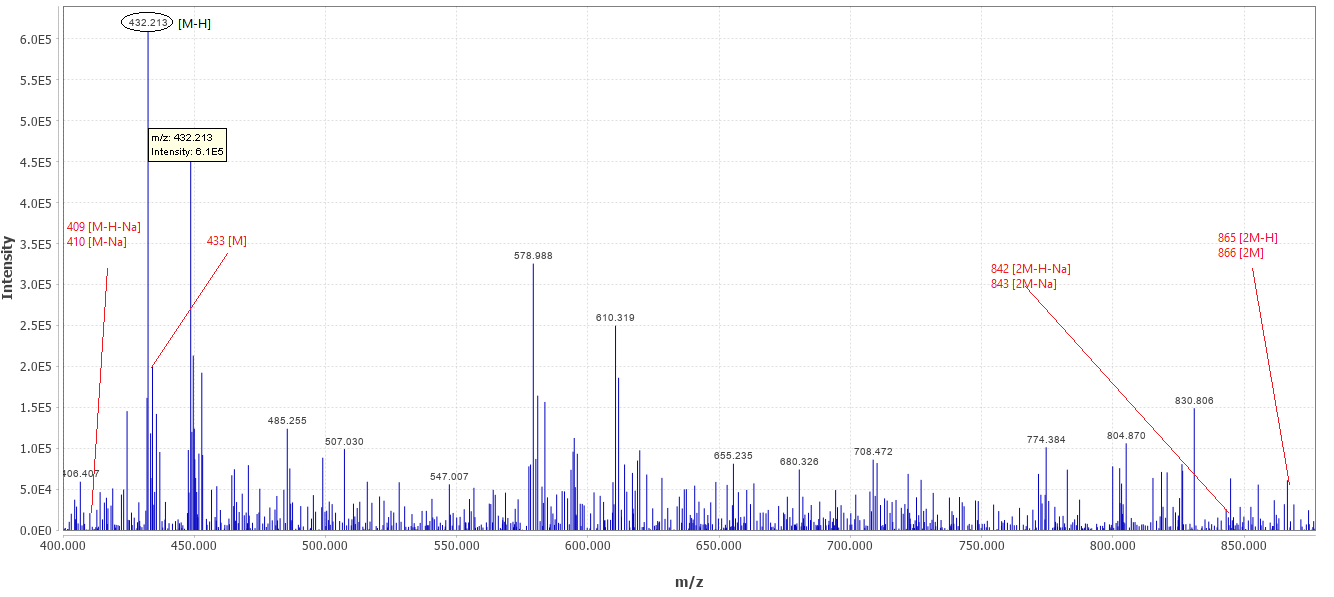

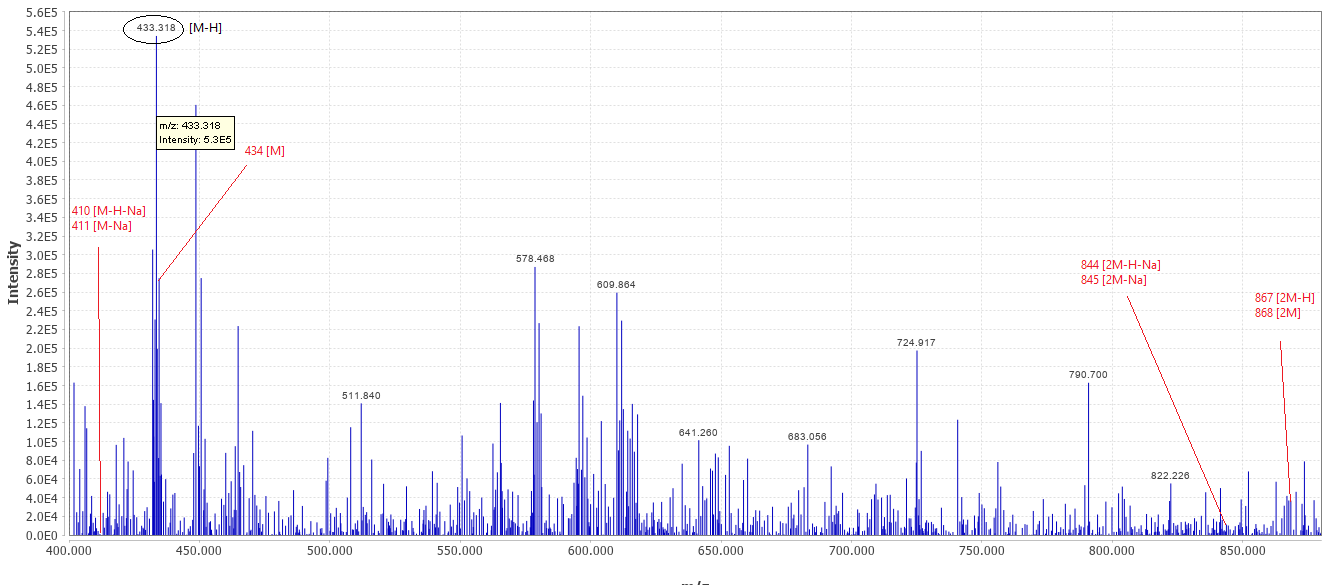

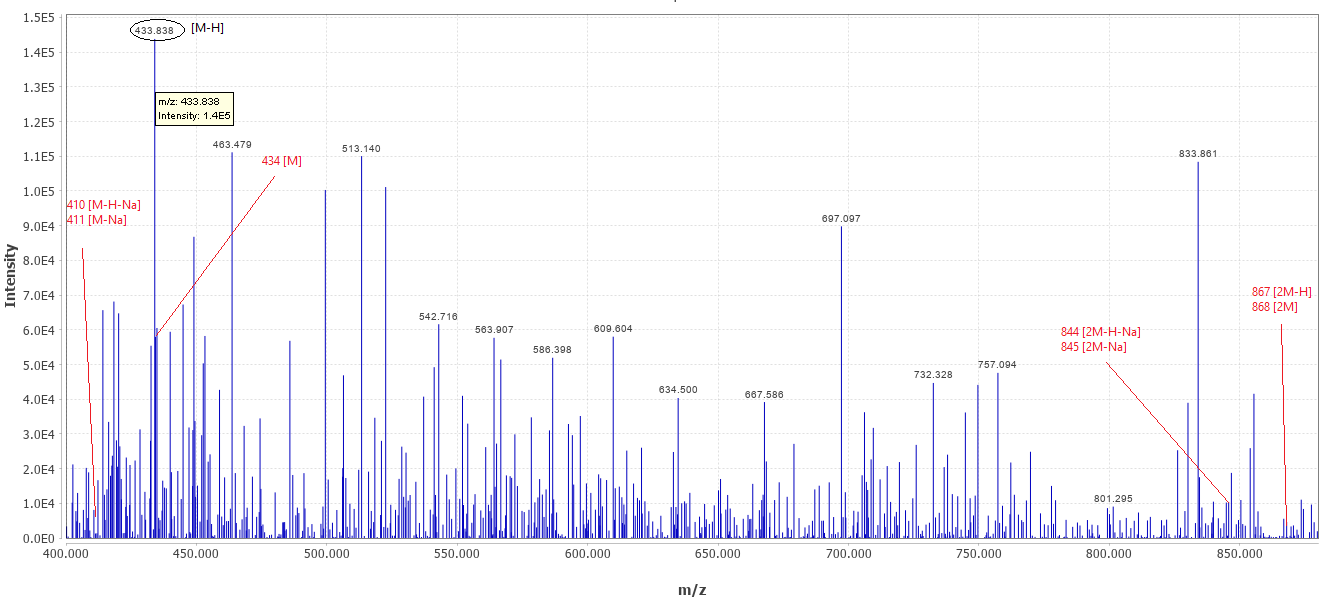

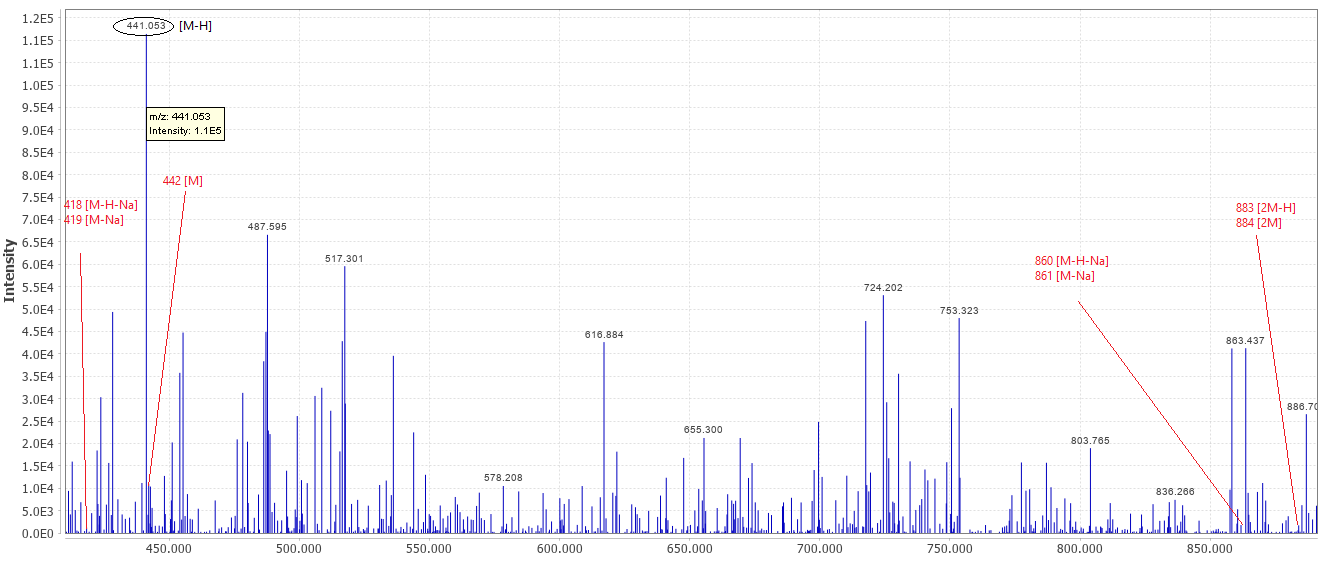

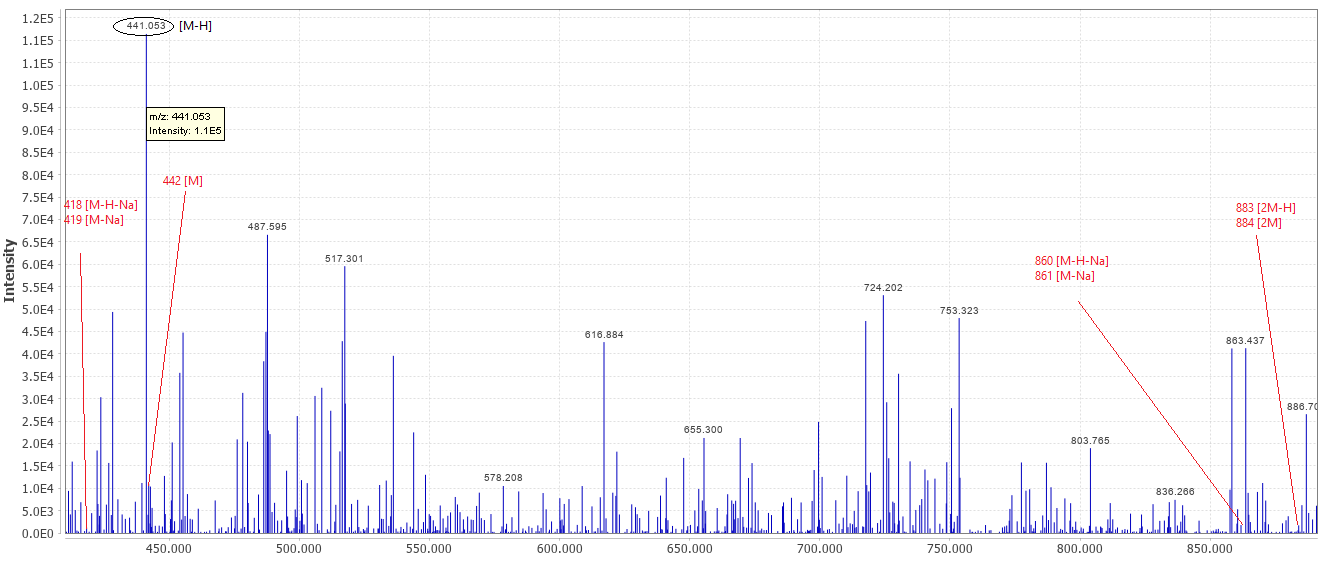

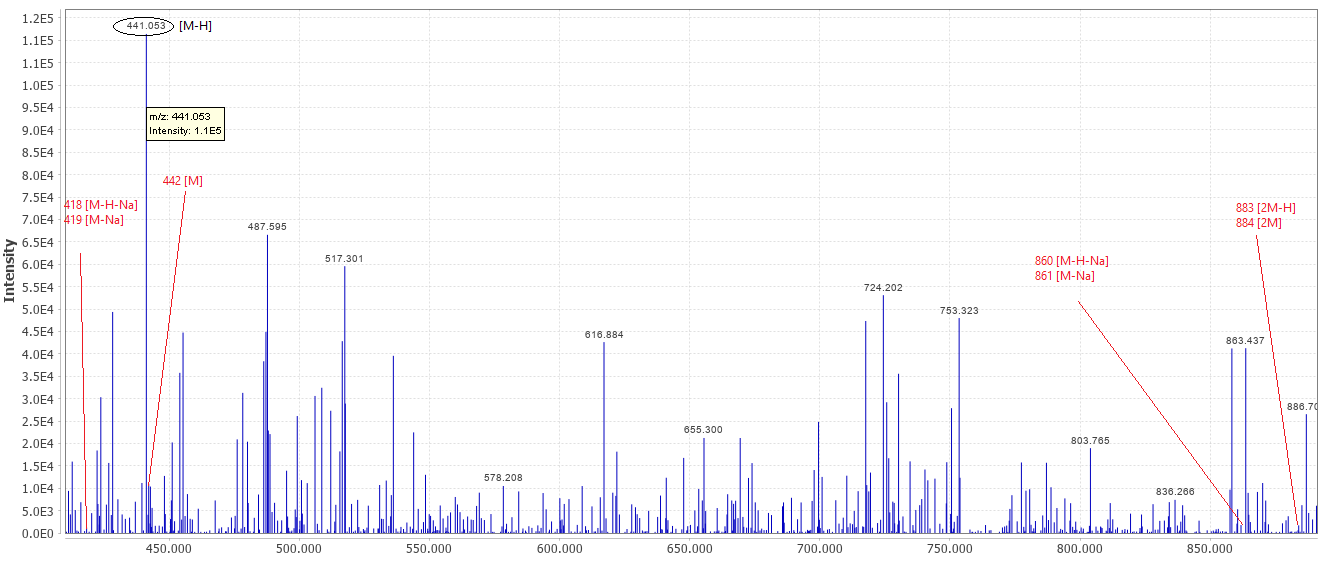

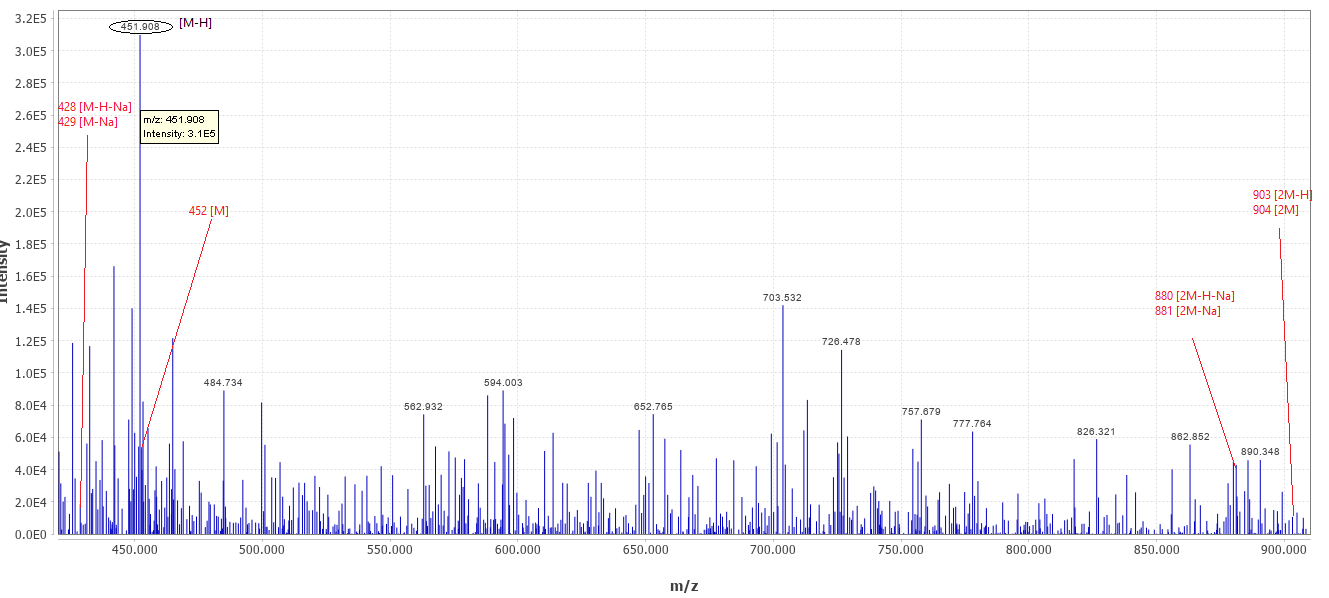

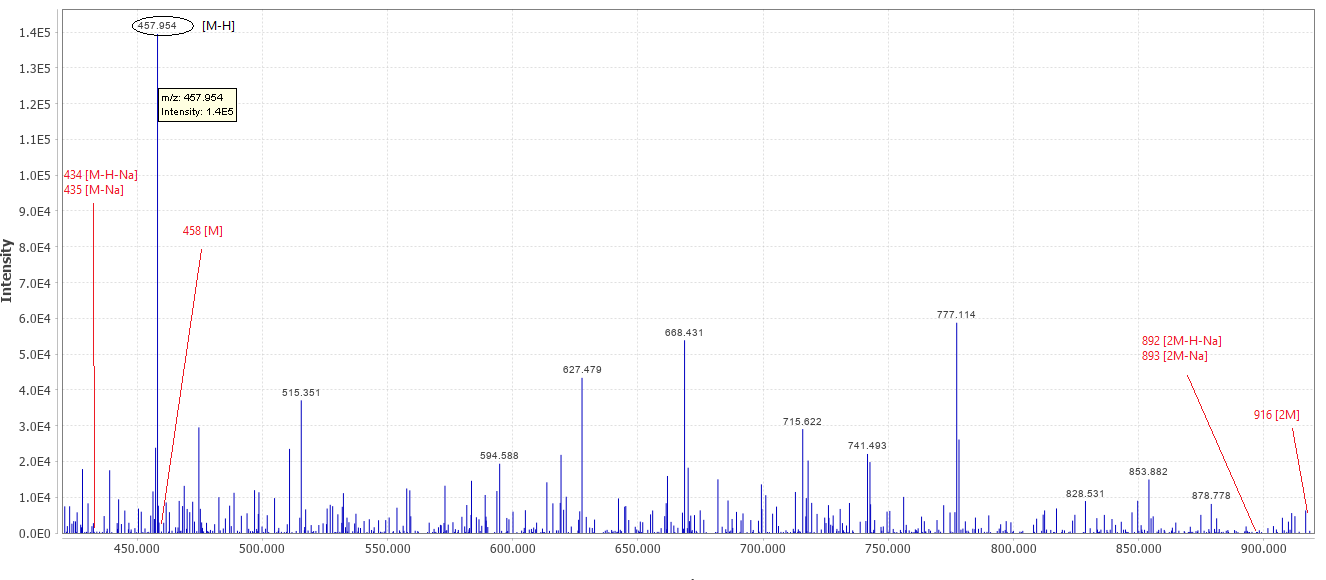

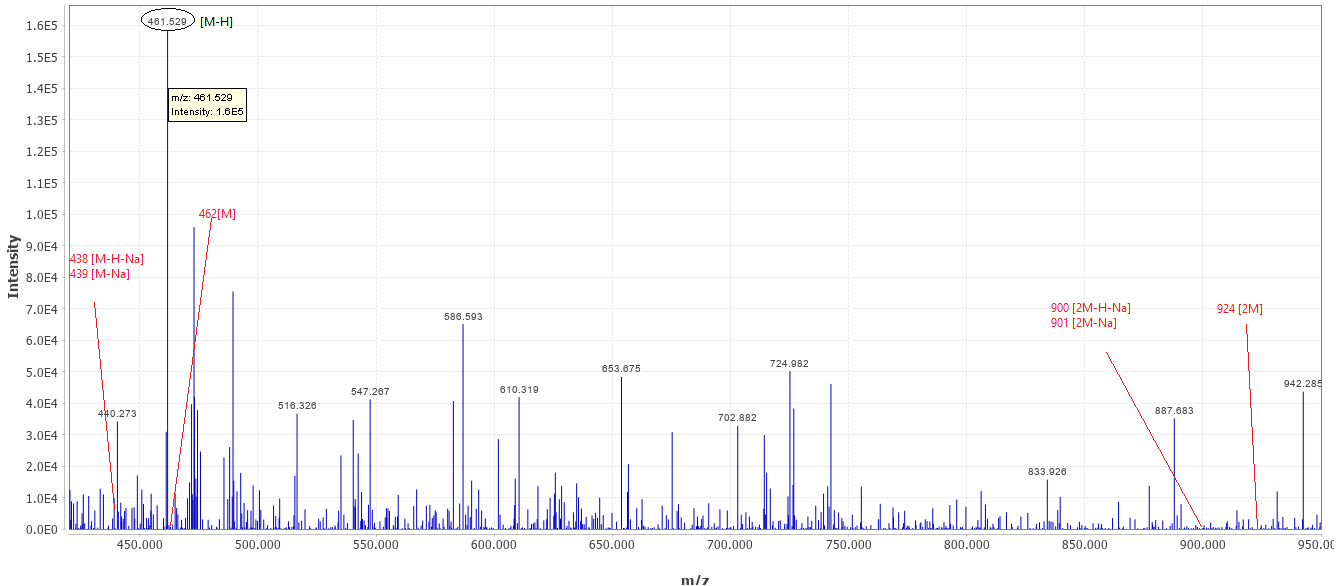

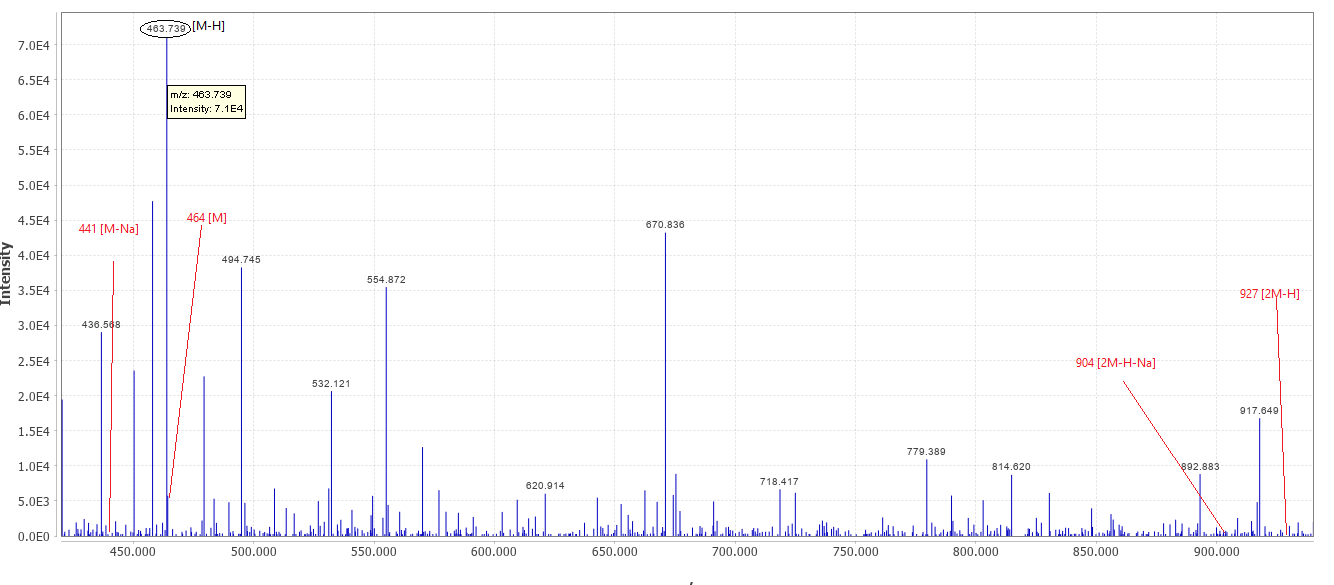

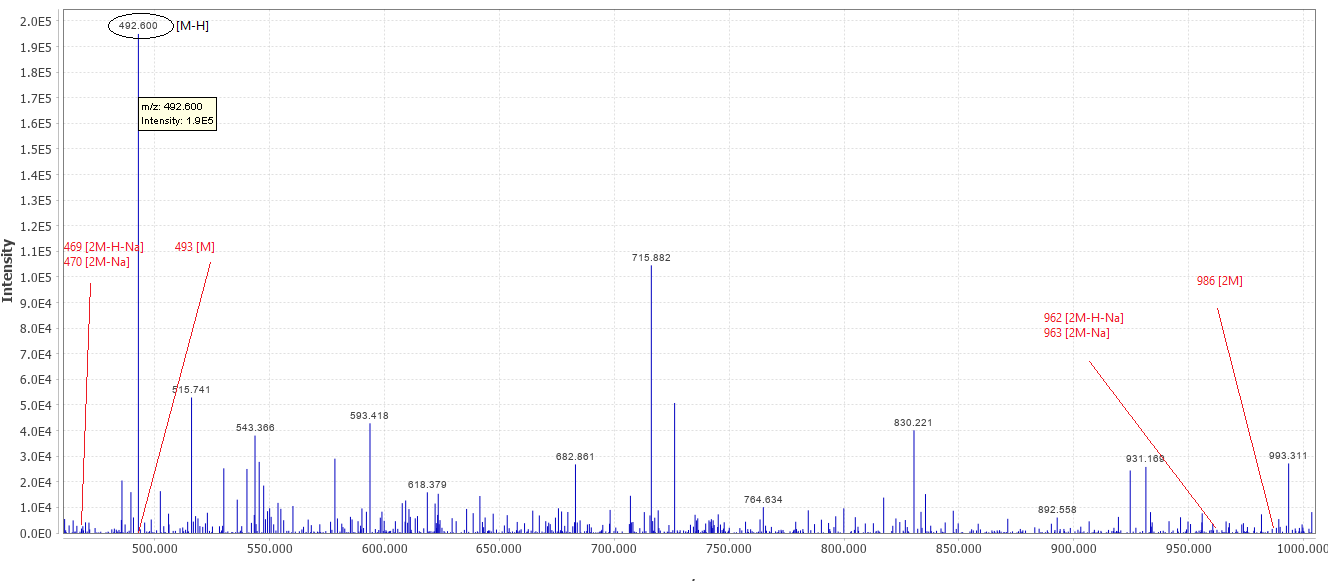

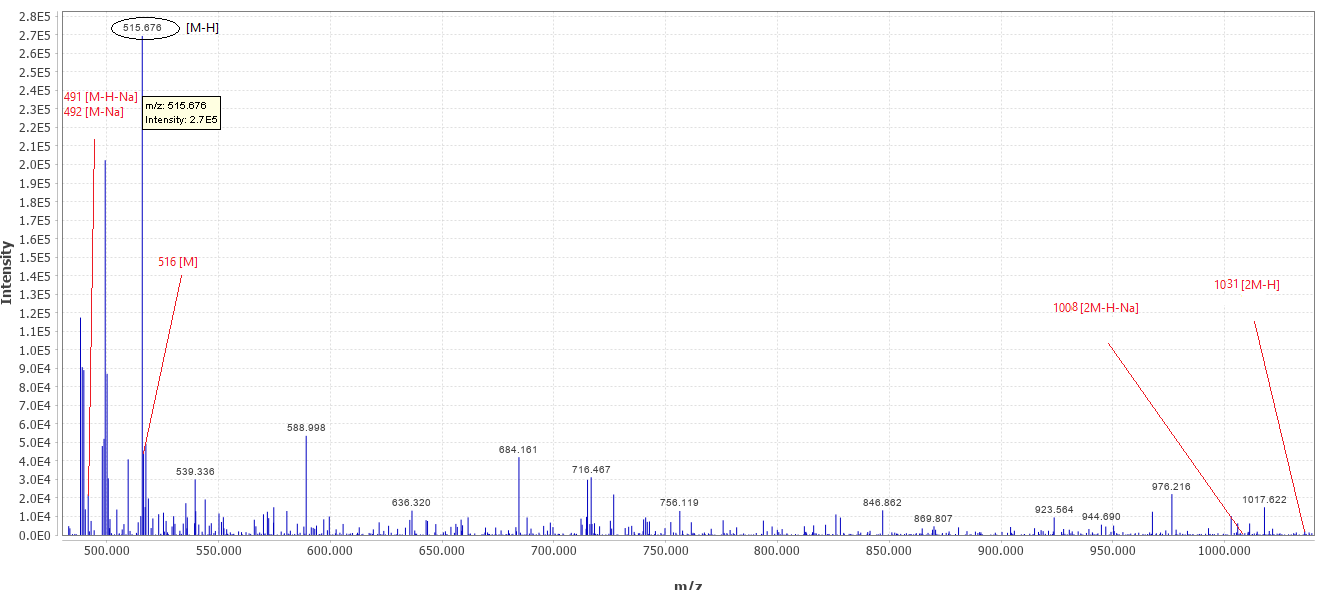

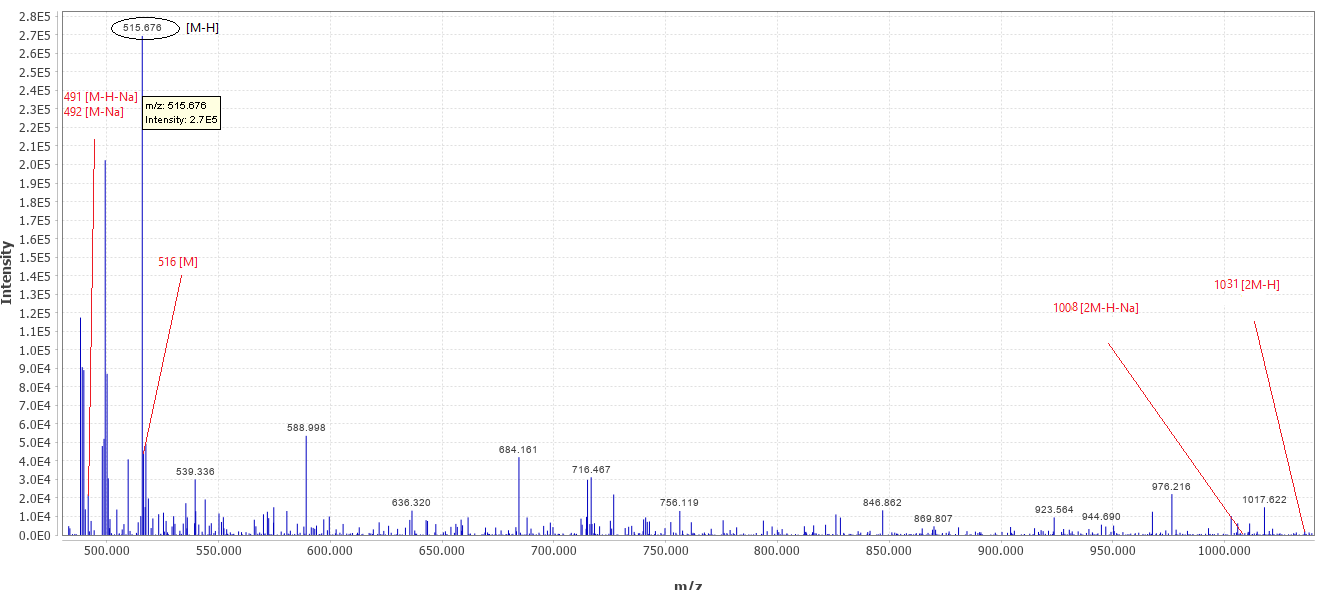

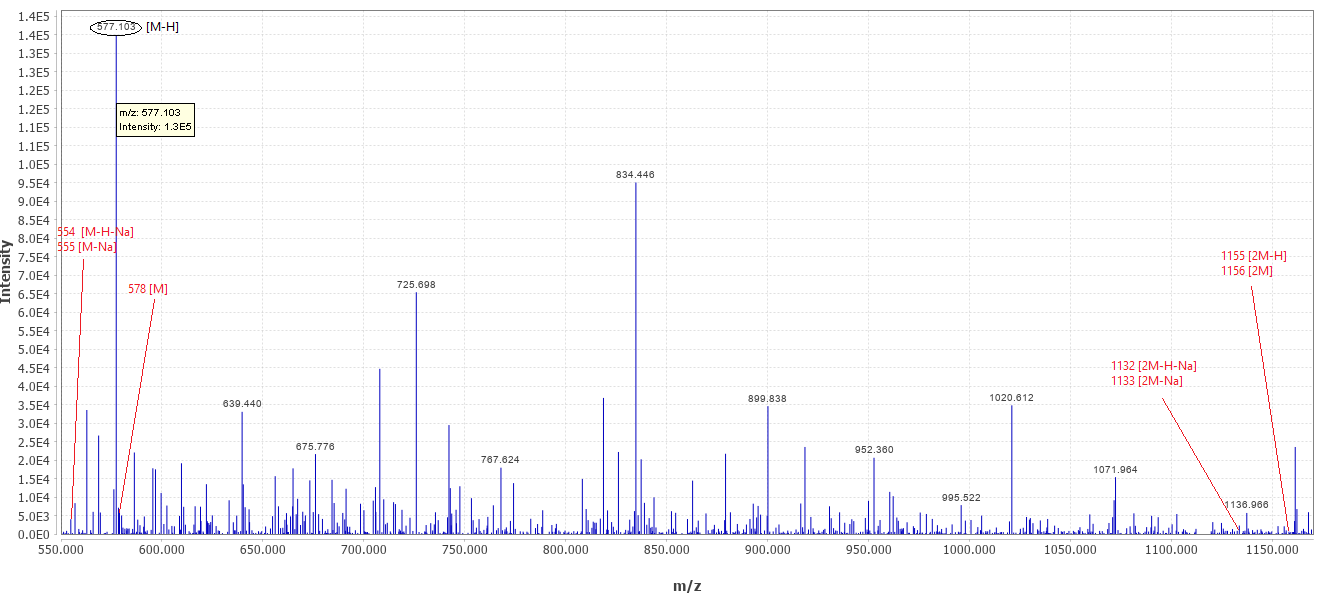

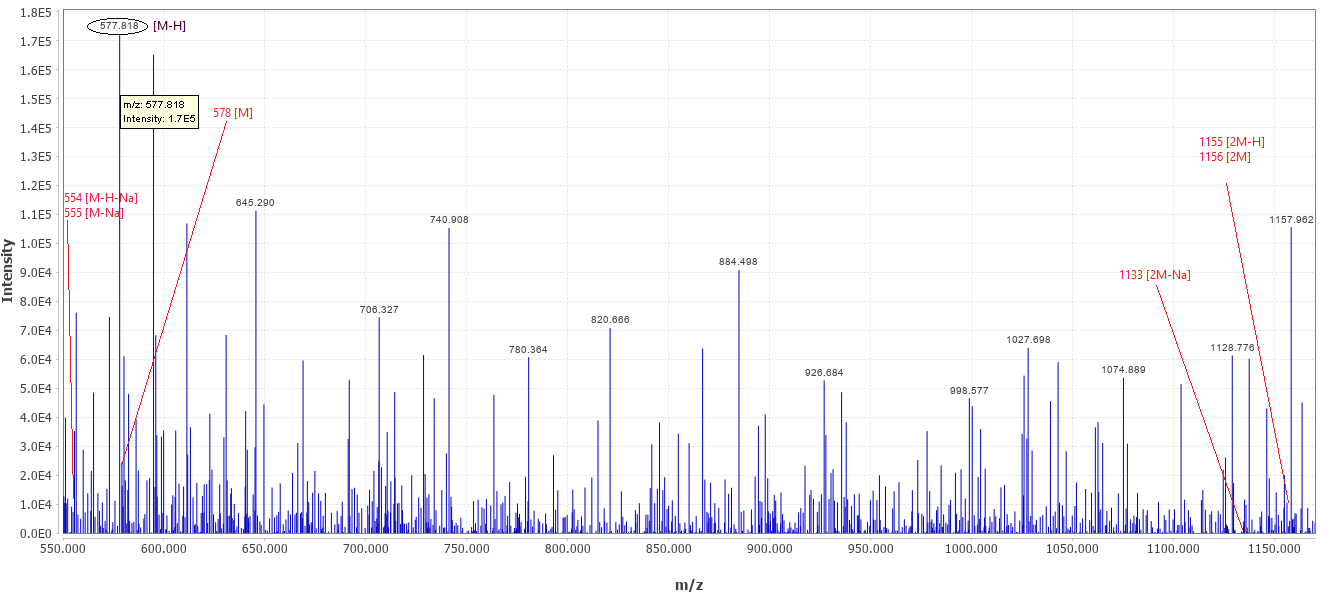

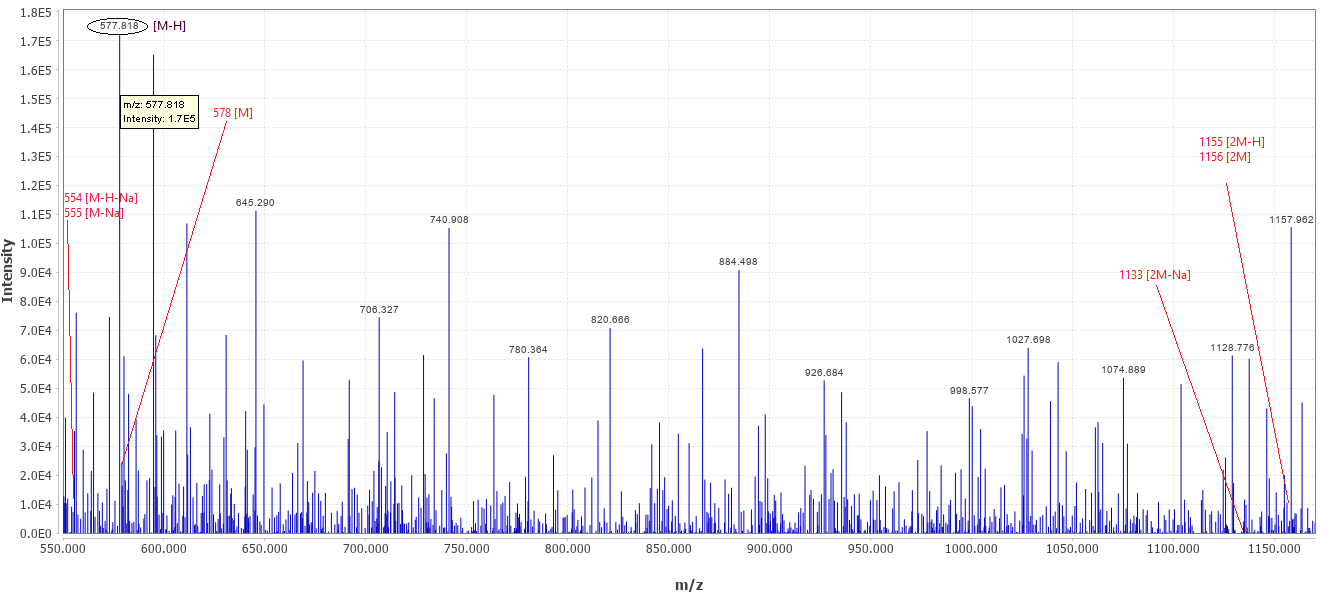


**Fig S4.** Mass spectra and corresponding adducts in all identified compounds of hydro-methanolic extract of *C.pentagyna* leaf.

**Fig S5.** Mass spectra and corresponding adducts in all identified compounds of hydro-methanolic extract of *C.pentagyna* root.
